# omicSynth: An open multi-omic community resource for identifying druggable targets across neurodegenerative diseases

**DOI:** 10.1016/j.ajhg.2023.12.006

**Published:** 2024-01-04

**Authors:** Chelsea X. Alvarado, Mary B. Makarious, Cory A. Weller, Dan Vitale, Mathew J. Koretsky, Sara Bandres-Ciga, Hirotaka Iwaki, Kristin Levine, Andrew Singleton, Faraz Faghri, Mike A. Nalls, Hampton L. Leonard

**Affiliations:** 1 Center for Alzheimer’s and Related Dementias (CARD), National Institute on Aging and National Institute of Neurological Disorders and Stroke, National Institutes of Health, Bethesda, MD 20814, USA; 2 Data Tecnica LLC, Washington, DC 20037, USA; 3 Laboratory of Neurogenetics, National Institute on Aging, National Institutes of Health, Bethesda, MD 20814, USA; 4 Department of Clinical and Movement Neurosciences, UCL Queen Square Institute of Neurology, London WC1N 3BG, UK; 5 UCL Movement Disorders Centre, University College London, London WC1N 3BG, UK; 6 German Center for Neurodegenerative Diseases (DZNE), Tübingen, Germany

**Keywords:** neurodegeneration, druggability, summary-data-based Mendelian randomization, SMR

## Abstract

Treatments for neurodegenerative disorders remain rare, but recent FDA approvals, such as lecanemab and aducanumab for Alzheimer disease (MIM: 607822), highlight the importance of the underlying biological mechanisms in driving discovery and creating disease modifying therapies. The global population is aging, driving an urgent need for therapeutics that stop disease progression and eliminate symptoms. In this study, we create an open framework and resource for evidence-based identification of therapeutic targets for neurodegenerative disease. We use summary-data-based Mendelian randomization to identify genetic targets for drug discovery and repurposing. In parallel, we provide mechanistic insights into disease processes and potential network-level consequences of gene-based therapeutics. We identify 116 Alzheimer disease, 3 amyotrophic lateral sclerosis (MIM: 105400), 5 Lewy body dementia (MIM: 127750), 46 Parkinson disease (MIM: 605909), and 9 progressive supranuclear palsy (MIM: 601104) target genes passing multiple test corrections (p_SMR_multi_ < 2.95 × 10^−6^ and p_HEIDI_ > 0.01). We created a therapeutic scheme to classify our identified target genes into strata based on druggability and approved therapeutics, classifying 41 novel targets, 3 known targets, and 115 difficult targets (of these, 69.8% are expressed in the disease-relevant cell type from single-nucleus experiments). Our novel class of genes provides a springboard for new opportunities in drug discovery, development, and repurposing in the pre-competitive space. In addition, looking at drug-gene interaction networks, we identify previous trials that may require further follow-up such as riluzole in Alzheimer disease. We also provide a user-friendly web platform to help users explore potential therapeutic targets for neurodegenerative diseases, decreasing activation energy for the community.

## Graphical abstract

**Figure:**
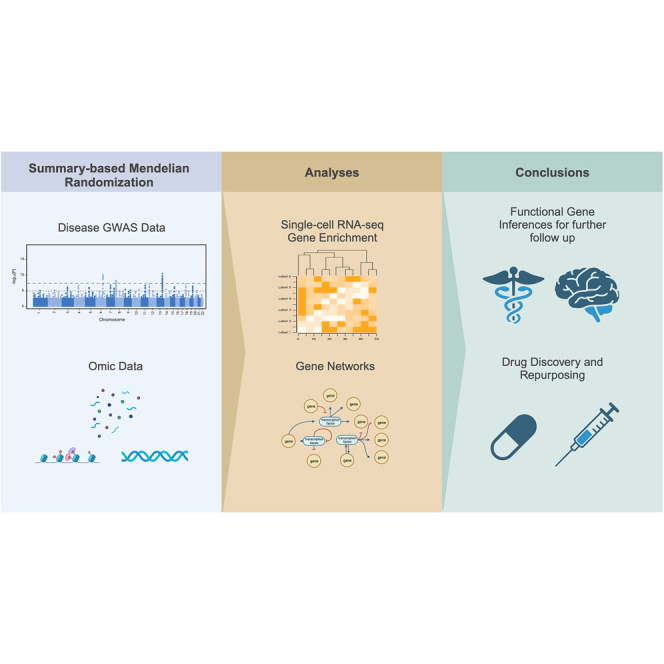

## Teaser

Neurodegenerative diseases (NDDs) affect millions of people, and finding genetically supported and effective drug targets has been difficult. Omic data availability and statistical methods can help nominate potential drug targets. We identified 54 NDD-related genes for further functional follow up and drug repurposing.

## Introduction

Currently, there are few approved disease-modifying therapeutics available to those with a neurodegenerative disease (NDD), the most recent being lecanamab for the treatment of Alzheimer disease.^1^ NDDs such as Alzheimer disease (AD), Parkinson disease (PD), amyotrophic lateral sclerosis (ALS), Lewy body dementia (LBD), frontotemporal lobar degeneration (FTLD [MIM: 607485]), and progressive supranuclear palsy (PSP) are diseases caused by progressive nerve cell degeneration that result in a loss of cognition and/or motor function.^2^ The World Health Organization (WHO) expects dementia diagnoses alone to reach 78 million by 2030 and 139 million by 2050. Without disease-modifying therapies, the health, social, and economic impacts of dementia and related NDDs will be catastrophic.^3^ The identification of rational therapeutic targets for NDDs will require both the generation of new data and the development and deployment of rapid, open, and transparent tools.

Drugs that are supported by genetic or genomic data frequently outperform those without such evidence in clinical trials. Over two-thirds of the US Food and Drug Administration (FDA)-approved drugs in 2021 were supported by genetic or genomic evidence.^4^ Therapeutics with genetically supported target mechanisms are twice as likely to pass a trial phase as those without supporting genetic data are.^5^ Given the importance of anchoring therapeutic targets to a disease mechanism substantiated by genetic evidence, we developed omicSynth: a dynamic, open, and accessible resource that leverages large-scale genetic and genomic data for the identification of therapeutic targets in the NDD space.

The omicSynth resource integrates genetic and genomic data in a summary-data-based Mendelian randomization (SMR) framework.^5^ The SMR framework facilitates functional inferences relating disease risk (from genome-wide association studies [GWASs]) to the underlying mechanism (from quantitative trait loci [QTL] variant data in relevant tissues) through their approach to Mendelian randomization (MR). MR uses instrumental variables (genetic variants) to test for a causative effect of an exposure, such as gene expression, on an outcome (disease phenotype). The SMR approach to MR tests for pleiotropic association between the exposure and outcome. Pleiotropic association is defined by Zhu et al. as the association between an outcome (disease phenotype) and exposure (QTLs) due to either pleiotropy (both QTLs and the disease phenotype are affected by the same causal variant) or causality (the effect of a causal variant on the disease phenotype is mediated by the QTLs). In order to distinguish pleiotropy from linkage, the accompanying heterogeneity in dependent instruments (HEIDI) method utilizes *cis*-QTLs surrounding the probe being tested to distinguish pleiotropy from linkage. The resulting SMR effect size (beta) and HEIDI values measure the effect of the exposure on the outcome free of any non-genetic confounders and the probability of the tested genetic variant being consistent with linkage, respectively.^5^ In an SMR association as shown here, the effect estimates correspond to functional inferences where a positive association suggests concordance between increased omic levels and increased risk and a negative beta suggests the inverse. In many analyses, the same associations can have opposite directions for the same gene in different tissues or in different disease contexts. The data utilized are GWASs from population-scale resources, composed of millions of samples across multiple NDDs, and omic data composed of QTL studies measuring methylation, gene expression, chromatin state, and protein expression. Additionally, we incorporated expression-QTL (eQTL) data from genetically diverse backgrounds into our SMR analyses, addressing the lack of multi-ancestry data in NDD research and allowing for limited functional inferences regarding differences between ancestral populations.

To add additional context to nominated gene targets, we also investigated single-nucleus data from disease-enriched cell types.^6^^,^^7^ Many therapeutics mechanistically target inhibition of expression, thus, it is necessary that the target is expressed at baseline in the relevant cell types for these treatments to be effective in addressing a particular disease. Using single-nucleus expression data, we investigated whether the nominated genes could potentially be affected by inhibitors that can cross the blood-brain barrier, as in general, genes need to be expressed in order to be susceptible to inhibition from a mechanistic perspective.

**Figure 1:**
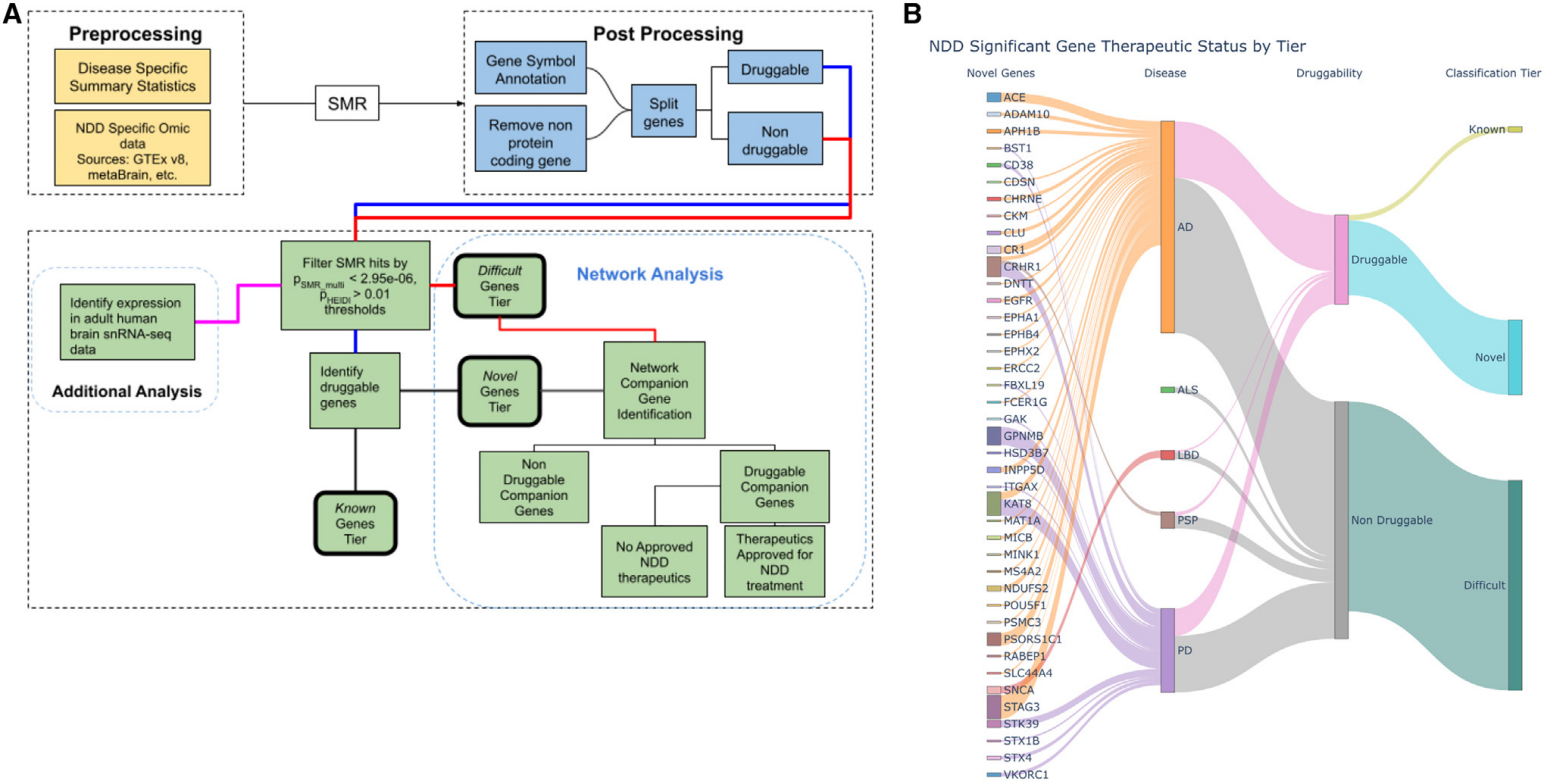
Graphical representation of research workflow and results (A) Graphical representation of the general workflow used in conducting our analyses. NDD, neurodegenerative disease; SMR, summary-data-based Mendelian Randomization. (B) Graphical summary of results. Sankey plot depicting the flow of candidate genes into their respective tier. On the left, we highlight novel genes, but the remainder of the plot visualizes all 159 candidate genes regardless of the final classification tier. Note: the size of each novel gene node is scaled to represent the number of significant SNPs for the associated gene probe-disease pairing.

We prioritize identified genes as therapeutic targets of interest into three classes based upon known small-molecule druggability and product market information (Figure 1). Novel targets include genes that exhibit significant functional inferences in relevant tissue and cell types in druggable regions of the genome, are not currently targeted by disease-specific therapeutics, and should be prioritized in future repurposing studies and drug development. Known targets include genes within relevant tissue and cell types that have documented significant functional inferences but are already impacted by a known drug that specifically targets an NDD. Difficult target genes are not in regions of the genome currently annotated as druggable. For all novel targets, we searched the corresponding gene regulatory networks to identify companion genes that could also be useful as therapeutic targets. Potential upstream and downstream effects on targeting these genes for therapeutic intervention were provided based on network memberships, and toxicity within these networks was inferred by evaluating liver eQTLs within the network as well as known interacting drugs for each gene of interest. Results can be browsed through the omicSynth resource, which is made available via a free web-based platform, further decreasing activation energy for therapeutic target discovery within the research community (https://nih-card-ndd-smr-home-syboky.streamlit.app/).

## Material and methods

### Datasets

GWAS summary statistics for each of the six NDDs highlighted in our study were used to obtain single nucleotide polymorphisms (SNPs) that served as instrumental variables in the MR pipeline. GWASs used are the latest and/or largest for each corresponding disease: Bellenguez et al. for AD (n = 788,989); Chia et al. for LBD (n = 7,372); Höglinger et al. for PSP (n = 4,361); Nalls et al. for PD (n = 1,456,306); Nicolas et al. for ALS (n = 80,610); and Pottier et al. for FTLD (n = 1,355).^8^^,^^9^^,^^10^^,^^11^^,^^12^^,^^13^All GWAS summary statistics were lifted over, as needed, to hg19 (GRCh37) using University of California, Santa Cruz’s liftOver command line tool.^14^

### QTL summary statistics

eQTLs, protein QTLs (pQTLs), chromatin QTLs (caQTLs), and methylation QTLs (mQTLs) were used as the exposure variables in the MR analyses. eQTLs are genetic loci that explain the variation in mRNA expression levels. *Cis*-eQTL, eQTLs that act on local genes, data make up most all QTL data used for our study because of the volume of publicly available data sources. All eQTL and mQTL data obtained, except from the sources eQTLgen, metaBrain, and Zeng et al. (multi ancestry), were already in SMR format and obtained from the Yang Lab’s Data Resource page.^15^^,^^16^^,^^17^ The eQTL sources from the Yang Lab include Genotype-Tissue Expression (GTEx) project v8 release, PsychENCODE, and BrainMeta v1 (formerly brain-eMeta).^18^^,^^19^^,^^20^ The specific tissues measured varied by data source but consisted of NDD-related tissues, which we have defined as brain, nerve, muscle, blood, and liver tissues. Liver was included because of its role in metabolizing medications, toxicity, and potential impacts on clinical trial progress.^21^

mQTLs are genetic variants that affect methylation patterns of CpG sites. mQTL data sources include Brain-mMeta and McRae et al., which are derived from blood tissues.^20^^,^^22^ We included caQTLs—caQTLs alter traits by modifying chromatin structure—data from Bryois et al. in our analysis.^23^ Blood tissues have been shown to have high correlation in expression levels with brain tissues, allowing blood tissues to provide a gain of power and ease of use in biomarker studies due to the relative ease of availability of this tissue.^20^ All genome positions are mapped to the human reference genome build hg19 (GRCh37).

pQTLs are genetic variants associated with protein expression levels. Similarly to eQTLs and mQTLs, pQTLs can be used as our exposure variable. We obtained pQTL summary statistics data from Yang et al.^24^ The pQTLs are from plasma, brain, and cerebrospinal fluid (CSF) tissues from participants with and without AD. Samples are on human reference genome build hg19 (GRCh37). More details on the samples and methods used can be found in the original manuscript.^24^ pQTLs were nominated for inclusion for SMR analysis if they were significant (p < 0.05) in at least one of the three tissues per the original manuscript. We included 453 unique pQTLs across the three tissues: 223 pQTLs from CSF, 159 from plasma, and 77 from the brain.

### Gene expression summarization

We summarized expression ranks for genes of interest within the single-cell adult human brain transcriptome dataset adult_human_20221007.loom from Siletti et al.^6^ Using custom R scripts, we converted feature counts into TPM (transcripts per million). For a given sample, feature counts were divided by maximum nonredundant intron-removed exon lengths to correct for differences in gene length. Values were then multiplied by a sample-specific constant (10^6^/T, where T is the sum of length-normalized counts) such that the resulting unitless vector sums to one million. We extracted exon lengths based on annotations from the gene transfer format (GTF) file used to originally annotate the single-cell data (gb_pri_annot.gtf). We calculated the expression percentile rank (EPR) for genes of interest using the empirical cumulative distribution function and then calculated the mean and median EPR value for each gene across cells of each tested cell type. To ease interpretation, we binned the EPR values into 3 classes—off, low, and high (off: EPR <10, low: 10 < EPR <90, high: EPR >90) by using the mean single-nucleus RNA sequencing (snRNA-seq) EPR of each gene against cell type.

### Gene-gene networks

Data were obtained from the Open Targets.^25^ Open Targets provides an API to cross reference annotations and relationships on diseases, genes, and drugs. Companion genes were pulled from the SIGnaling Network Open Resource (SIGNOR) database due to the manual curation of gene interactions.^26^

### Therapeutic drug data

Therapeutic drug data were obtained from various sources including Finan et al. (“druggable genome”) and the Drug Gene Interaction Database (DGIdb).^27^^,^^28^ Druggable genome data were obtained from the supplementary materials in Finan et al. The obtained data provided insight on 3,000+ potential gene targets with evidence for drug targets or potential targets.^27^ DGIdb drug data (accessed January 2023) were downloaded from the DGIdb online database as files consisting of known gene and drug interactions as well as details such as interaction types and drug categories.

### Pre-processing

All data pre-processing was carried out using custom scripts for data that were not obtained via the Yang Lab or was missing information such as gene symbols. Pre-processing included gene annotation, binary with expression summary data (BESD) format preparation and conversion, and/or calculation of necessary measures such as beta values. Gene annotation was performed as necessary if no gene symbol was provided in the original data source. Annotation was conducted by using both the Python biomart package and pyensembl with Ensembl gene (ENSG) IDs. mQTL gene annotation was conducted by obtaining Illumina 450k chip probe data via the R package IlluminaHumanMethylation450kanno.ilmn12.hg19.^29^^,^^30^^,^^31^

Data sources were converted as necessary into BESD format using the flist method outlined by the Yang Lab. BESD format stores QTL summary data in a set of three files: .esi, .epi, and .besd. More information on the format and how to process data into BESD format can be found on the SMR Yang Lab website listed in the key resources table.

Our multi-ancestry eQTL data originally lacked allele frequency, beta, or standard error values. Missing allele frequencies were obtained using the 1000 Genomes reference panel from which we derived beta and standard error values using each eQTL’s random effects model *Z* score, allele frequency, and total number of samples from the original study (n = 2,119).

### Summary-data-based Mendelian randomization

SMR is an MR computational tool that uses summary-level data to test if an exposure variable (i.e., gene expression) and outcome (i.e., disease phenotype) are causally associated because of a shared causal variant (i.e., instrumental variable).^5^ To discern potentially causal variants from those in linkage disequilibrium with the functional variant, the HEIDI method was implemented using the default flag that uses the top 20 SNPs within 500 kb of the probe.^5^ Linkage disequilibrium reference data were obtained from 1000 Genomes Project phase 3 reference panel.^32^ To increase statistical power, we applied the SMR-multiple exposures (SMR-multi) method, a Bayesian framework for simultaneous testing of multiple traits or exposures on a single outcome while accounting for the correlation between them. SMR and HEIDI analysis were conducted using the SMR software established and maintained by the Yang Lab using all default parameters, including those previously detailed.^5^^,^^33^

Following SMR, we filtered results to include only protein-coding genes and removed potential associations with no available gene annotations or associations with genes in the major histocompatibility complex (MHC). We used a significance threshold of p_SMR_multi_ < 2.95 × 10^−6^, corresponding to the Bonferroni-corrected value at α = 0.05 for 16,875 protein-coding genes tested across all NDDs and omic pairs. We then filtered results based on the presence of inferred pleiotropy via the computed HEIDI score (p_HEIDI_ > 0.01 for inclusion in this study).^5^ SNPs were then split on their associated genes status as a therapeutic target or as a non-therapeutic target. After initial processing, analyses were conducted as demonstrated in our workflow diagram (Figure 1A) and explained further in our gene nomination workflow below. In total, we tested a total of 186 omic-tissue pairs across six NDDs (Table 1).

**Table 1:** Summary of SMR data mining across NDDs

**Disease**	**Total genes (unique)**	**Liver genes**	**Total eQTL genes (non-multi-ancestry)**	**Replicated in multi-Ancestry**	**Total druggable genes**	**% Druggable**	**Total non-druggable genes**	**% Non-druggable**
**All tested genes (protein coding)**								

AD	16,833	1,597	15,112	8,404	3,562	21.2%	13,271	78.8%
ALS	16,875	1,610	15,163	8,408	3,565	21.1%	13,310	78.9%
FTLD	16,788	1,537	15,038	8,394	3,551	21.2%	13,237	78.8%
LBD	16,797	1,540	15,069	8,388	3,554	21.2%	13,243	78.8%
PD	16,872	1,596	15,159	8,407	3,566	21.1%	13,306	78.9%
PSP	16,042	1,033	13,839	8,073	3,420	21.3%	12,622	78.7%

**Significance p_**_**SMR_multi**_**< 0.05 and**_**p_HEIDI**_**> 0.01**								

AD	8	175	3,189	2,079	1,142	14,275.0%	3,806	47,575.0%
ALS	3,188	83	1,857	1,260	715	22.4%	2,473	77.6%
FTLD	2,318	78	1,243	810	542	23.4%	1,776	76.6%
LBD	2,530	82	1,384	900	580	22.9%	1,950	77.1%
PD	3,592	108	2,161	1,434	811	22.6%	2,781	77.4%
PSP	2,275	30	1,270	842	574	25.2%	1,701	74.8%

**Significance p_**_**SMR_multi**_**< 2.95E-06 (testing all protein coding genes) and p**_**_HEIDI**_**> 0.01**								

AD	116	2	68	7	31	26.7%	85	73.3%
ALS	3	0	3	0	0	0.0%	3	100.0%
FTLD	0	0	0	0	0	0.0%	0	0.0%
LBD	5	0	1	0	1	20.0%	4	80.0%
PD	46	3	33	5	15	32.6%	31	67.4%
PSP	9	0	5	3	2	22.2%	7	77.8%

**Significance p_**_**SMR_multi**_**< 1.58E-08 (testing all protein coding genes across all omics) & p_**_**HEIDI**_**> 0.01**								

AD	47	1	19	19	14	29.8%	33	70.2%
ALS	1	0	0	0	0	0.0%	1	100.0%
FTLD	0	0	0	0	0	0.0%	0	0.0%
LBD	2	0	0	0	1	50.0%	1	50.0%
PD	24	1	14	14	8	33.3%	16	66.7%
PSP	8	0	5	5	2	25.0%	6	75.0%

AD, Alzheimer disease; ALS, amyotrophic lateral sclerosis; FTLD, frontotemporal dementia lobar degeneration; LBD, Lewy body dementia; PD, Parkinson disease; PSP, progressive supranuclear palsy.

### Gene nomination and drug target identification

Gene nomination focused on targets shared by multiple NDDs, classifying targets by inferred druggability as described in the introduction. Therapeutic targets were initially nominated using data from DGIdb and Finan et al.^27^^,^^28^ Further target curation was conducted using Open Targets to verify if any approved indications included an NDD thus allowing us to classify drugs into either the novel or known tiers. Identified network companion genes upstream and downstream of the initial target identified were further categorized into groups based on therapeutic status and approved use in treating any NDD. Our known and difficult tiers were further investigated using gene co-expression networks via Open Targets interaction annotations through the SIGNOR database. Using SIGNOR-identified interactions, we identified companion genes, i.e., those manually annotated for their causal relationships with the gene of interest. We additionally searched known therapeutics that target any identified companion genes to potentially identify proxy gene targets thus expanding the net for drug discovery and repurposing. We implemented custom python scripts to query Open Targets’ application programming interface (API) to extract relevant annotations for this workflow.

## Results

### Overview

We identified 540 candidate gene-level SMR associations (159 unique gene targets) across six NDDs and 186 tissue-omic pairings with a stringent disease-level multiple test correction threshold (p_SMR_multi_ < 2.95 × 10^−6^ and p_HEIDI_ > 0.01; Tables S1, S2, and S4). On a per-disease basis we identified 317 total significant associations: 116 unique gene targets for AD, 4 significant associations across 3 unique gene targets for ALS, 13 significant associations across 5 unique genes for LBD, 184 significant associations across 46 unique gene targets for PD, and 22 significant associations across 9 unique gene targets for PSP. FTLD had no significant associations at our corrected p value threshold. No NDDs showed significant pQTL or caQTL associations. Of the 159 unique genes across all diseases, 69.8% (111 genes) were expressed in tissues enriched with disease-associated gene expression, and 11.9% (19 genes) showed colocalization with brain tissue eQTLs with posterior probability (PP) >0.90.^7^ Colocalized genes from this complementary analysis are also identified in Tables 2 and 3.

**Table 2:** Candidate genes for multiple NDDs

**Gene**	**Diseases**	**Omics**
ARL17B	AD, PD	cerebellum eQTL, cortex eQTL, spinalcord eQTL
KAT8	AD, PD	cerebellum eQTL, whole-brain meta-analysis mQTL, cerebellar hemisphere eQTL, cortex eQTL, tibial nerve eQTL, skeletal muscle eQTL, hypothalamus eQTL, whole-brain eQTL, cerebellum eQTL, spinalcord eQTL
LRRC37A2	AD, PD	hippocampus eQTL, cortex eQTL, frontal cortex BA9 eQTL, prefrontal cortex eQTL, caudate basal ganglia eQTL, skeletal muscle eQTL, multi-ancestry, whole-brain meta-analysis eQTL, hypothalamus eQTL, liver eQTL, anterior cingulate cortex BA24 eQTL, putamen basal ganglia eQTL, amygdala eQTL, whole-brain eQTL, cerebellum eQTL, nucleus accumbens eQTL, basal ganglia eQTL, spinalcord eQTL, hippocampus eQTL, substantia nigra eQTL
KANSL1	AD, PD, PSP	whole-brain meta-analysis mQTL, whole-blood mQTL, cortex eQTL, multi-ancestry whole-brain meta-analysis eQTL, spinalcord eQTL, anterior cingulate cortex BA24 eQTL
ARL17A	AD, PD, PSP	spinalcord eQTL, amygdala eQTL, multi-ancestry whole-brain meta-analysis eQTL, hypothalamus eQTL, hippocampus eQTL, cerebellar hemisphere eQTL, cortex eQTL, caudate basal ganglia eQTL, anterior cingulate cortex BA24 eQTL, putamen basal ganglia eQTL, cerebellum eQTL, nucleus accumbens basal ganglia
PRSS36	AD, PD	whole-brain meta-analysis mQTL, cortex eQTL, cerebellar hemisphere eQTL, multi-ancestry whole-brain meta-analysis eQTL, whole-brain eQTL
MAPT	AD, PD, PSP	whole-brain meta-analysis mQTL, whole-blood mQTL
IDUA^∗^	LBD, PD	whole-brain meta-analysis mQTL, whole-blood mQTL, whole-blood eQTL(eQTLgen)
TMEM175^∗^	LBD, PD	whole-blood mQTL
ARHGAP27	AD, PD, PSP	whole-blood mQTL, whole-blood eQTL (eQTLgen), multi-ancestry whole-brain meta-analysis eQTL, caudate basal ganglia eQTL, nucleus accumbens basal ganglia
CRHR1	AD, PD, PSP	whole-brain meta-analysis mQTL, whole-blood mQTL, cortex eQTL, skeletal muscle eQTL
FMNL1	AD, PSP	multi-ancestry whole-brain meta-analysis eQTL, whole-blood mQTL
PLEKHM1	PD, PSP	cortex eQTL, frontal cortex BA9 eQTL, prefrontal cortex eQTL, caudate basal ganglia eQTL, skeletal muscle eQTL, anterior cingulate cortex BA24 eQTL, putamen basal ganglia eQTL, whole-brain eQTL
WNT3	AD, PD	cortex eQTL metaBrain, skeletal muscle eQTL, tibial nerve eQTL
SPPL2C	AD, PD	cerebellum eQTL, prefrontal cortex eQTL

This table shows genes with functional inferences passing multiple test corrections for multiple neurodegenerative diseases. We provide details for all the omics and diseases in which a given gene has significant associations. Asterisks indicate colocalized genes.

**Table 3:** Therapeutic classification scheme by tier

Tier	**Requirements**	**Number of genes**	**Genes**
Novel	druggable; not approved for use in treating NDDs	41	ADAM10, SNCA, EGFR, POU5F1, STK39, INPP5D, CRHR1, APH1B, MINK1, CLU, CR1, ACE, CD38, RABEP1, ERCC2, KAT8, ITGAX, GAK, STX4, EPHB4, EPHA1, GPNMB, STAG3, CHRNE, NDUFS2, FCER1G, VKORC1, DNTT, CKM, HSD3B7, BST1, STX1B, PSMC3, CDSN, MICB, MS4A2, PSORS1C1, EPHX2, SLC44A4, MAT1A, FBXL19
Known	druggable; approved for use in treating NDD	3	MAPT, KCNN4, ADORA2B
Difficult	no known druggability	115	TRIM27, PPP4C, SPI1, EFNA3, KIF1C, WNT3, CD2AP^∗^, CCNE2, KCTD13, C9orf72^∗^, SRCAP, CELF1, HIP1R^∗^, GRN^∗^, APOC2, ARHGAP27, MEPCE, LRRFIP2, COPS6, GIGYF1, BCKDK, POLR2E, EFNA4, DYDC1, ATF6B, LLGL1, MTMR2, GPC2^∗^, LRRC37A, ARL17B, INO80E^∗^, SNX31, CEACAM19^∗^, DGKQ^∗^, NUP42, LRRC37A2, KANSL1, ARL17A, ANXA11, TSPAN14, CASTOR3, ZNF232, ZNF45, TSBP1, TREM2, PRSS36^∗^, IDUA^∗^, CCDC158, CCDC189, ZSWIM7, PLEKHM1, STH, PVRIG, YPEL3, MMRN1^∗^, SPPL2C, SCIMP, PILRB^∗^, PILRA, LACTB^∗^, FMNL1, APOC4, ZNF646, CPSF3, ZSCAN9, ZKSCAN3, TREML2, EPDR1, UFSP1, FAM131B, TAS2R60^∗^, USP6NL, MS4A4A, CASS4, G2E3, SCFD1, PCGF3^∗^, SETD1A, DCAKD, ZNF668, AGFG2, TMEM175^∗^, TOMM40, TRIM40, WDR81, TMEM106B, FNBP4, SHROOM3, CYP21A2^∗^, REXO1, TNXB, MS4A3, AIF1, RAB8B, ZFP57, FAM200B, BTNL2, IGSF9B^∗^, HS3ST1, ZNF311, NDUFAF6, TMEM163^∗^, APOC1, C17orf107, EXOC3L2, DYDC2, DOC2A, ACMSD, TRIM31, PRDM7, TRIM10, ZAN, MS4A6A, CPLX1, SFTA2

Table providing information on the three classifications tiers in our therapeutic classification scheme including requirements for each tier. Asterisks indicate colocalized genes.

### SMR analysis identifies 15 common genes significant across multiple NDDs

Using SMR, we identified 15 unique genes across 182 associations to be significant in two or more NDDs at a stringent significance threshold (p_SMR_multi_ < 2.95 × 10^−6^ and p_HEIDI_ > 0.01; Tables S2, S3, and S4). Of the identified genes, five genes (*MAPT* [MIM: 157140], *CRHR1* [MIM: 122561], *KANSL1* [MIM: 612452], *ARL17A*, and *ARHGAP27* [MIM: 610591]) were found to be significant across 97 tested associations and three NDDs (AD, PD, and PSP). *MAPT* and *CRHR1* were found to be largely significant in mQTL omics with *MAPT* significant in whole blood and brain mQTL data for all previously mentioned NDDs, and *CRHR1* was found to be significant in whole blood mQTL data for all three NDDs (Tables S3, S4, and S5). Additionally, only *MAPT* and *CRHR1* are annotated as druggable in multiple drug data sources as of the writing of this manuscript. All genes, except for *ARL17A*, had multiple significant associations in both brain and blood mQTL tissues. *ARHGAP27* and *KANSL1* had significant associations replicated in the multi-ancestry eQTL data (African, American, East Asian, European, and South Asian ancestries), suggesting a potential generalizability of these targets across different populations.

We identified 10 unique genes across 85 significant associations in any two NDDs (p_SMR_multi_ < 2.95 × 10^−6^ and p_HEIDI_ > 0.01). One of the identified genes is considered therapeutic, and the remaining nine are non-therapeutic. *KAT8* (MIM: 609912), *ARL17B*, *PRSS36* (MIM: 610560), *LRRC37A2* (MIM: 616556), *WNT3* (MIM: 165330), and *SPPL2C* (MIM: 608284) were all found to be significant in both AD and PD; *IDUA* (MIM 252800) and *TMEM175* (MIM: 616660) were found to be significant in LBD and PD; *PLEKHM1* (MIM: 611466) was significant in PD and PSP; and *FMNL1* (MIM: 604656) was significant in AD and PSP (Tables S3, S4, and S5). *KAT8*, the only therapeutic gene, showed significant associations with decreased expression in brain tissue and blood, as well as a significant association with increased methylation. Of the remaining nine genes, *IDUA*, *FMNL1*, *PRSS36*, and *TMEM175* had significant associations in mQTL sources. Additionally, *FMNL1*, *LRRC37A2*, and *PRSS36* had significant associations replicated in the multi-ancestry eQTL data (p_SMR_multi_ < 2.95 × 10^−6^; Table S7).

### Drug target discovery using significant genes identifies 41 novel gene targets for follow-up study

Using the approach previously outlined in our introduction and methods for drug target gene nomination, we categorized 159 gene hits into one of three tiers (Table 3). In our first tier, novel genes, we nominated 41 gene targets. SMR results for the novel genes are listed in Table S8. Genes are categorized as novel if they are in druggable regions of the genome that can be targeted by common molecular methods and currently have no FDA-approved treatment for any NDD as identified by current literature, knowledge base, and drug databases. Our second tier, known genes, had three gene targets identified—*MAPT*, *KCNN4* (MIM: 602754), and *ADORA2B* (MIM: 600446). Known genes are genes that have at least one FDA-approved therapeutic for treatment of an NDD (Table S9). The 3 nominated known gene targets are targeted by four therapeutics—apomorphine, carbidopa, istradefylline, and riluzole. Currently, these therapeutics are used for the treatment of PD symptoms (apomorphine, carbidopa, istradefylline) and prolonged survival for ALS (riluzole). In our last and largest tier, difficult genes, we identified 115 gene targets with no currently known therapeutics that target these genes and no known druggability. A total of 52 of the identified difficult genes exhibited at least two significant associations, with *LRRC37A2* having the maximum number of significant associations at 25 associations across AD and PD.

### Network analysis provides insight into druggable companion genes to non-druggable genes of interest

**Figure 2:**
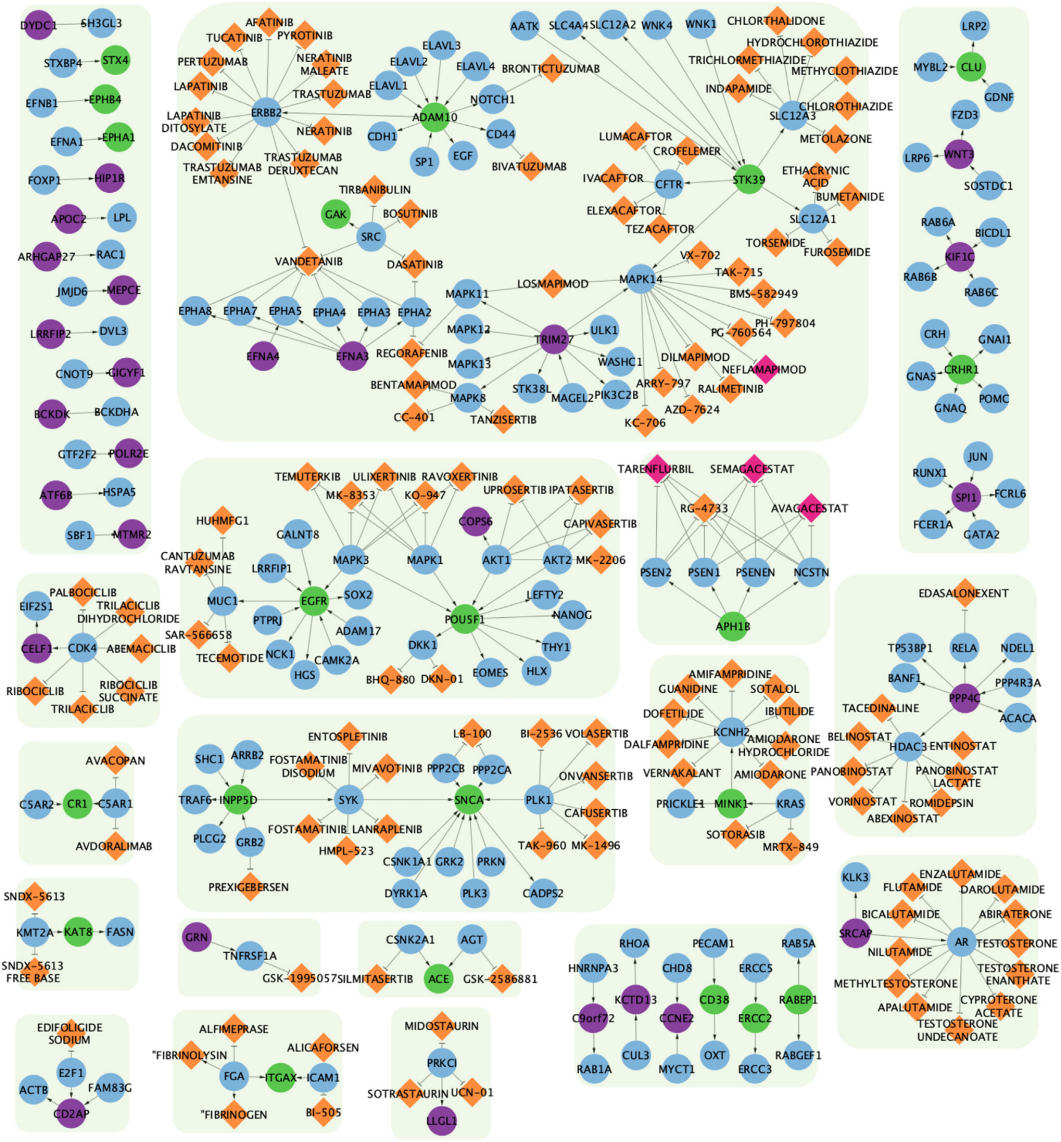
Network visualization of novel and difficult genes, companion genes, and drugs Graph network visualization of both novel (green nodes) and difficult (purple nodes) genes and their SIGNOR-curated partners (blue nodes). Drugs that interact with companion genes are denoted by orange-colored diamonds while FDA-approved drugs for use in NDD treatment are colored pink. Connecting arrows indicate the direction from regulator gene to target gene. Note: green boxes surrounding the gene-drug groupings serve as a visual guide to help separate non-connected groups.

We further implemented a gene network analysis for our novel and difficult tier candidates to identify potential proxy gene targets within each nominated genes’ SIGNOR curated network. In the novel gene tier, we identified 87 companion genes of which 58 are considered potentially druggable (Table S9). Of the 58 druggable companion genes, 30 were found to be targeted by a known drug, and a further five are targeted by therapeutics approved for treatment of AD. The five companion genes with AD-targeted therapeutics are *NCSTN* (MIM: 605254), *MAPK14* (MIM: 600289), *PSEN1* (MIM: 104311), *PSEN2* (MIM: 600759), and *PSENEN* (MIM: 607632), which are all targeted by tarenflurbil, semagacestat, and avagacestat. *MAPK14* is targeted by neflamapimod, an oral p38 alpha kinase inhibitor that the FDA approved for use in the treatment of AD and LBD (Figure 2). Further analysis of difficult gene co-expression networks identified 27 genes with 65 curated companion genes (Tables S10, S11, and S12; Figure S1). Of the 65 identified companion genes for the difficult target tier, 34 were found to be druggable with 18 having known drugs. *MAPK14* was the only companion gene to have a therapeutic approved (neflamapimod) to treat an NDD in the difficult target tier. *MAPK14* was identified as a companion gene to the difficult gene *TRIM27* (MIM: 602165) (Figure 2).

Drug toxicity analyses were conducted using liver eQTL data as a proxy. No novel gene was found to show significant SMR expression in liver eQTL data. However, using companion genes, we identified *OXT* (MIM: 167050), a companion to the novel gene *CD38* (MIM: 107270), to be significantly expressed in liver eQTL in the context of ALS. While *CD38* was found to be significant in AD and PD but not ALS, it is a consideration that should be considered as comorbidity may exist between ALS and AD or PD. *TOMM40* (MIM: 608061) is the only difficult gene to show significance in liver eQTL data. There are no companion genes to a difficult gene with significant expression in liver eQTL data.

### Multi-ancestry analyses reveal opposing gene expression patterns in significant disease risk loci between non-European and European ancestries

To investigate our findings in more diverse data, we also performed SMR with multi-ancestry eQTL data, aiming to replicate previous significant results (p_SMR_multi_ < 2.95 × 10^−6^ and p_HEIDI_ > 0.01) and nominate targets that show evidence toward generalizability across multiple ancestry groups. After multiple test corrections, we significantly replicated 11 total associations corresponding to 9 genes that were nominated in our initial analysis: *ARHGAP27*, *ARL17A*, *GPNMB* (MIM: 604368), *KANSL1*, *LRRC37A2*, *PILRA* (MIM: 605341), *PILRB* (MIM: 605342), *PRSS36*, and *ZNF232* (MIM: 616463) (see Table S7).

We then categorized these nine replicated genes based on their druggable status. One of the replicated hits, *GPNMB*, is classified in our novel gene tier, meaning that it is druggable and has no known treatment approved for treatment of NDDs, which suggests this gene may be an interesting but also potentially generalizable target across ancestries. The remaining eight replicated genes were in our difficult tier, meaning they are currently considered non-druggable through the small molecule modality.

### Nominated genes of interest found to be expressed in disease-relevant adult human brain snRNA-seq expression data

To provide additional evidence for biological relevance of our nominated targets, we investigated whether the 159 significant genes nominated through SMR (P_SMR_multi_ < 2.95 × 10^−6^ & P_HEIDI_ > 0.01) were expressed in relevant cell types from adult human brain snRNA-seq data.

**Figure 3:**
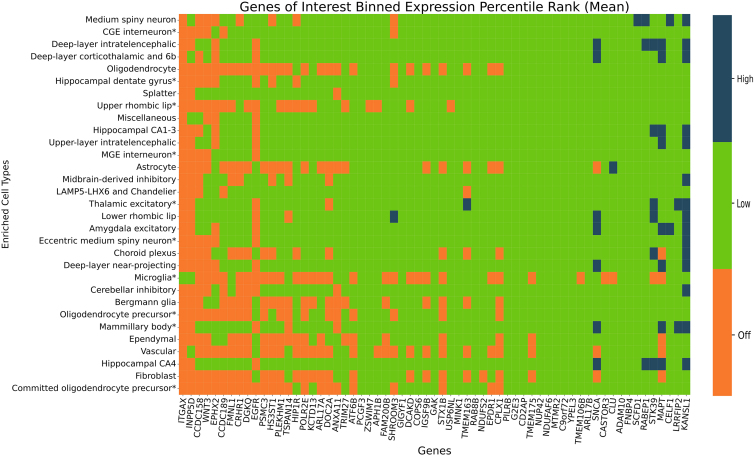
Single-cell RNA sequencing expression for significant genes (p_SMR_multi_ < 2.95 × 10^−6^ and p_HEIDI_ > 0.01) Expression of a given gene within each cell type is categorized as being highly expressed (dark blue, top 10% of all genes), intermediately expressed (green, middle 80%), or undetected (orange, bottom 10%). Genes that had off mean EPR values across all tested cell types were excluded from the heatmap. Cell types found to be enriched with AD and/or PD-relevant genes are marked by an asterisk. All other NDDs did not show significant cell type enrichment in any tissue, partially impacted by the much-smaller reference disease GWAS sizes.

We tested the 159 genes found to be significant against adult human snRNA-seq data to identify expression in varying brain single-cell types (P_SMR_multi_ < 2.95 × 10^−6^ & P_HEIDI_ > 0.01). We calculated the mean and median EPR for each gene across cells corresponding to each of the 31 tested cell types. To ease interpretation, we additionally binned the mean EPR values into three expression categories: off, low, and high based on the mean EPR value for each gene-cell-type combination. Using our binned mean EPR values, we found that 40 genes had all exclusively off EPR values while 16 genes had exclusively all low EPR values across all 31 tested cell types (Figure 3; Tables S13 and S14). We identified 11 genes (*KANSL1*, *LRRFIP2* [MIM: 614043], *CELF1* [MIM: 601074], *MAPT*, *STK39* [MIM: 607648], *RABEP1* [MIM: 603616], *SCFD1* [MIM: 618207], *CLU* [MIM: 185430], *SNCA* [MIM: 163890], *TMEM163* [MIM: 618978], and *SHROOM3* [MIM: 604570]) that had at least one gene highly expressed in any single cell type, with *KANSL1* having the most high EPR values across 15 cell types (Figure 3; Tables S13 and S14). Detailed breakdowns of both mean and median EPR values are provided in Figures S2–S4 and Tables S13 and S14. Cell types hippocampal CA4 and deep-layer intratelencephalic had the most genes with high EPR values, and hippocampal CA4 had the least number of genes with off EPR values (n_high_ = 5, n_low_ = 89, n_off_ = 65). The vascular cell type had the highest number of genes with off values (n_off_ = 123).

We also investigated the nominated genes in disease-relevant cell types—Bergmann glia, caudal ganglionic eminence (CGE) interneuron, committed oligodendrocyte precursor, deep-layer intratelencephalic, eccentric medium spiny neuron, hippocampal CA1–3, hippocampal dentate gyrus, LAMP5, LHX6 and chandelier, medial ganglionic eminence (MGE) interneuron, mammillary body, microglia, midbrain derived inhibitory, oligodendrocyte precursor, thalamic excitatory, upper layer intratelencephalic, and upper rhombic lip—identified in Alvarado et al. to be significantly enriched with AD- and PD-relevant genes.^7^ Significant genes from our SMR analysis were highly expressed in three of these disease-relevant cell types: thalamic excitatory, eccentric medium spiny neuron, and mamillary body. *KANSL1* had high expression in all three listed cell types (Figure 3). Of the 159 tested genes, 20 had all low EPR values in the disease-relevant cell types. The thalamic excitatory cell type had the least number of off EPR values for genes *INPP5D* (MIM: 601582), *ITGAX* (MIM: 151510), *EGFR* (MIM: 131550), and *DOC2A* (MIM: 604567).

## Discussion

As the global population continues to age, the threat posed by NDDs presents an overwhelming and multifaceted challenge. Our research aims to address the challenge of treating NDDs by identifying therapeutic targets anchored in genetic data—a proven strategy in therapeutic development. Our conservative approach primarily focuses on small-molecule drug targets given the breadth of available data as this class of therapeutics has the most studied and reliable gene-based annotations available. Implementation of this strategy has been impeded by the small sample sizes and the dispersed nature of genetic- and disease-related data, such as proteomics and transcriptomics. Here, we attempted to address this need by creating and implementing an open-source framework to identify druggable targets across varied NDDs.—→

In our targeted analyses, we were unable to identify any potentially functionally relevant genes that were significant across all six tested NDDs. While NDDs share prominent hallmarks, such as cell death, inflammation, and pathological protein aggregation, the role that each hallmark and its associated biological processes play in the pathogenesis of each NDD differs, creating a spectrum.^2^^,^^34^ We identified *MAPT*, *CRHR1*, *KANSL1*, *ARL17A*, and *ARHGAP27* to be independently significant in multiple different omics for AD, PD, and PSP (Tables S3, S4, and S5). *MAPT* was found to have significant associations with primarily increased expression for AD, PD, and PSP across eQTL and mQTL omic data, as supported by previous research.^35^^,^^36^^,^^37^ The *MAPT* locus, 17q21, contains genes *CRHR1*, *KANSL1*, *ARL17A*, and *ARHGAP27*, and mutations in this locus have been previously associated with both PD and PSP.^38^ Due to the complexity and observed linkage disequilibrium found in the 17q21 locus and the limitations of the SMR framework, causality cannot be established or inferred without further functional follow up. Previous evidence of significant association of this locus in AD is more fragmented and sparser. The 17q21 locus, which includes genes *KANSL1* and *MAPT*, has been previously implicated in AD.^39^ *ARL17A* has been reported to harbor eQTL SNPs implicated in both brain and blood tissues in relation to AD.^40^ *CRHR1*’s role in stress response has been hypothesized to exacerbate AD pathologies given its abundance in the brain, including areas implicated in learning and memory.^41^ Lastly, evidence of *ARHGAP27*’s significance in AD includes associations between complex traits such as cognitive functioning, reaction time, and cortical structure phenotypes.^42^^,^^43^

A deep dive into *KANSL1* highlights its role in autophagy pathways. *KANSL1* is a core member of the non-specific lethal (NSL) complex that binds to *MOF* (also known as *KAT8*), which is necessary for the acetylation of histone H4 lysine 16 acetylation (H4K16ac).^44^^,^^45^ Some studies have associated elevated expression levels of *KANSL1* with over-promoted autophagic activity, resulting in cell death and cytotoxicity from autophagosome accumulation; however, further research is required to understand this mechanism.^46^ Additional research into the role of autophagy and lysosomal pathways in NDDs have indicated that altered autophagy function results in the inability to clear out protein aggregates, resulting in cell death and potentially contributing to disease pathogenesis and neurodegeneration.^45^^,^^47^^,^^48^^,^^49^ Our results are consistent with previous research, linking increased expression of *KANSL1* with neurodegenerative effects. When assessing associations with AD, PD, and PSP, *KANSL1* is associated with an increased expression in brain mQTLs, three different brain eQTLs (psychEncode, multi-ancestry, and anterior cingulate cortex), and spinal cord eQTLs. The consistent significance of *KANSL1* and most of our gene hits in mQTL omics highlights the influence of DNA methylation for NDD pathogenesis and progression.

We identified 10 genes as significant in two diseases. The nominated genes do not share any explicit relationships but are common in their importance for varying biological processes and cellular functions, such as cell proliferation and differentiation, degradation of transmembrane proteins, calcium homeostasis, and autophagy regulation.^50^^,^^51^^,^^52^^,^^53^^,^^54^ Six of our nominated genes, *ARL17B*, *KAT8*, *LRRC37A2*, *PRSS36*, *SPPL2C*, and *WNT3*, are associated with both AD and PD. Given the significantly larger sample sizes and increased power of the two diseases in GWAS summary statistics, we did not find this unexpected. LBD and PD share two genes, *IDUA* and *TMEM175*, while AD and PSP share *FMNL1*, and PD and PSP share *PLEKHM1* (Tables S3, S4, and S6). In general, the bulk of the gene hits were found to be significant in mQTL data for both brain and blood tissues (n_whole brain_ = 4; n_whole blood_ = 4) followed by cortex eQTLs (n_cortex metaBrain_ = 6, n_cortex GTEx_ = 3, n_Frontal Cortex BA9_ = 2, n_prefrontal cortex_ = 3).

The only gene found in two diseases, AD and PD, that could be targeted therapeutically was *KAT8*, which we previously mentioned in the context of the *KANSL1* gene. In literature, *KAT8* (lysine acetyltransferase 8) is identified as a protein-coding gene that plays a vital role in the NSL complex for acetylation of H4K16ac.^49^ Scientific observation has identified the consequences of autophagic dysfunction in NDDs to include impaired neuronal function, neuronal death, and neuron loss. In opposition to the expression pattern of *KANSL1*, decreased expression of *KAT8* is associated with deacetylation of H4K16ac in AD patients, while an overexpression of *KAT8* has been linked to increased expression levels of neuroprotective soluble amyloid precursor protein (sAPP)α and β-secretase (BACE)2 and decreased levels of sAPPβ and *BACE1* (MIM: 604252).^55^ In our results, we found blood mQTLs for AD and brain mQTLs for AD and PD to be associated with increased expression of *KAT8*; this is in contrast to gene expression in some of the same tissues, such as blood and brain mQTLs, for *KANSL1*. The associated increased expression of *KAT8* in our results suggests that an increase in expression may be correlated with excess autophagy resulting in cell death, which is a hallmark symptom of all three NDDs (AD, PD, and LBD).^56^^,^^57^ There are currently no FDA-approved therapeutic that target *KAT8* in NDDs. However, compound MG149, a histone acetyltransferase inhibitor, has been found to reduce proinflammatory genes via inhibition of MYST (named for protein members MOZ, Ybf2/Sas3, Sas2, and Tip60)-type histone acetyltransferase *KAT8*.^58^ MG149 has also been found to be effective in restoring impaired autophagic flux via the inhibition of histone acetylation of H4K16ac in cases of ischemic stroke and inflammatory diseases.^48^^,^^59^ Further research into the application of MG149 could result in a novel treatment targeting the characteristic accumulation of toxic proteins in NDDs.

FTLD was the only tested disease that did not have any suggestive targets at our test correction threshold. This may be because the FTLD GWASs had the smallest sample size out of all the diseases tested, and results will likely improve as larger FTLD GWASs are conducted. As there were no significant results for FTLD after correction, we decided to investigate potential pleiotropic relationships between FTLD and the other NDDs. To do this, we looked for FTLD associations at a less-stringent p value threshold (p_SMR_multi_ < 0.05) only in the 254 unique candidate genes passing our original threshold of p_SMR_multi_ < 2.95 × 10^−6^, a process detailed by Baird et al.^60^ This resulted in 124 FTLD hits made up of 31 unique genes that have a potential pleiotropic relationship between FTLD and another NDD. Of those 31, 12 were classified as druggable through our sources (*STX4* [MIM: 186591], *STX1B* [MIM: 601485], *VKORC1* [MIM: 608547], *POU5F1* [MIM: 164177], *HSD3B7* [MIM: 607764], *PSORS1C1* [MIM: 613525], *SLC44A4* [MIM: 606107], *CD38*, *EPHX2* [MIM: 132811], *FBXL19* [MIM: 609085], *CLU*, and *CDSN* [MIM: 602593]). All 12 fall into the novel tier of drug targets, representing potential avenues for drug repurposing for FTLD.

Our creation of a drug target classification scheme is an attempt to inform drug discovery and repurposing from genes considered significant with evidence of causative roles in NDDs. Further inspection of our 41 novel genes provides multiple insights into the genes that compose the tier. Many genes that compose our novel tier have therapeutics used in the treatment of multiple types of cancers and tumors. Fourteen of our novel genes have therapeutics approved for use in the treatment of cancer (MONDO_0004992). Other commonly approved indications for therapeutics that target our novel genes include, but are not limited to, neoplasm (EFO_0000616), hypertension (EFO_0000537 [MIM: 145500]), and cardiovascular disease (EFO_0000319). *GPNMB*, which is of particular interest due to support for its role in PD*,* falls into this grouping of 14 genes. Similar to its role in cancer and tumor growth, our results highlight *GPNMB*’s pattern of increased expression as shown in brain-related PD eQTLs. We were able to find replication of increased GPNMB expression in brain-related tissues in Li et al., Ortiz et al., and Nalls et al.^61^^,^^62^^,^^63^ Glembatumumab vedotin is one of the therapeutics that targets *GPNMB* where its primary mechanism of action (MOA) is tubulin inhibition.^25^ Consequently, glembatumumab vedotin’s inhibitory MOA could be repurposed for use in PD treatment for suppression of inflammation given the recognized role of inflammatory response/neuroinflammation in PD onset and progression.^64^^,^^65^ However, any treatment developed targeting *GPNMB* would most likely be limited in treating people of European ancestries due to the gene’s importance and role compared to non-European ancestries—further increasing inequality.

Our largest and most uncertain classification tier contains 121 difficult genes. Despite not having any currently known therapeutics, this classification tier could lead to the development of NDD-targeted therapeutics or the repurposing of existing ones. Our approach for these genes focused on analyzing well curated networks centered on each difficult gene to identify any partner genes with existing therapeutic drugs. This approach provides us context into any biological pathways and processes that may be affected by a targeted treatment, which could help eliminate the time and resources spent on developing and researching ineffective therapies.

The smallest tier, known genes, is composed of the three genes targeted by NDD-targeted therapeutics. Apomorphine, carbidopa, and istradefylline are indicated for use in treatment of PD. Riluzole is indicated for the treatment of ALS but has undergone phase 2 clinical trials for use in treatment of AD. The results in clinical trials for use of riluzole in AD treatment were promising with cerebral glucose metabolism, an AD biomarker, preserved in patients receiving riluzole compared to those in the placebo group.^66^ The researchers conducting the study suggested a more powerful and longer study, but no follow up studies have yet been initiated. Our results support the continued follow up of riluzole clinical trials.

Focusing on genes we flagged as putatively associated with risk across multiple diseases, 13 of 15 were noted as being at least moderately expressed in cell types of interest (those with enriched expression for GWAS risk signatures) from single-nucleus sequencing. Positive beta coefficients at these genes from the SMR analysis suggest that if an expression effect was inhibited, it could be possible to reduce disease risk. Two of these genes, *KANSL1* and *MAPT*, showed significant positive associations (defined as a gene with positive beta values in more than 50% of its significant SMR associations) between risk and expression in our SMR analyses, providing a contextual insight for future follow up.

Genes such as *GPNMB* had different expression patterns in European and non-European ancestries. For example, *GPNMB* had decreased associated expression in multi-ancestry eQTLs but an increased associated expression in all other tested eQTLs. Previous research in certain Asian populations has found no significant association between *GPNMB* and PD.^67^^,^^68^ Rizig and colleagues, conducting the largest PD GWAS in the African and African admixed populations in ∼200,000 individuals, of which 1,488 are cases, report the following per SNP in *GPNMB*: rs858275, p = 0.1250, beta = −0.0824, indicating no association in African/African admixed ancestries. Our multi-ancestry data report the same direction of expression in GPNMB SNP rs858275, p = 1.080397 × 10^−8^, beta = −0.107745 in PD. Interestingly, the reported direction of expression in our multi-ancestry data and Rizig and colleagues’ data contrasts with the direction of expression reported for European ancestries in addition to indicating no significant associations (Table S13).^69^

The limitations we encountered in our research included limited GWAS data for diseases, excluding AD and PD, limited non-eQTL omic data, limited multi ancestry omic data, and reference panels, as well as non-small-molecule drug target annotations. In general, the availability of public and free omic and drug target data is increasing. As new data are published, we intend to conduct updates and incorporate new omic types into our analysis, such as more pQTL, single-cell QTLs, and splicing QTLs (sQTLs). This was a hypothesis generating effort at scale, and while there are too many results for us to follow up in detail ourselves, we hope that the single-nucleus enrichments will help guide others to the correct cell types for their studies and further target nomination efforts. The incorporation of additional multi-omic data should provide new and novel insights into the complex underpinnings of NDDs, while incorporating additional data on drug target modalities, such as monoclonal antibodies and gene therapies, will open new treatment possibilities.

The limitation we feel that presents the most barriers is limited multi-ancestry data. The state of diversity in the NDD research space has historically been Eurocentric, which remains the case in this study due to the limited availability of omic data from non-European participants. One of the distinguishing aspects of this study is the inclusion of multi-ancestry eQTL data in the search for generalizable drug targets. This is particularly important in an era where precision medicine and machine learning can introduce inherent bias when using reference data from solely European populations. We identified common hits, which were consistent with current understanding that there are NDD risk loci that are shared across genetic ancestries while providing insight on which gene loci and differences in expression may play a role in NDD development and treatment in non-Europeans. It is worth noting that while replication was limited at our stringent significance threshold, we were able to make some interesting observations. While we made attempts to include a limited set of multi ancestry data in the future, we would like to be able to include more multi-ancestry disease GWASs and omic data to make more meaningful insights. We recognize that we will need more multi-ancestry QTL and GWAS data for these results to be truly generalizable across different populations. We look forward to the increasing availability of non-European data with the growth of data sources such All of Us, an NIH research program focusing on inclusion of health data of marginalized populations in the United States.^70^

This report is a description of the foundation for a community-driven resource to identify and investigate future genetically derived drug targets in an open-source context. Ultimately, we are working on creating a network tool that incorporates multi-omic data, disease GWAS summary statistics, drug data, and other relevant data types to ease research such as this study, eliminating barriers to drug discovery and drug repurposing and potentially enabling precision medicine in the NDD space. Using multi-omics integration methods, deep learning techniques, and most importantly, community input to better parse and interpret the data presented by the platform, we aim to make our community resource a robust tool for NDD research.

## Data and code availability

This paper analyzes existing, publicly available data. All original code has been deposited at GitHub, which can be found on the Center for Alzheimer’s and Dementia GitHub (https://github.com/NIH-CARD/NDD_SMR) and Zenodo: https://doi.org/10.5281/zenodo.8425910. Results of SMR analyses can be browsed and downloaded from the Streamlit application (https://nih-card-ndd-smr-home-syboky.streamlit.app/) and csv versions are located at (https://drive.google.com/drive/folders/16lB70BgRKA8yjXuAdW3OntHIrR8gqADO).

## Web resources

Biomart python package, https://github.com/sebriois/biomart
GWAS catalog, https://www.ebi.ac.uk/gwas/
metaBrain, https://www.metabrain.nl/
Open Targets Platform, https://platform.opentargets.org/
Pyensembl, https://github.com/openvax/pyensembl
SIGnaling Network Open Resource (SIGNOR), https://signor.uniroma2.it/
Streamlit, https://nih-card-ndd-smr-home-syboky.streamlit.app/
Yang Lab, https://yanglab.westlake.edu.cn/software/smr/#Overview
Yang Lab, Data Resources, https://yanglab.westlake.edu.cn/software/smr/#DataResource

## Supplemental information

Tables S4, S5, S7, S8, and S11–S14

Document S2. Article plus supplemental information
