# omicSynth: An open multi-omic community resource for identifying druggable targets across neurodegenerative diseases

**DOI:** 10.1016/j.ajhg.2025.08.002

**Published:** 2025-08-12

**Authors:** Chelsea X. Alvarado, Mary B. Makarious, Cory A. Weller, Dan Vitale, Mathew J. Koretsky, Sara Bandres-Ciga, Hirotaka Iwaki, Kristin Levine, Andrew Singleton, Faraz Faghri, Mike A. Nalls, Hampton L. Leonard

## Main text

(The American Journal of Human Genetics *111*, 150–164; January 4, 2024)

H.L.L. should have been added as having contributed to data collection, analysis, and the drafting of the manuscript and preparation for publication. Additionally, in the discussion where it is noted that “Our largest and most uncertain classification tier contains 121 difficult genes…,” the number 121 should be corrected to 115. The authors regret the oversights.

