# Supplementary material for: omicSynth: An open multi-omic community resource for identifying druggable targets across neurodegenerative diseases
Source: Am J Hum Genet. 2024 Jan 4;111(1):150–64. doi: 10.1016/j.ajhg.2023.12.006 (PMC10806756; doi:10.1016/j.ajhg.2023.12.006)
Supplement: Document S1. Figures S1–S4, Tables S1–S3, S6, S9, S10, and S15 [file mmc1.pdf]

The American Journal of Human Genetics, Volume *111*

Supplemental information

omicSynth: An open multi-omic community resource for identifying druggable targets across neurodegenerative diseases

Chelsea X. Alvarado, Mary B. Makarious, Cory A. Weller, Dan Vitale, Mathew J. Koretsky, Sara Bandres-Ciga, Hirotaka Iwaki, Kristin Levine, Andrew Singleton, Faraz Faghri, Mike A. Nalls, and Hampton L. Leonard

Supplementary Tables Legend

Table S1: Summary of SMR data mining across diseases. This table provides a summary of the number of unique genes, genes found to be significant in liver eQTL tissue, eQTL specific genes, number of genes replicated in multi ancestry eQTL data, total number of gene found to be therapeutic/druggable, the percentage of therapeutic genes, total number of non-therapeutic/non-druggable genes, and the percentage of non-therapeutic genes for each diseases. This data is provided at 4 levels, an overall summary of all tested genes and three significance thresholds: $p_{SMR\_multi} < 0.05$ & $p_{HEIDI} > 0.01$, $p_{SMR\_multi} < 2.95 \times 10^{-6}$ (testing all protein coding genes) & $p_{HEIDI} > 0.01$, and $p_{SMR\_multi} < 1.58 \times 10^{-8}$ (testing all protein coding genes across all omics) & $p_{HEIDI} > 0.01$.

Table S2:All unfiltered SMR association summary statistics. Table provides a link to data files for download.

Table S3: Extended data for candidate genes for multiple neurodegenerative diseases (Table 1). This table provides extended data for genes with functional inferences passing multiple test correction at a multi-SNP SMR $P < 2.95 \times 10^{-6}$ for multiple neurodegenerative diseases. We provide details for the most significant *-omic association detected as well as the most significant single SNP in that set of gene level associations. Additionally, we provide all the omics and diseases in which a given gene has significant associations.

Table S4: Summary statistics for all significant gene associations. This table provides summary statistics and SMR results for candidate genes with associations passing multiple test correction at a multi-SNP SMR P < 2.95E-06. Columns ending with GWAS and QTL provide values for the tested probe from the source QTL and GWAS summary statistics used in the SMR analyses.

Table S5: Extended Summaries of candidate genes in three neurodegenerative diseases. This table provides extended data on candidate genes passing multiple test correction at a multi-SNP SMRP < 2.95E-06 for three neurodegenerative diseases. We provide summary statistics for each significant association.

Table S6: Extended Summaries of candidate genes in two neurodegenerative diseases. This table provides extended data on candidate genes passing multiple test correction at a multi-SNP SMRP < 2.95E-06 for two neurodegenerative diseases. We provide summary statistics for each significant association.

Table S7: Summary statistics for candidate genes replicated in multi ancestry data. This table provides summary statistics for all replicated candidate genes' significant associations across all *-omics.

Table S8: Summary statistics for all novel class genes. This table provides summary statistics of candidate genes classified as *novel,* meaning the gene is considered druggable but does not have NDD specific therapeutics that target the gene. Significant associations for each gene are provided.

Table S9: Summary statistics for all known class genes. This table provides summary statistics of candidate genes classified as *known,* meaning that the gene has therapeutics that are approved for use in at least one NDD. We provide all significant associations for each *known* gene.

Table S10: Summaries of the novel target class of genes including network members. This table lists all candidate genes that fall into the novel tier in our druggable gene classification scheme. For each gene we provide a summary of how many omics a gene has significant associations in and in which diseases. We identify companion genes for each gene using the Signor database and in bold are genes that have been found to have significant associations in our SMR results (multi-SNP SMRP < 2.95E-06).

Table S11: Summary statistics for all difficult class genes. This table provides summary statistics of candidate genes classified as *difficult,* meaning the gene is not currently considered druggable and no therapeutics target the gene.

Table S12: Summaries of the difficult target class of genes including network members. This table lists all candidate genes that fall into the difficult tier in our druggable gene classification scheme. For each gene we provide a summary of how many omics a gene has significant associations in and in which diseases. We identify companion genes for each gene using the Signor database and in bold are genes that have been found to have significant associations in our SMR results (multi-SNP SMRP < 2.95E-06). We provide additional information regarding potential liver toxicity issues by cross referencing companion genes against significant associations in the liver omic.

Table S13: Mean expression percentile ranks in single cell data for multi-NDD gene targets. This table provides calculated mean expression rank values using snRNA-seq data from Silleti et al. (2022). Mean values for each gene-cell type were obtained by using scRNA-seq values for all cell type specific individual cells for a specific gene.

Table S14: Median expression percentile ranks in single cell data for multi-NDD gene targets. This table provides calculated median expression rank values using snRNA-seq data from Silleti et al. (2022). Median values for each gene-cell type were obtained by using scRNA-seq values for all cell type specific individual cells for a specific gene.

Table S15: Comparison of GPNMB across multiple genetic ancestries. This table provides basic summary statistics of our GPNMB significant associations in comparison to other GPNMB summary statistics. We summarize data in African American/African admixed, Chinese, and Taiwanese genetic ancestries to confirm that our multi ancestry association direction was consistent with previous findings in other non-European ancestries.

Supplementary Figure(s)

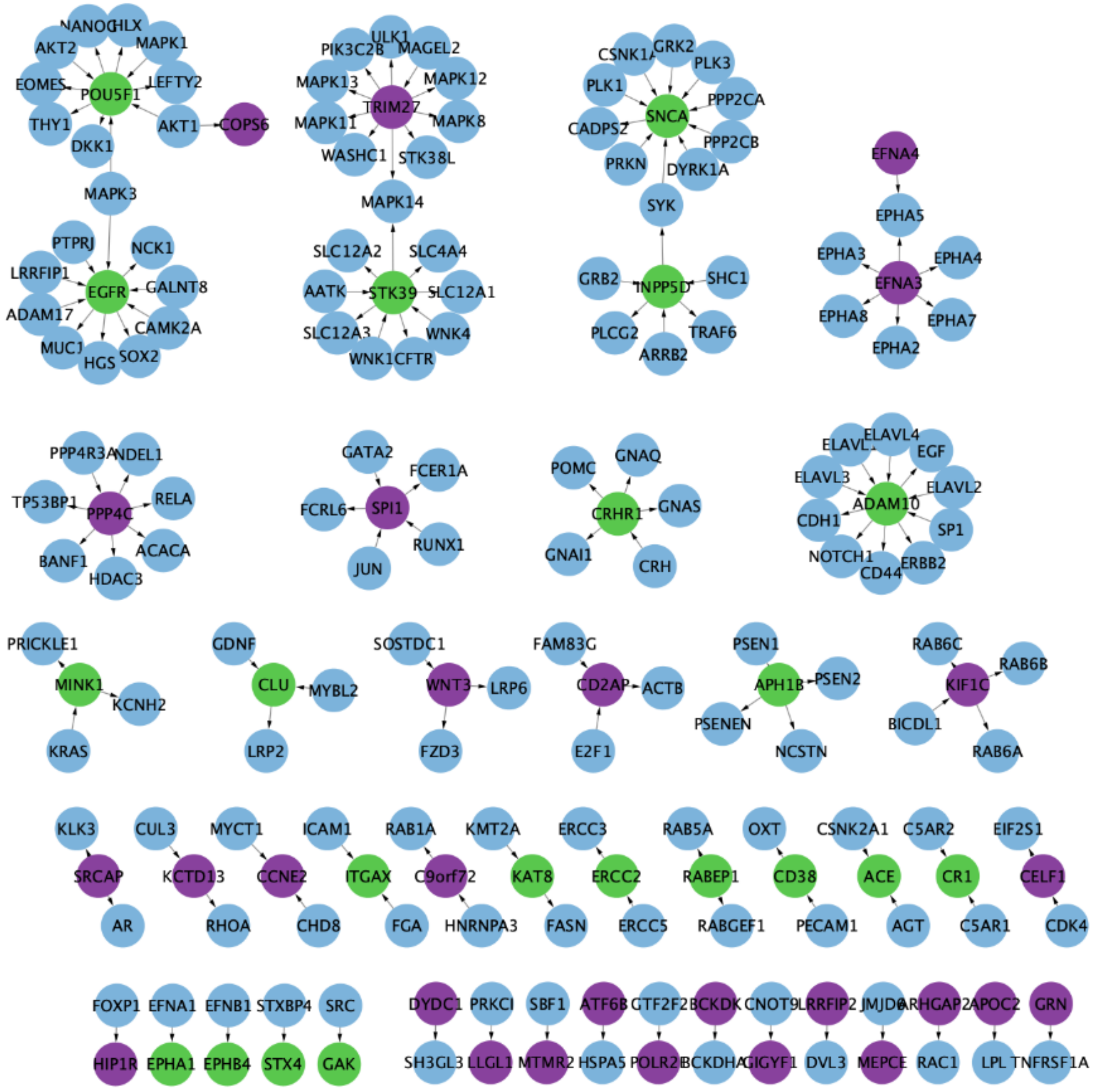

Figure S1: Network Visualization of *Novel* and *Difficult* genes companion genes Graph network visualization of both *novel* (green nodes) and *difficult* (purple nodes) genes and their SIGNOR curated partners (blue nodes). The direction of connecting arrows indicates interaction from regulator to target.

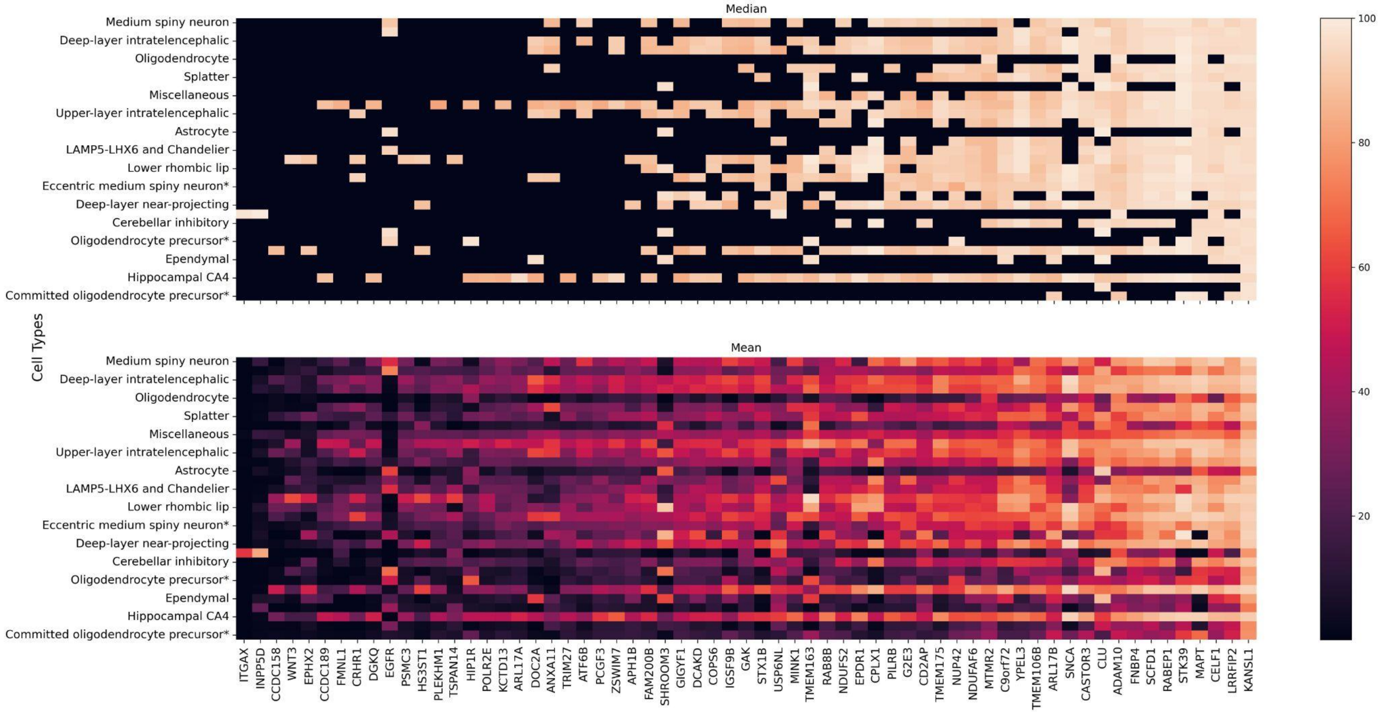

Figure S2: scRNA-seq expression for significant genes (p_{SMR_multi} < 2.95×10-6 and p_{HEIDI} > 0.01). Heatmaps were used to illustrate both the median (top plot) and mean (bottom plot) expression percentile rank for each gene-celltype combination using significant genes and disease relevant cell types.

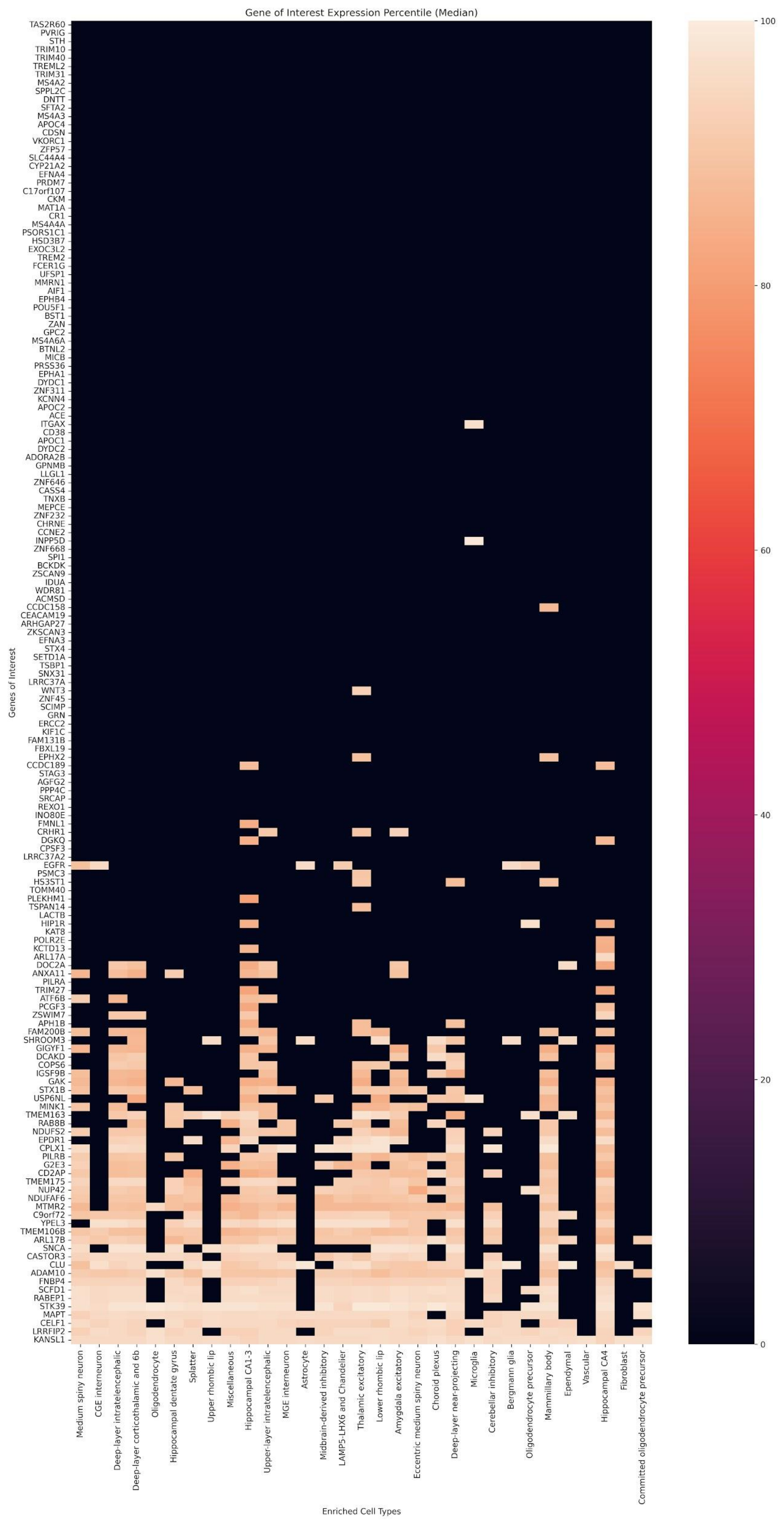

Figure S3: Median scRNA-seq expression for all significant genes. Heatmap illustration of the median expression percentile rank value for each gene-celltype combination.

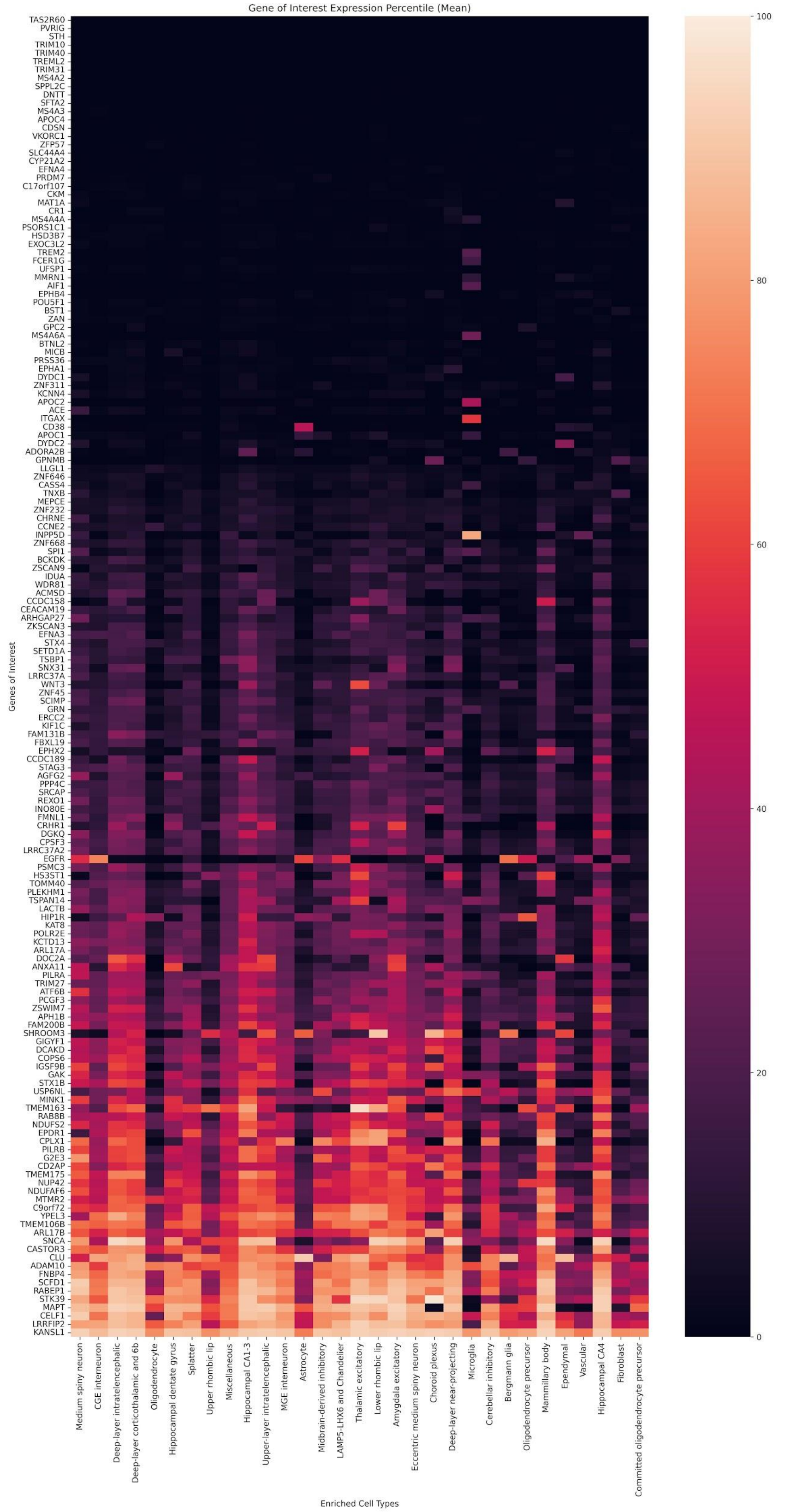

Figure S4: Mean scRNA-seq expression for all significant genes. Heatmap illustration of the mean expression percentile rank value for each gene-celltype combination.

Disease	Total Genes (Unique)	Liver Genes	Total eQTL Genes (non multi ancestry)	Replicated in Multi Ancestry	Total Therapeutic Genes	% Therapeutic	Total Non-therapeutic Genes	% Non-therapeutic
**All Tested Genes (Protein Coding)**								
AD	16833	1597	15112	8404	3,562	21.2%	13,271	78.8%
ALS	16875	1610	15163	8408	3,565	21.1%	13,310	78.9%
FTLD	16788	1537	15038	8394	3,551	21.2%	13,237	78.8%
LBD	16797	1540	15069	8388	3,554	21.2%	13,243	78.8%
PD	16872	1596	15159	8407	3,566	21.1%	13,306	78.9%
PSP	16042	1033	13839	8073	3,420	21.3%	12,622	78.7%
**Significance p_SMR_multi < 0.05 & p_HEIDI > 0.01**								
**Disease**	**Total Significant Genes (Unique)**		**Total eQTL Genes (non multi ancestry)**	**Replicated in Multi Ancestry**		**% Therapeutic + Sig**		**% Non-therapeutic + Sig**
AD	8	175	3189	2079	1,142	14275.0%	3,806	47575.0%
ALS	3188	83	1857	1260	715	22.4%	2,473	77.6%
FTLD	2318	78	1243	810	542	23.4%	1,776	76.6%
LBD	2530	82	1384	900	580	22.9%	1,950	77.1%
PD	3592	108	2161	1434	811	22.6%	2,781	77.4%
PSP	2275	30	1270	842	574	25.2%	1,701	74.8%
**Significance p_SMR_multi < 2.95E-06 (testing all protein coding genes) & p_HEIDI > 0.01**								
AD	116	2	68	7	31	26.7%	85	73.3%
ALS	3	0	3	0	0	0.0%	3	100.0%
FTLD	0	0	0	0	0	0.0%	0	0.0%
LBD	5	0	1	0	1	20.0%	4	80.0%
PD	46	3	33	5	15	32.6%	31	67.4%
PSP	9	0	5	3	2	22.2%	7	77.8%
**Significance p_SMR_multi < 1.58E-08 (testing all protein coding genes across all omics) & p_HEIDI > 0.01**								
AD	47	1	19	19	14	29.8%	33	70.2%
ALS	1	0	0	0	0	0.0%	1	100.0%
FTLD	0	0	0	0	0	0.0%	0	0.0%
LBD	2	0	0	0	1	50.0%	1	50.0%
PD	24	1	14	14	8	33.3%	16	66.7%
PSP	8	0	5	5	2	25.0%	6	75.0%

All protein coding associations can be download from this link: https://drive.google.com/file/d/1l70F2UcEoKEFJYRM4LYtbsr61QP2VjAV/view?usp=share_link (~200mb)

Gene	Omic	Disease	topRSID	beta, SMR	SE, SMR	p, SMR	p, multi-SNP SMR	p, HEIDI	Diseases	Omics
									AD	Cerebellum eQTL, Cortex eQTL
ARL17B	Spinal cord eQTL	PD	rs199451	-0.202836	0.0332609	1.07E-09	6.42E-09	0.01223556	PD	Spinalcord eQTL
									PD	Spinalcord eQTL
KAT8	Tibial Nerve eQTL	PD	rs9936329	-0.428193	0.0892292	1.60E-06	3.76E-08	0.06971106	AD	Cerebellum eQTL, Whole Brain meta-analysis mQTL, Cerebellar Hemisphere eQTL, Cortex eQTL, Tibial Nerve eQTL, Skeletal Muscle eQTL, Hypothalamus eQTL, Whole Brain eQTL, Cerebellum eQTL
									PD	Hippocampus eQTL, Cortex eQTL, Frontal Cortex BA9 eQTL, Prefrontal Cortex eQTL, Caudate Basal Ganglia eQTL, Skeletal Muscle eQTL, Hippocampus eQTL, Multi Ancestry Whole Brain Meta-analysis eQTL, Hypothalamus eQTL, Liver eQTL, Anterior Cingulate Cortex BA24 eQTL, Putamen Basal Ganglia eQTL, Amygdala eQTL, Whole Brain eQTL, Cerebellum eQTL, Nucleus Accumbens Basal Ganglia
LRRC37A2	Multi Ancestry Whole Brain Meta-analysis eQTL	PD	rs2532329	-0.22263	0.0255896	3.32E-18	1.22E-15	0.4841153	AD	Basal Ganglia eQTL, Spinalcord eQTL, Frontal Cortex BA9 eQTL, Hippocampus eQTL, Multi Ancestry Whole Brain Meta-analysis eQTL, Substantia nigra eQTL, Liver eQTL, Putamen Basal Ganglia eQTL, Amygdala eQTL
									PD	Whole Brain meta-analysis mQTL, Whole Blood mQTL, Cortex eQTL, Multi Ancestry Whole Brain Meta-analysis eQTL
									PSP	Spinalcord eQTL, Whole Brain meta-analysis mQTL, Whole Blood mQTL
KANSL1	Whole Brain meta-analysis mQTL	PD	rs1966345	0.15927	0.0174497	7.02E-20	2.28E-19	0.06522781	AD	Spinalcord eQTL, Whole Blood mQTL, Anterior Cingulate Cortex BA24 eQTL
									PSP	Multi Ancestry Whole Brain Meta-analysis eQTL, Hypothalamus eQTL
									PD	Cerebellar Hemisphere eQTL, Cortex eQTL, Caudate Basal Ganglia eQTL, Hippocampus eQTL, Multi Ancestry Whole Brain Meta-analysis eQTL, Hypothalamus eQTL, Anterior Cingulate Cortex BA24 eQTL, Putamen Basal Ganglia eQTL, Cerebellum eQTL, Nucleus Accumbens Basal Ganglia
ARL17A	Multi Ancestry Whole Brain Meta-analysis eQTL	PD	rs58879558	-0.337169	0.0385816	2.35E-18	3.03E-15	0.1614406	AD	Spinalcord eQTL, Amygdala eQTL
									AD	Whole Brain meta-analysis mQTL, Cerebellar Hemisphere eQTL, Cortex eQTL, Multi Ancestry Whole Brain Meta-analysis eQTL, Whole Brain eQTL
PRSS36	Whole Brain meta-analysis mQTL	AD	rs55667375	0.0928108	0.0167849	3.21E-08	6.46E-07	0.01582905	PD	Whole Brain meta-analysis mQTL
									AD	Whole Brain meta-analysis mQTL
									PD	Whole Brain meta-analysis mQTL, Whole Blood mQTL
MAPT	Whole Brain meta-analysis mQTL	PSP	rs1981997	1.14017	0.0815642	2.10E-44	2.10E-44	0.01425494	PSP	Whole Brain meta-analysis mQTL, Whole Blood mQTL
									LBD	
IDUA	Whole Brain meta-analysis mQTL	PD	rs6599388	-0.134337	0.0147854	1.03E-19	1.87E-12	0.01914885	PD	Whole Brain meta-analysis mQTL
									PD	
TMEM175	Whole Blood mQTL	PD	rs11248057	0.495391	0.0721195	6.46E-12	4.33E-11	0.6479261	LBD	Whole Blood mQTL
									PD	Whole Blood mQTL, Multi Ancestry Whole Brain Meta-analysis eQTL
									AD	Whole Brain meta-analysis mQTL, Whole Blood mQTL, Caudate Basal Ganglia eQTL, Nucleus Accumbens Basal Ganglia
ARHGAP27	Whole Blood mQTL	PSP	rs11012	4.15654	0.543286	2.00E-14	1.36E-13	0.07969932	PSP	Whole Blood mQTL, Whole Blood eQTL eQTLgen
									PD	Whole Blood mQTL
									PSP	Whole Brain meta-analysis mQTL, Whole Blood mQTL, Cortex eQTL, Skeletal Muscle eQTL
CRHR1	Whole Brain meta-analysis mQTL	PSP	rs12373139	1.79071	0.188188	1.81E-21	5.15E-18	0.2191273	AD	Whole Brain meta-analysis mQTL, Whole Blood mQTL
									PSP	Multi Ancestry Whole Brain Meta-analysis eQTL
FMNL1	Whole Blood mQTL	PSP	rs17630064	0.247221	0.0717373	0.0005685207	7.24E-08	0.06396753	AD	Whole Blood mQTL
									PD	Cortex eQTL, Frontal Cortex BA9 eQTL, Prefrontal Cortex eQTL, Cortex eQTL, Caudate Basal Ganglia eQTL, Skeletal Muscle eQTL, Anterior Cingulate Cortex BA24 eQTL, Putamen Basal Ganglia eQTL, Whole Brain eQTL
PLEKHM1	Skeletal Muscle eQTL	PD	rs12947718	-0.667265	0.0974424	7.50E-12	7.76E-12	0.03535971	PSP	Anterior Cingulate Cortex BA24 eQTL
									PD	Cortex eQTL metaBrain, Skeletal Muscle eQTL
WNT3	Cortex eQTL metaBrain	PD	rs9904865	-0.062473	0.0240469	0.009377936	1.37E-08	0.09647706	AD	Tibial Nerve eQTL
									PD	Cerebellum eQTL
SPPL2C	Prefrontal Cortex eQTL	PD	rs17577369	1.04066	0.182771	1.24E-08	1.30E-07	0.01842581	AD	Prefrontal Cortex eQTL

Gene	Disease	Omic	probeID	ProbeChr	Probe_bp	topRSID	topSNP_chr	topSNP_bp	A1	A2	beta, SMR	SE, SMR	p, SMR	p, multi-SNP SMR	p, HEIDI
		Cerebellum eQTL	ENSG00000228696	17	46361797	rs7225002	17	44189067	A	G	-0.0626033	0.0105364	2.82E-09	4.20E-07	0.5937533
	AD	Cortex eQTL metaBrain	ENSG00000228696	17	46361797	rs538628	17	44787313	G	C	-0.0537127	0.00920295	5.33E-09	6.60E-08	0.6738135
ARL17B	PD	Spinalcord eQTL	ENSG00000228696	17	46361797	rs199451	17	44801784	G	A	-0.202836	0.0332609	1.07E-09	6.42E-09	0.01223556
	AD	Multi Ancestry Whole Brain Meta-analysis eQTL	ENSG00000184922	17	43298811	rs62063276	17	44036408	G	T	-0.134405	0.0271688	7.53E-07	2.21E-06	0.6697149
FMNL1	PSP	Whole Blood mQTL	cg19481029	17	43314586	rs17630064	17	43289122	G	A	0.247221	0.0717373	5.69E-04	7.24E-08	0.06396753
	LBD	Whole Brain meta-analysis mQTL	cg08160350	4	996052	rs6599388	4	939087	C	T	-0.165404	0.030336	4.97E-08	2.81E-06	0.08410463
		Cortex eQTL metaBrain	ENSG00000127415	4	986997	rs73211813	4	975238	C	T	-0.435648	0.0846761	2.68E-07	2.39E-08	0.03864672
		Whole Blood eQTL GTEx	ENSG00000127415	4	980785	rs11248061	4	980896	A	C	-0.702144	0.152039	3.87E-06	4.08E-07	0.02435755
			cg00247629	4	995263	rs62294519	4	988619	C	T	-0.487929	0.122202	6.53E-05	2.00E-08	0.05213469
		Whole Blood mQTL	cg27494429	4	997540	rs11248060	4	964359	T	C	-0.486581	0.107127	5.57E-06	1.47E-07	0.07626291
			cg01962146	4	995931	rs1051613	4	951179	A	G	-0.165566	0.0343796	1.47E-06	8.18E-11	0.04819888
			cg08160350	4	996052	rs6599388	4	939087	C	T	-0.134337	0.0147854	1.03E-19	1.87E-12	0.01914885
			cg00187933	4	996785	rs11248061	4	980896	A	C	-0.235183	0.0490756	1.65E-06	3.55E-09	0.08509635
			cg15769764	4	996790	rs11248061	4	980896	A	C	-0.270404	0.0587199	4.12E-06	1.67E-07	0.09144947
IDUA	PD	Whole Brain meta-analysis mQTL	cg08332990	4	997351	rs11248061	4	980896	A	C	-0.248079	0.0525305	2.33E-06	1.16E-08	0.1446274
		Whole Blood eQTL eQTLgen	ENSG00000103510	16	31134894	rs1060506	16	31133449	T	C	-0.216715	0.0379747	1.15E-08	1.78E-07	0.1463961
		Whole Blood mQTL	cg02220965	16	31128310	rs1978487	16	31129942	C	T	0.0603758	0.0130409	3.66E-06	2.55E-07	0.06856892
		Whole Brain eQTL	ENSG00000103510	16	31134894	rs12597511	16	31145219	T	C	-0.079204	0.0178323	8.93E-06	1.45E-06	0.2647209
	AD	Whole Brain meta-analysis mQTL	cg02220965	16	31128310	rs4889619	16	31128615	T	C	0.0713321	0.0162194	1.09E-05	2.80E-07	0.1321537
		Cerebellar Hemisphere eQTL	ENSG00000103510	16	31127075	rs2855475	16	31147548	A	G	-0.233509	0.051988	7.07E-06	3.28E-07	0.02523191
		Cerebellum eQTL	ENSG00000103510	16	31114489	rs1549293	16	31141993	C	T	-0.17521	0.039031	7.16E-06	1.21E-06	0.04107506
		Cerebellum eQTL	ENSG00000103510	16	31127075	rs9972727	16	31149142	G	A	-0.176591	0.0390447	6.10E-06	1.04E-06	0.01264411
		Cortex eQTL GTEx	ENSG00000103510	16	31127075	rs1060506	16	31133449	T	C	-0.189104	0.0477015	7.36E-05	6.68E-07	0.03828886
		Hypothalamus eQTL	ENSG00000103510	16	31127075	rs61162043	16	31114234	G	A	-0.245687	0.0612799	6.09E-05	1.81E-06	0.2130376
		Skeletal Muscle eQTL	ENSG00000103510	16	31127075	rs9925964	16	31129895	G	A	-0.390776	0.0836696	3.01E-06	1.57E-07	0.0538749
		Tibial Nerve eQTL	ENSG00000103510	16	31127075	rs9936329	16	31140799	T	G	-0.428193	0.0892292	1.60E-06	3.76E-08	0.06971106
		Whole Brain eQTL	ENSG00000103510	16	31134894	rs12597511	16	31145219	T	C	-0.184809	0.0378713	1.06E-06	1.84E-07	0.04586272
KAT8	PD	Whole Brain meta-analysis mQTL	cg02220965	16	31128310	rs4889619	16	31128615	T	C	0.170253	0.0349027	1.07E-06	2.53E-06	0.5369356
		Amygdala eQTL	ENSG00000238083	17	44588877	rs2942166	17	43715427	C	T	-0.0465837	0.00988142	2.43E-06	2.54E-06	0.3929416
		Anterior Cingulate Cortex BA24 eQTL	ENSG00000238083	17	44588877	rs56971664	17	43918613	C	T	-0.0439728	0.00888721	7.50E-07	1.03E-06	0.6869415
		Caudate Basal Ganglia eQTL	ENSG00000238083	17	44588877	rs8073146	17	43893751	G	A	-0.0431091	0.00859267	5.25E-07	2.96E-06	0.4893544
		Cerebellum eQTL	ENSG00000238083	17	44588877	rs17564020	17	43991781	T	G	-0.0455583	0.0087789	2.11E-07	1.64E-06	0.8570875
		Cortex eQTL metaBrain	ENSG00000238083	17	46511511	rs2696466	17	44289832	A	G	-0.0586461	0.00950285	6.77E-10	3.80E-08	0.8992636
		Frontal Cortex BA9 eQTL	ENSG00000238083	17	44588877	rs4074462	17	43855228	T	G	-0.0430712	0.00872755	8.01E-07	1.50E-06	0.817564
		Hippocampus eQTL eQTLgen	ENSG00000238083	17	46511511	rs2696466	17	44289832	G	A	-0.0644059	0.0125498	2.87E-07	2.88E-06	0.5139333
		Hippocampus eQTL GTEx	ENSG00000238083	17	44588877	rs2942166	17	43715427	C	T	-0.0431814	0.00898806	1.55E-06	2.25E-06	0.3720312
		Hypothalamus eQTL	ENSG00000238083	17	44588877	rs8073146	17	43893751	G	A	-0.0404116	0.00803609	4.94E-07	1.59E-06	0.4894522
		Liver eQTL	ENSG00000238083	17	44588877	rs58879558	17	44095467	C	T	-0.0659929	0.0128493	2.81E-07	1.90E-06	0.1531949
		Multi Ancestry Whole Brain Meta-analysis eQTL	ENSG00000238083	17	44588877	rs2532329	17	44350090	A	G	-0.04872	0.00902299	6.68E-08	4.57E-08	0.9418131
		Nucleus Accumbens Basal Ganglia	ENSG00000238083	17	44588877	rs413917	17	43723189	A	G	-0.0432939	0.00858522	4.59E-07	6.97E-07	0.484854
		Prefrontal Cortex eQTL	ENSG00000238083	17	44588877	rs2696466	17	44289832	G	A	-0.0902575	0.0151182	2.37E-09	1.22E-08	0.04836808
		Putamen Basal Ganglia eQTL	ENSG00000238083	17	44588877	rs55942528	17	43932028	G	A	-0.0445283	0.00884181	4.75E-07	2.72E-06	0.7963627
		Skeletal Muscle eQTL	ENSG00000238083	17	44588877	rs4510068	17	44184828	T	G	-0.0624675	0.0102461	1.08E-09	1.15E-07	0.4008675
	AD	Whole Brain eQTL	ENSG00000238083	17	44610946	rs55825513	17	44176215	A	G	-0.0406017	0.0076394	1.07E-07	4.35E-08	0.02762345
		Amygdala eQTL	ENSG00000238083	17	44588877	rs2942166	17	43715427	C	T	-0.233466	0.0328069	1.11E-12	7.92E-11	0.3625708
		Basal Ganglia eQTL	ENSG00000238083	17	46511511	rs199530	17	44836653	G	A	-0.204835	0.0316577	9.78E-11	5.01E-10	0.01035425
		Frontal Cortex BA9 eQTL	ENSG00000238083	17	44588877	rs4074462	17	43855228	T	G	-0.206808	0.027368	4.14E-14	8.74E-12	0.4723248
		Hippocampus eQTL GTEx	ENSG00000238083	17	44588877	rs2942166	17	43715427	C	T	-0.216414	0.0290959	1.02E-13	1.43E-11	0.3236888
		Liver eQTL	ENSG00000238083	17	44588877	rs58879558	17	44095467	C	T	-0.273497	0.0391198	2.72E-12	1.24E-10	0.4735931
		Multi Ancestry Whole Brain Meta-analysis eQTL	ENSG00000238083	17	44588877	rs2532329	17	44350090	A	G	-0.22263	0.0255896	3.32E-18	1.22E-15	0.4841153
		Putamen Basal Ganglia eQTL	ENSG00000238083	17	44588877	rs9897399	17	43804317	G	A	-0.209757	0.027917	5.75E-14	1.07E-11	0.7115251
		Spinalcord eQTL	ENSG00000238083	17	46511511	rs58879558	17	44095467	T	C	-0.223417	0.0357817	4.27E-10	4.69E-09	0.2679779
LRRC37A2	PD	Substantia nigra eQTL	ENSG00000238083	17	44588877	rs55974014	17	43757450	A	C	-0.208719	0.0314696	3.30E-11	1.61E-09	0.5630028
		Anterior Cingulate Cortex BA24 eQTL	ENSG00000225190	17	43513266	rs55925547	17	43556807	C	T	-0.565533	0.120734	2.81E-06	1.03E-06	0.3394045
		Caudate Basal Ganglia eQTL	ENSG00000225190	17	43513266	rs79172804	17	43828764	A	G	-0.634594	0.119171	1.01E-07	2.22E-07	0.04643205
		Cortex eQTL GTEx	ENSG00000225190	17	43513266	rs10445367	17	43932798	T	G	-0.450407	0.0891061	4.31E-07	3.81E-09	0.03471772
		Cortex eQTL metaBrain	ENSG00000225190	17	45490749	rs55925547	17	43556807	T	C	-0.880801	0.155923	1.61E-08	2.82E-08	0.01207896
		Frontal Cortex BA9 eQTL	ENSG00000225190	17	43513266	rs79172804	17	43828764	A	G	-0.617712	0.125824	9.14E-07	1.19E-06	0.05384652
		Prefrontal Cortex eQTL	ENSG00000225190	17	43568110	rs79724577	17	43463493	C	A	1.20902	0.207821	5.97E-09	5.90E-09	0.01561697
		Putamen Basal Ganglia eQTL	ENSG00000225190	17	43513266	rs62065442	17	43563894	C	T	-0.545119	0.106568	3.13E-07	2.87E-07	0.03026128
		Skeletal Muscle eQTL	ENSG00000225190	17	43513266	rs12947718	17	43493101	A	G	-0.667265	0.0974424	7.50E-12	7.76E-12	0.03535971
	PD	Whole Brain eQTL	ENSG00000225190	17	43540690	rs113434679	17	44126765	A	C	0.545968	0.111957	1.08E-06	7.17E-07	0.3130195
PLEKHM1	PSP	Anterior Cingulate Cortex BA24 eQTL	ENSG00000225190	17	43513266	rs11012	17	43513441	T	C	3.97818	0.768359	2.25E-07	8.50E-08	0.487497
		Cerebellar Hemisphere eQTL	ENSG00000178226	16	31150246	rs78924645	16	31154358	A	G	-0.066351	0.0135831	1.04E-06	1.04E-06	0.5743859
		Cortex eQTL GTEx	ENSG00000178226	16	31150246	rs1549299	16	31154146	A	G	-0.0643026	0.012623	3.50E-07	1.83E-06	0.4465604
		Cortex eQTL metaBrain	ENSG00000178226	16	31150066	rs55667375	16	31155458	C	T	-0.0957689	0.0165264	6.84E-09	2.42E-06	0.4249718
		Multi Ancestry Whole Brain Meta-analysis eQTL	ENSG00000178226	16	31150246	rs78924645	16	31154358	A	G	-0.0889382	0.0151209	4.06E-09	2.10E-06	0.0123253
		Whole Brain eQTL	ENSG00000178226	16	31155830	rs1549299	16	31154146	A	G	-0.0732105	0.0149537	9.79E-07	9.79E-07	0.1419257
			cg07078430	16	31154357	rs78924645	16	31154358	A	G	-0.0865634	0.0176667	9.59E-07	9.59E-07	0.04135834
	AD	Whole Brain meta-analysis mQTL	cg00915858	16	31154636	rs55667375	16	31155458	C	T	0.0928108	0.0167849	3.21E-08	6.46E-07	0.01582905
PRSS36	PD	Whole Brain meta-analysis mQTL	cg00915858	16	31154636	rs55667375	16	31155458	C	T	0.137373	0.0357248	1.20E-04	2.18E-06	0.1779642
	AD	Cerebellum eQTL	ENSG00000185294	17	43922256	rs17573607	17	44083081	A	G	-0.071854	0.0146818	9.88E-07	2.71E-06	0.9201365
SPPL2C	PD	Prefrontal Cortex eQTL	ENSG00000185294	17	43922256	rs17577369	17	44192923	G	A	1.04066	0.182771	1.24E-08	1.30E-07	0.01842581
	LBD	Whole Blood mQTL	cg18586891	4	952366	rs11248059	4	960331	G	C	0.219928	0.0775702	4.58E-03	2.88E-06	0.06025584
		Cerebellum eQTL	ENSG00000127419	4	926175	rs73211813	4	975238	T	C	-0.275567	0.0574718	1.63E-06	7.71E-07	0.3303648
			cg01437235	4	939027	rs935977	4	943540	A	G	-0.183139	0.0360859	3.87E-07	7.83E-10	0.01008769
			cg10941006	4	942705	rs11724898	4	935933	A	G	-0.343495	0.0745804	4.11E-06	1.05E-09	0.01186439
		Whole Blood mQTL	cg02070118	4	952128	rs11248057	4	906131	G	C	0.495391	0.0721195	6.46E-12	4.33E-11	0.6479261
			cg15105011	4	940614	rs10008187	4	941017	T	C	-0.0888259	0.0169041	1.48E-07	4.41E-10	0.4333231
			cg23939001	4	940644	rs6848199	4	940838	T	C	-0.0868987	0.0165618	1.55E-07	1.17E-09	0.2607437
			cg20814179	4	940893	rs10008187	4	941017	T	C	-0.0876292	0.0166691	1.46E-07	6.69E-10	0.3099254
TMEM175	PD	Whole Brain meta-analysis mQTL	cg08222618	4	941054	rs10008187	4	941017	T	C	-0.0869293	0.0165502	1.50E-07	1.40E-09	0.2163939
	AD	Tibial Nerve eQTL	ENSG00000108379	17	44839872	rs199523	17	44848517	A	C	-0.0919719	0.0204807	7.10E-06	2.44E-06	0.03239027
		Cortex eQTL metaBrain	ENSG00000108379	17	46833058	rs9904865	17	44908263	T	C	-0.062473	0.0240469	9.38E-03	1.37E-08	0.09647706
WNT3	PD	Skeletal Muscle eQTL	ENSG00000108379	17	44839872	rs199523	17	44848517	A	C	-0.550915	0.105743	1.89E-07	8.52E-07	0.01487822

S9 - Known Class Genes

Omic	Disease	Gene	probeID	ProbeChr	ProbeBp	topRSID	topSNPChr	topSNPBp	A1	A2	beta, SMR	SE, SMR	p, SMR	p, multi-SNP SMR	p, HEIDI
Whole Brain meta-analysis mQTL	AD	MAPT	cg17569492	17	44026659	rs17650872	17	44039516	T	G	-0.0814127	0.0161079	4.32E-07	4.32E-07	0.6403735
Whole Brain meta-analysis mQTL	AD	MAPT	cg05721485	17	44071124	rs17651483	17	44058861	A	C	-0.0392778	0.00714939	3.93E-08	1.26E-06	0.1842054
Whole Brain meta-analysis mQTL	PD	MAPT	cg07163735	17	43971906	rs55780945	17	44040120	T	C	-0.485081	0.0918691	1.29E-07	1.29E-07	0.2039012
Whole Brain meta-analysis mQTL	PD	MAPT	cg21327887	17	43971928	rs56189701	17	43987801	C	T	-0.428248	0.0761027	1.83E-08	1.83E-08	0.3550347
Whole Brain meta-analysis mQTL	PD	MAPT	cg10780632	17	43973522	rs9303523	17	43976684	C	T	-0.190159	0.0331328	9.51E-09	1.65E-10	0.06329188
Whole Brain meta-analysis mQTL	PD	MAPT	cg19156875	17	43974446	rs17651507	17	44059010	A	T	0.232089	0.0584561	7.18E-05	1.83E-07	0.01714676
Whole Brain meta-analysis mQTL	PD	MAPT	cg20888521	17	43977917	rs17652121	17	44073973	C	T	-0.157605	0.0173742	1.18E-19	4.13E-13	0.02329478
Whole Brain meta-analysis mQTL	PD	MAPT	cg22291189	17	43978251	rs17652121	17	44073973	C	T	0.157605	0.0173742	1.18E-19	4.73E-14	0.04416005
Whole Brain meta-analysis mQTL	PD	MAPT	cg26019600	17	43978704	rs62063859	17	44078616	A	G	0.53072	0.106841	6.79E-07	6.79E-07	0.1094086
Whole Brain meta-analysis mQTL	PD	MAPT	cg12633764	17	43978756	rs62063859	17	44078616	A	G	0.395332	0.0645481	9.09E-10	1.72E-09	0.1586649
Whole Brain meta-analysis mQTL	PD	MAPT	cg05772917	17	44027251	rs17650872	17	44039516	T	G	-0.174153	0.0191833	1.10E-19	1.72E-13	0.01447168
Whole Brain meta-analysis mQTL	PD	MAPT	cg21705961	17	44060775	rs3785884	17	44057595	A	G	0.36317	0.0597238	1.20E-09	1.20E-09	0.1378246
Whole Brain meta-analysis mQTL	PD	MAPT	cg07368061	17	44090862	rs17574361	17	44108202	G	A	0.173968	0.0190538	6.83E-20	2.72E-13	0.06000518
Whole Brain meta-analysis mQTL	PSP	MAPT	cg10224600	17	43975063	rs1981997	17	44056767	A	G	1.14017	0.0815642	2.10E-44	2.10E-44	0.01425494
Whole Brain meta-analysis mQTL	PSP	MAPT	cg20888521	17	43977917	rs1981997	17	44056767	A	G	1.14017	0.0815642	2.10E-44	1.19E-31	0.08905337
Whole Blood mQTL	AD	MAPT	cg23202277	17	43971911	rs17689882	17	43906828	A	G	-0.130387	0.026718	1.06E-06	1.80E-06	0.04451087
Whole Blood mQTL	AD	MAPT	cg00891649	17	43972573	rs111751251	17	44042951	T	C	0.0760692	0.0144379	1.37E-07	3.08E-07	0.07318684
Whole Blood mQTL	AD	MAPT	cg18878992	17	43974344	rs17689882	17	43906828	A	G	-0.0678265	0.0127858	1.13E-07	3.97E-07	0.0158721
Whole Blood mQTL	AD	MAPT	cg14202850	17	43974869	rs117646503	17	43893260	C	T	-0.128522	0.0263587	1.08E-06	6.88E-07	0.2207176
Whole Blood mQTL	AD	MAPT	cg24801230	17	43978533	rs111751251	17	44042951	T	C	0.0343863	0.00628708	4.52E-08	1.81E-06	0.03870612
Whole Blood mQTL	AD	MAPT	cg18228076	17	43983362	rs111751251	17	44042951	T	C	0.0369295	0.00676406	4.77E-08	9.45E-07	0.02969893
Whole Blood mQTL	AD	MAPT	cg02228913	17	44058016	rs112572874	17	44072984	G	A	0.0385404	0.00711207	5.99E-08	2.08E-06	0.02802182
Whole Blood mQTL	AD	MAPT	cg00846647	17	44060252	rs111751251	17	44042951	T	C	0.0461684	0.00851495	5.89E-08	6.76E-07	0.04804293
Whole Blood mQTL	AD	MAPT	cg21705961	17	44060775	rs17689882	17	43906828	A	G	0.0721308	0.0136509	1.26E-07	2.53E-07	0.02723276
Whole Blood mQTL	AD	MAPT	cg01934064	17	44064242	rs2532233	17	44273218	T	C	-0.0982497	0.0191463	2.87E-07	4.34E-07	0.4999633
Whole Blood mQTL	AD	MAPT	cg07368061	17	44090862	rs62056790	17	43975417	A	G	0.0645877	0.0119984	7.32E-08	3.12E-07	0.09065659
Whole Blood mQTL	AD	MAPT	cg09764761	17	44105544	rs111541901	17	43994358	T	C	0.0724373	0.0134753	7.63E-08	2.69E-07	0.07306081
Whole Blood mQTL	PD	MAPT	cg23202277	17	43971911	rs112995313	17	43795768	C	T	-0.558183	0.0820831	1.04E-11	2.08E-10	0.1171783
Whole Blood mQTL	PD	MAPT	cg11909912	17	43974919	rs112197756	17	44154105	G	A	-1.03241	0.206365	5.65E-07	5.55E-07	0.1464477
Whole Blood mQTL	PD	MAPT	cg10224600	17	43975063	rs112197756	17	44154105	G	A	-0.789168	0.132521	2.60E-09	3.65E-08	0.2790191
Whole Blood mQTL	PSP	MAPT	cg05772917	17	44027251	rs11012	17	43513441	T	C	5.23144	0.831232	3.10E-10	6.22E-11	0.1366316
Skeletal Muscle eQTL	PD	ADORA2B	ENSG00000170425	17	15848231	rs1045599	17	15879910	T	C	0.0810002	0.0161604	5.38E-07	2.73E-06	0.0173546
Whole Blood eQTL eQTLgen	AD	KCNN4	ENSG00000104783	19	44278047	rs56681946	19	44283031	C	T	-0.0500123	0.0145726	0.0005992623	1.01E-08	0.1526556

S10 - Novel Genes Networks

Gene	Diseases	Omics	Number of Omics	Network Partners	Number of Network Partners	Potential network toxicity issues	Gene	Diseases
ADAM10	AD	Whole Brain meta-analysis mQTL, Whole Blood eQTL eQTLgen	2	CDH1, **NOTCH1**, **ERBB2**, **SP1**, **CD44**, ELAVL1, **EGF**, ELAVL4, ELAVL2, ELAVL3	10	None in immediate network	ADAM10	AD
SNCA	LBD	Whole Brain meta-analysis mQTL, Whole Blood mQTL, Whole Blood eQTL eQTLgen	3	**SYK, PPP2CB, CADPS2, PLK1, PRKN, CSNK1A1, PPP2CA, PLK3, DYRK1A, GRK2**	10	None in immediate network	SNCA	LBD
EGFR	AD	Whole Blood mQTL	2	**NCK1, LRRFIP1, MUC1, GALNT8, HGS, CAMK2A, PTPRJ, MAPK3, ADAM17, SOX2**	10	None in immediate network	POU5F1	AD
POU5F1	AD	Cortex eQTL metaBrain, Prefrontal Cortex eQTL	1	**DKK1, MAPK1, AKT1, NANOG, AKT2, MAPK3, LEFTY2, THY1, EOMES, HLX**	10	None in immediate network	EGFR	AD
STK39	PD	Whole Brain meta-analysis mQTL, Whole Blood mQTL	2	**MAPK14, CFTR, SLC4A4, WNK4, WNK1, SLC12A2, AATK, SLC12A3, SLC12A1**	9	None in immediate network	STK39	PD
INPP5D	AD	Whole Blood mQTL, Whole Blood eQTL eQTLgen, Whole Blood eQTL GTEx	3	**SHC1, ARRB2, PLCG2, SYK, TRAF6, GRB2**	6	None in immediate network	INPP5D	AD
CRHR1	PD, PSP, AD	Whole Brain meta-analysis mQTL, Whole Blood mQTL, Cortex eQTL metaBrain, Skeletal Muscle eQTL	4	**GNAS, GNAQ, CRH, GNAI1, POMC**	5	None in immediate network	CRHR1	PD, PSP, AD
APH1B	AD	Cortex eQTL metaBrain, Tibial Nerve eQTL, Skeletal Muscle eQTL, Whole Blood eQTL eQTLgen	4	**PSEN2, NCSTN, PSEN1, PSENEN**	4	None in immediate network	APH1B	AD
MINK1	AD	Whole Blood eQTL eQTLgen	1	**KRAS, KCNH2, PRICKLE1**	3	None in immediate network	MINK1	AD
CLU	AD	Whole Brain meta-analysis mQTL	1	MYBL2, **GDNF**, LRP2	3	None in immediate network	CLU	AD
CR1	AD	Cerebellum eQTL, Basal Ganglia eQTL, Cortex eQTL metaBrain, Cortex eQTL GTEx, Caudate Basal Ganglia eQTL, Whole Brain eQTL	6	C5AR1, **C5AR2**	2	None in immediate network	CR1	AD
ACE	AD	Whole Blood eQTL eQTLgen	6	CSNK2A1, **AGT**	2	None in immediate network	ITGAX	PD
CD38	PD	Cerebellum eQTL, Whole Brain meta-analysis mQTL, Whole Blood mQTL, Cerebellar Hemisphere eQTL, Cortex eQTL GTEx, Tibial Nerve eQTL, Skeletal Muscle eQTL, Hypothalamus eQTL, Whole Blood eQTL eQTLgen, Whole Brain eQTL, Cerebellum eQTL	6	**OXT, PECAM1**	2	OXT - ALS	KAT8	PD, AD
RABEP1	AD	Whole Blood eQTL eQTLgen	1	**RAB5A, RABGEF1**	2	None in immediate network	ERCC2	AD
ERCC2	AD	Cerebellum eQTL, Whole Blood mQTL, Cortex eQTL metaBrain, Prefrontal Cortex eQTL, Whole Blood eQTL eQTLgen, Whole Brain eQTL	1	**ERCC3, ERCC5**	2	None in immediate network	ACE	AD
KAT8	PD, AD	Whole Blood eQTL eQTLgen	11	KMT2A, **FASN**	2	None in immediate network	RABEP1	AD
ITGAX	PD	Cortex eQTL metaBrain, Prefrontal Cortex eQTL, Cortex eQTL GTEx, Caudate Basal Ganglia eQTL, Whole Brain eQTL, Nucleus Accumbens Basal Ganglia	1	FGA, **ICAM1**	2	None in immediate network	CD38	PD
GAK	PD	Whole Blood mQTL, Skeletal Muscle eQTL	2	**SRC**	1	None in immediate network	STX4	PD
STX4	PD	Whole Blood mQTL	2	STXBP4	1	None in immediate network	EPHA1	AD
EPHB4	AD	Whole Blood eQTL eQTLgen	1	EFNB1	1	None in immediate network	EPHB4	AD
EPHA1	AD	Whole Brain meta-analysis mQTL, Whole Brain eQTL	1	**EFNA1**	1	None in immediate network	GAK	PD
GPNMB	PD	Skeletal Muscle eQTL, Whole Blood eQTL eQTLgen	14	No curated network partners	0	None in immediate network	VKORC1	PD
STAG3	AD	Whole Blood mQTL	14	No curated network partners	0	None in immediate network	CDSN	AD
CHRNE	AD	Whole Brain meta-analysis mQTL	2	No curated network partners	0	None in immediate network	MAT1A	AD
NDUFS2	AD	Whole Blood mQTL	4	No curated network partners	0	None in immediate network	SLC44A4	AD
FCER1G	AD	Whole Brain meta-analysis mQTL	3	No curated network partners	0	None in immediate network	EPHX2	AD
VKORC1	PD	Whole Blood mQTL	2	No curated network partners	0	None in immediate network	PSORS1C1	AD
DNTT	AD	Cerebellum eQTL, Whole Brain meta-analysis mQTL, Cortex eQTL metaBrain, Frontal Cortex BA9 eQTL, Prefrontal Cortex eQTL, Cortex eQTL GTEx, Caudate Basal Ganglia eQTL, Multi Ancestry Whole Brain Meta-analysis eQTL, Hypothalamus eQTL, Anterior Cingulate Cortex BA24 eQTL, Whole Blood eQTL eQTLgen, Putamen Basal Ganglia eQTL, Whole Brain eQTL, Nucleus Accumbens Basal Ganglia	1	No curated network partners	0	None in immediate network	GPNMB	PD
CKM	AD	Whole Brain meta-analysis mQTL	1	No curated network partners	0	None in immediate network	MS4A2	AD
HSD3B7	PD	Whole Brain meta-analysis mQTL, Whole Blood mQTL, Cortex eQTL metaBrain, Frontal Cortex BA9 eQTL, Cerebellar Hemisphere eQTL, Caudate Basal Ganglia eQTL, Tibial Nerve eQTL, Hippocampus eQTL, Substantia nigra eQTL, Hypothalamus eQTL, Whole Blood eQTL eQTLgen, Amygdala eQTL, Cerebellum eQTL, Nucleus Accumbens Basal Ganglia	2	No curated network partners	0	None in immediate network	STAG3	AD
BST1	PD	Whole Blood mQTL	1	No curated network partners	0	None in immediate network	MICB	AD
STX1B	PD	Whole Blood mQTL	1	No curated network partners	0	None in immediate network	STX1B	PD
PSMC3	AD	Whole Blood mQTL	1	No curated network partners	0	None in immediate network	PSMC3	AD
CDSN	AD	Whole Blood mQTL, Cortex eQTL GTEx	1	No curated network partners	0	None in immediate network	CHRNE	AD
MICB	AD	Whole Blood eQTL eQTLgen	1	No curated network partners	0	None in immediate network	BST1	PD
MS4A2	AD	Prefrontal Cortex eQTL, Skeletal Muscle eQTL	1	No curated network partners	0	None in immediate network	HSD3B7	PD
PSORS1C1	AD	Whole Blood mQTL, Cortex eQTL metaBrain, Whole Blood eQTL eQTLgen, Whole Brain eQTL	1	No curated network partners	0	None in immediate network	NDUFS2	AD
EPHX2	AD	Whole Blood eQTL eQTLgen	1	No curated network partners	0	None in immediate network	CKM	AD
SLC44A4	AD	Skeletal Muscle eQTL, Whole Blood eQTL eQTLgen, Whole Blood eQTL GTEx	1	No curated network partners	0	None in immediate network	FCER1G	AD
MAT1A	AD	Whole Blood eQTL eQTLgen	1	No curated network partners	0	None in immediate network	DNTT	AD
FBXL19	PD	Whole Blood mQTL	1	No curated network partners	0	None in immediate network	FBXL19	PD

Source	Ancestry	rsID	Allele1	Allele2	Effect	P-value
Wu et al. (2017)	Taiwanese	rs3793947	A	*n/a*	*n/a*	0.508
Xu et al. (2016)	Chinese	rs156429	A	A	*n/a*	0.108
Rizig et al. (under preperation)	African & African Admix	rs1637190	G	A	-0.0808	0.1338
		rs858275	C	T	-0.0824	0.125
		rs858274	C	T	-0.0522	0.5875
		rs858239	G	A	0.0494	0.6099
		rs199357	G	A	-0.0271	0.6602
		rs199355	G	A	-0.0122	0.8008
		rs199348	C	A	-0.0042	0.931
		rs199351	C	A	-0.0335	0.7277
		rs466240	C	T	-0.0232	0.8135
		rs858273	C	T	0.054	0.5768
Present Study	multiancestry	rs858275	T	C	-0.107745	1.08E-08
	European	rs1637190	A	G	0.124869	2.00E-07
		rs199357	A	G	0.118241	9.66E-07
		rs858239	A	G	0.24504	1.94E-08
		rs199357	A	G	0.126337	2.20E-06
		rs858273	T	C	0.128654	2.65E-06
		rs199351	C	A	0.132703	2.88E-06
		rs858273	T	C	0.155196	1.40E-06
		rs858274	T	C	0.0887275	3.21E-08
		rs199357	A	G	0.179231	1.88E-08
		rs199348	C	A	0.117214	1.61E-08
		rs199355	G	A	-0.237456	1.94E-06
		rs199357	G	A	-0.181814	2.46E-07
		rs199357	G	A	-0.17763	5.79E-07
		rs466240	T	C	-0.078348	2.84E-08
		rs199357	G	A	-0.0900894	1.89E-08
		rs199357	G	A	-0.0901975	2.45E-08
		rs199357	A	G	0.131601	1.20E-06
		rs199357	A	G	0.121781	8.08E-07
