# Supplementary material for: omicSynth: An open multi-omic community resource for identifying druggable targets across neurodegenerative diseases
Source: Am J Hum Genet. 2024 Jan 4;111(1):150–64. doi: 10.1016/j.ajhg.2023.12.006 (PMC10806756; doi:10.1016/j.ajhg.2023.12.006)
Supplement: Document S2. Article plus supplemental information [file mmc3.pdf]

omicSynth: An open multi-omic community resource for identifying druggable targets across neurodegenerative diseases

Graphical abstract

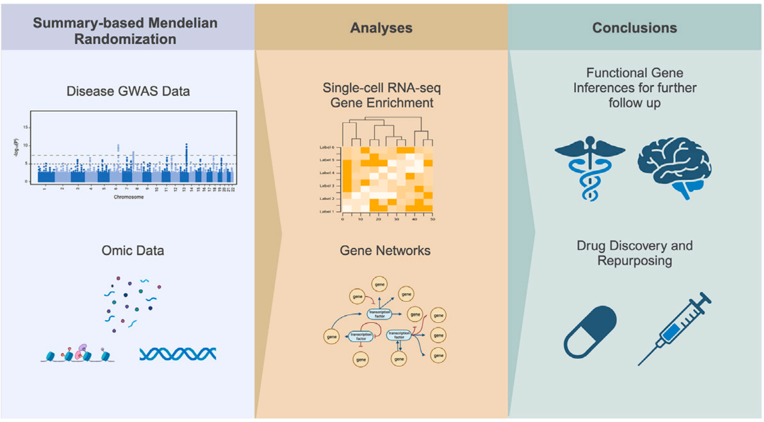

Authors

Chelsea X. Alvarado,
Mary B. Makarious, Cory A. Weller, ...,
Faraz Faghri, Mike A. Nalls,
Hampton L. Leonard

Correspondence

hampton@datatecnica.com

Neurodegenerative diseases (NDDs) affect millions of people, and finding genetically supported and effective drug targets has been difficult. Omic data availability and statistical methods can help nominate potential drug targets. We identified 54 NDD-related genes for further functional follow up and drug repurposing.

Alvarado et al., 2024, The American Journal of Human Genetics *111*, 150–164
January 4, 2024 © 2023 The Author(s).
https://doi.org/10.1016/j.ajhg.2023.12.006

CellPress

ARTICLE

omicSynth: An open multi-omic community resource for identifying druggable targets across neurodegenerative diseases

Chelsea X. Alvarado,[1,2] Mary B. Makarious,[3,4,5] Cory A. Weller,[1,2] Dan Vitale,[1,2] Mathew J. Koretsky,[1] Sara Bandres-Ciga,[1] Hirotaka Iwaki,[1,2,3] Kristin Levine,[1,2] Andrew Singleton,[1,3] Faraz Faghri,[1,2,3] Mike A. Nalls,[1,2,3] and Hampton L. Leonard[1,2,3,6,*]

Summary

Treatments for neurodegenerative disorders remain rare, but recent FDA approvals, such as lecanemab and aducanumab for Alzheimer disease (MIM: 607822), highlight the importance of the underlying biological mechanisms in driving discovery and creating disease modifying therapies. The global population is aging, driving an urgent need for therapeutics that stop disease progression and eliminate symptoms. In this study, we create an open framework and resource for evidence-based identification of therapeutic targets for neurodegenerative disease. We use summary-data-based Mendelian randomization to identify genetic targets for drug discovery and repurposing. In parallel, we provide mechanistic insights into disease processes and potential network-level consequences of gene-based therapeutics. We identify 116 Alzheimer disease, 3 amyotrophic lateral sclerosis (MIM: 105400), 5 Lewy body dementia (MIM: 127750), 46 Parkinson disease (MIM: 605909), and 9 progressive supranuclear palsy (MIM: 601104) target genes passing multiple test corrections ($p_{SMR\_multi} < 2.95 \times 10^{-6}$ and $p_{HEIDI} > 0.01$). We created a therapeutic scheme to classify our identified target genes into strata based on druggability and approved therapeutics, classifying 41 novel targets, 3 known targets, and 115 difficult targets (of these, 69.8% are expressed in the disease-relevant cell type from single-nucleus experiments). Our novel class of genes provides a springboard for new opportunities in drug discovery, development, and repurposing in the pre-competitive space. In addition, looking at drug-gene interaction networks, we identify previous trials that may require further follow-up such as riluzole in Alzheimer disease. We also provide a user-friendly web platform to help users explore potential therapeutic targets for neurodegenerative diseases, decreasing activation energy for the community.

Introduction

Currently, there are few approved disease-modifying therapeutics available to those with a neurodegenerative disease (NDD), the most recent being lecanamab for the treatment of Alzheimer disease.[1] NDDs such as Alzheimer disease (AD), Parkinson disease (PD), amyotrophic lateral sclerosis (ALS), Lewy body dementia (LBD), frontotemporal lobar degeneration (FTLD [MIM: 607485]), and progressive supranuclear palsy (PSP) are diseases caused by progressive nerve cell degeneration that result in a loss of cognition and/or motor function.[2] The World Health Organization (WHO) expects dementia diagnoses alone to reach 78 million by 2030 and 139 million by 2050. Without disease-modifying therapies, the health, social, and economic impacts of dementia and related NDDs will be catastrophic.[3] The identification of rational therapeutic targets for NDDs will require both the generation of new data and the development and deployment of rapid, open, and transparent tools.

Drugs that are supported by genetic or genomic data frequently outperform those without such evidence in clinical trials. Over two-thirds of the US Food and Drug Administration (FDA)-approved drugs in 2021 were supported by genetic or genomic evidence.[4] Therapeutics with genetically supported target mechanisms are twice as likely to pass a trial phase as those without supporting genetic data are.[5] Given the importance of anchoring therapeutic targets to a disease mechanism substantiated by genetic evidence, we developed omicSynth: a dynamic, open, and accessible resource that leverages large-scale genetic and genomic data for the identification of therapeutic targets in the NDD space.

The omicSynth resource integrates genetic and genomic data in a summary-data-based Mendelian randomization (SMR) framework.[5] The SMR framework facilitates functional inferences relating disease risk (from genome-wide association studies [GWASs]) to the underlying mechanism (from quantitative trait loci [QTL] variant data in relevant tissues) through their approach to Mendelian randomization (MR). MR uses instrumental variables (genetic variants) to test for a causative effect of an exposure, such as gene expression, on an outcome (disease phenotype). The SMR approach to MR tests for pleiotropic association

[1]Center for Alzheimer's and Related Dementias (CARD), National Institute on Aging and National Institute of Neurological Disorders and Stroke, National Institutes of Health, Bethesda, MD 20814, USA; [2]Data Tecnica LLC, Washington, DC 20037, USA; [3]Laboratory of Neurogenetics, National Institute on Aging, National Institutes of Health, Bethesda, MD 20814, USA; [4]Department of Clinical and Movement Neurosciences, UCL Queen Square Institute of Neurology, London WC1N 3BG, UK; [5]UCL Movement Disorders Centre, University College London, London WC1N 3BG, UK; [6]German Center for Neurodegenerative Diseases (DZNE), Tübingen, Germany
*Correspondence: hampton@datatecnica.com
https://doi.org/10.1016/j.ajhg.2023.12.006.
© 2023 The Author(s). This is an open access article under the CC BY license (http://creativecommons.org/licenses/by/4.0/).

between the exposure and outcome. Pleiotropic association is defined by Zhu et al. as the association between an outcome (disease phenotype) and exposure (QTLs) due to either pleiotropy (both QTLs and the disease phenotype are affected by the same causal variant) or causality (the effect of a causal variant on the disease phenotype is mediated by the QTLs). In order to distinguish pleiotropy from linkage, the accompanying heterogeneity in dependent instruments (HEIDI) method utilizes *cis*-QTLs surrounding the probe being tested to distinguish pleiotropy from linkage. The resulting SMR effect size (beta) and HEIDI values measure the effect of the exposure on the outcome free of any non-genetic confounders and the probability of the tested genetic variant being consistent with linkage, respectively.[5] In an SMR association as shown here, the effect estimates correspond to functional inferences where a positive association suggests concordance between increased omic levels and increased risk and a negative beta suggests the inverse. In many analyses, the same associations can have opposite directions for the same gene in different tissues or in different disease contexts. The data utilized are GWASs from population-scale resources, composed of millions of samples across multiple NDDs, and omic data composed of QTL studies measuring methylation, gene expression, chromatin state, and protein expression. Additionally, we incorporated expression-QTL (eQTL) data from genetically diverse backgrounds into our SMR analyses, addressing the lack of multi-ancestry data in NDD research and allowing for limited functional inferences regarding differences between ancestral populations.

To add additional context to nominated gene targets, we also investigated single-nucleus data from disease-enriched cell types.[6,7] Many therapeutics mechanistically target inhibition of expression, thus, it is necessary that the target is expressed at baseline in the relevant cell types for these treatments to be effective in addressing a particular disease. Using single-nucleus expression data, we investigated whether the nominated genes could potentially be affected by inhibitors that can cross the blood-brain barrier, as in general, genes need to be expressed in order to be susceptible to inhibition from a mechanistic perspective.

We prioritize identified genes as therapeutic targets of interest into three classes based upon known small-molecule druggability and product market information (Figure 1). Novel targets include genes that exhibit significant functional inferences in relevant tissue and cell types in druggable regions of the genome, are not currently targeted by disease-specific therapeutics, and should be prioritized in future repurposing studies and drug development. Known targets include genes within relevant tissue and cell types that have documented significant functional inferences but are already impacted by a known drug that specifically targets an NDD. Difficult target genes are not in regions of the genome currently annotated as druggable. For all novel targets, we searched the corresponding gene regulatory networks to identify companion genes that could also be useful as therapeutic targets. Potential upstream and downstream effects on targeting these genes for therapeutic intervention were provided based on network memberships, and toxicity within these networks was inferred by evaluating liver eQTLs within the network as well as known interacting drugs for each gene of interest. Results can be browsed through the omicSynth resource, which is made available via a free web-based platform, further decreasing activation energy for therapeutic target discovery within the research community (https://nih-card-ndd-smr-home-syboky.streamlit.app/).

Material and methods

Datasets

GWAS summary statistics for each of the six NDDs highlighted in our study were used to obtain single nucleotide polymorphisms (SNPs) that served as instrumental variables in the MR pipeline. GWASs used are the latest and/or largest for each corresponding disease: Bellenguez et al. for AD (n = 788,989); Chia et al. for LBD (n = 7,372); Höglinger et al. for PSP (n = 4,361); Nalls et al. for PD (n = 1,456,306); Nicolas et al. for ALS (n = 80,610); and Pottier et al. for FTLD (n = 1,355).[8–13]All GWAS summary statistics were lifted over, as needed, to hg19 (GRCh37) using University of California, Santa Cruz's liftOver command line tool.[14]

QTL summary statistics

eQTLs, protein QTLs (pQTLs), chromatin QTLs (caQTLs), and methylation QTLs (mQTLs) were used as the exposure variables in the MR analyses. eQTLs are genetic loci that explain the variation in mRNA expression levels. *Cis*-eQTL, eQTLs that act on local genes, data make up most all QTL data used for our study because of the volume of publicly available data sources. All eQTL and mQTL data obtained, except from the sources eQTLgen, metaBrain, and Zeng et al. (multi ancestry), were already in SMR format and obtained from the Yang Lab's Data Resource page.[15–17] The eQTL sources from the Yang Lab include Genotype-Tissue Expression (GTEx) project v8 release, PsychENCODE, and BrainMeta v1 (formerly brain-eMeta).[18–20] The specific tissues measured varied by data source but consisted of NDD-related tissues, which we have defined as brain, nerve, muscle, blood, and liver tissues. Liver was included because of its role in metabolizing medications, toxicity, and potential impacts on clinical trial progress.[21]

mQTLs are genetic variants that affect methylation patterns of CpG sites. mQTL data sources include Brain-mMeta and McRae et al., which are derived from blood tissues.[20,22] We included caQTLs—caQTLs alter traits by modifying chromatin structure—data from Bryois et al. in our analysis.[23] Blood tissues have been shown to have high correlation in expression levels with brain tissues, allowing blood tissues to provide a gain of power and ease of use in biomarker studies due to the relative ease of availability of this tissue.[20] All genome positions are mapped to the human reference genome build hg19 (GRCh37).

pQTLs are genetic variants associated with protein expression levels. Similarly to eQTLs and mQTLs, pQTLs can be used as our exposure variable. We obtained pQTL summary statistics data from Yang et al.[24] The pQTLs are from plasma, brain, and cerebrospinal fluid (CSF) tissues from participants with and without AD. Samples are on human reference genome build hg19 (GRCh37). More details on the samples and methods used can be found in

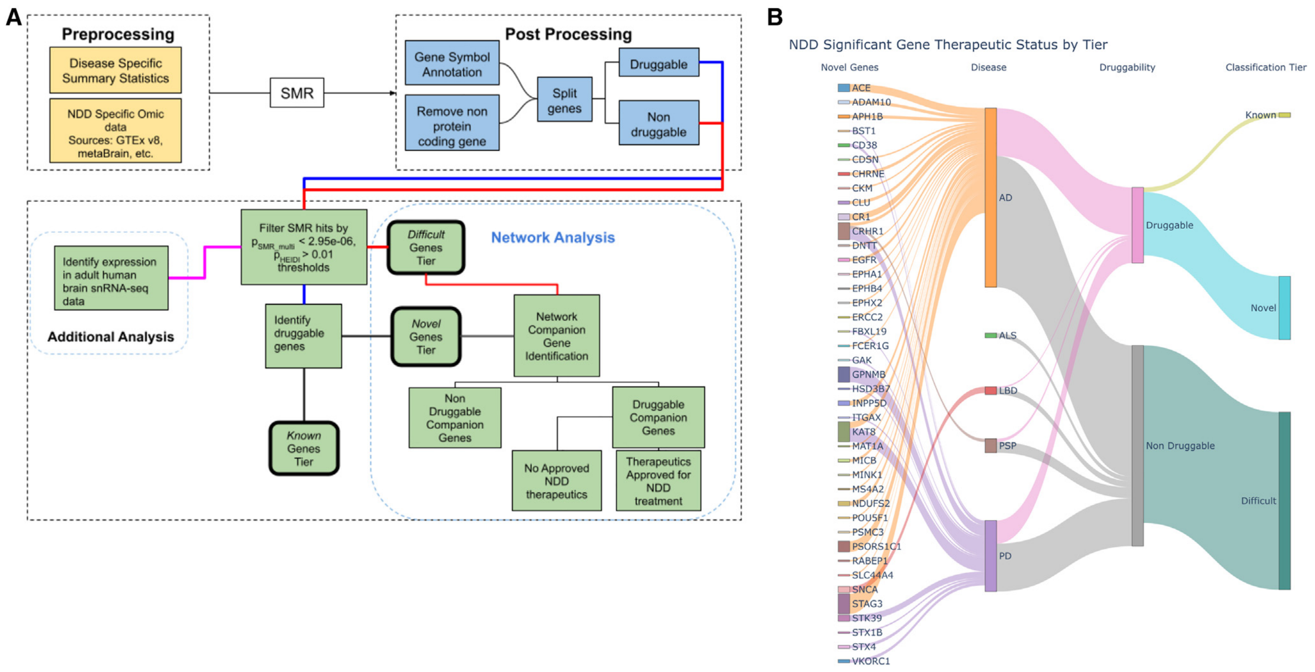

Figure 1. Graphical representation of research workflow and results

(A) Graphical representation of the general workflow used in conducting our analyses. NDD, neurodegenerative disease; SMR, summary-data-based Mendelian Randomization. (B) Graphical summary of results. Sankey plot depicting the flow of candidate genes into their respective tier. On the left, we highlight novel genes, but the remainder of the plot visualizes all 159 candidate genes regardless of the final classification tier. Note: the size of each novel gene node is scaled to represent the number of significant SNPs for the associated gene probe-disease pairing.

the original manuscript.[24] pQTLs were nominated for inclusion for SMR analysis if they were significant ($p < 0.05$) in at least one of the three tissues per the original manuscript. We included 453 unique pQTLs across the three tissues: 223 pQTLs from CSF, 159 from plasma, and 77 from the brain.

Gene expression summarization

We summarized expression ranks for genes of interest within the single-cell adult human brain transcriptome dataset adult_human_20221007.loom from Siletti et al.[6] Using custom R scripts, we converted feature counts into TPM (transcripts per million). For a given sample, feature counts were divided by maximum nonredundant intron-removed exon lengths to correct for differences in gene length. Values were then multiplied by a sample-specific constant (10^6/T, where T is the sum of length-normalized counts) such that the resulting unitless vector sums to one million. We extracted exon lengths based on annotations from the gene transfer format (GTF) file used to originally annotate the single-cell data (gb_pri_annot.gtf). We calculated the expression percentile rank (EPR) for genes of interest using the empirical cumulative distribution function and then calculated the mean and median EPR value for each gene across cells of each tested cell type. To ease interpretation, we binned the EPR values into 3 classes—off, low, and high (off: EPR <10, low: $10 <$ EPR <90, high: EPR >90) by using the mean single-nucleus RNA sequencing (snRNA-seq) EPR of each gene against cell type.

Gene-gene networks

Data were obtained from the Open Targets.[25] Open Targets provides an API to cross reference annotations and relationships on diseases, genes, and drugs. Companion genes were pulled from the SIGnaling Network Open Resource (SIGNOR) database due to the manual curation of gene interactions.[26]

Therapeutic drug data

Therapeutic drug data were obtained from various sources including Finan et al. ("druggable genome") and the Drug Gene Interaction Database (DGIdb).[27,28] Druggable genome data were obtained from the supplementary materials in Finan et al. The obtained data provided insight on 3,000+ potential gene targets with evidence for drug targets or potential targets.[27] DGIdb drug data (accessed January 2023) were downloaded from the DGIdb online database as files consisting of known gene and drug interactions as well as details such as interaction types and drug categories.

Pre-processing

All data pre-processing was carried out using custom scripts for data that were not obtained via the Yang Lab or was missing information such as gene symbols. Pre-processing included gene annotation, binary with expression summary data (BESD) format preparation and conversion, and/or calculation of necessary measures such as beta values. Gene annotation was performed as necessary if no gene symbol was provided in the original data source. Annotation was conducted by using both the Python biomart package and pyensembl with Ensembl gene (ENSG) IDs. mQTL gene annotation was conducted by obtaining Illumina 450k chip probe data via the R package IlluminaHumanMethylation450kanno.ilmn12.hg19.[29–31]

Data sources were converted as necessary into BESD format using the flist method outlined by the Yang Lab. BESD format stores QTL summary data in a set of three files: .esi, .epi, and .besd. More information on the format and how to process data into BESD

format can be found on the SMR Yang Lab website listed in the key resources table.

Our multi-ancestry eQTL data originally lacked allele frequency, beta, or standard error values. Missing allele frequencies were obtained using the 1000 Genomes reference panel from which we derived beta and standard error values using each eQTL's random effects model Z score, allele frequency, and total number of samples from the original study ($n = 2,119$).

Summary-data-based Mendelian randomization

SMR is an MR computational tool that uses summary-level data to test if an exposure variable (i.e., gene expression) and outcome (i.e., disease phenotype) are causally associated because of a shared causal variant (i.e., instrumental variable).[5] To discern potentially causal variants from those in linkage disequilibrium with the functional variant, the HEIDI method was implemented using the default flag that uses the top 20 SNPs within 500 kb of the probe.[5] Linkage disequilibrium reference data were obtained from 1000 Genomes Project phase 3 reference panel.[32] To increase statistical power, we applied the SMR-multiple exposures (SMR-multi) method, a Bayesian framework for simultaneous testing of multiple traits or exposures on a single outcome while accounting for the correlation between them. SMR and HEIDI analysis were conducted using the SMR software established and maintained by the Yang Lab using all default parameters, including those previously detailed.[5,33]

Following SMR, we filtered results to include only protein-coding genes and removed potential associations with no available gene annotations or associations with genes in the major histocompatibility complex (MHC). We used a significance threshold of $p_{SMR\_multi} < 2.95 \times 10^{-6}$, corresponding to the Bonferroni-corrected value at $\alpha = 0.05$ for 16,875 protein-coding genes tested across all NDDs and omic pairs. We then filtered results based on the presence of inferred pleiotropy via the computed HEIDI score ($p_{HEIDI} > 0.01$ for inclusion in this study).[5] SNPs were then split on their associated genes status as a therapeutic target or as a non-therapeutic target. After initial processing, analyses were conducted as demonstrated in our workflow diagram (Figure 1A) and explained further in our gene nomination workflow below. In total, we tested a total of 186 omic-tissue pairs across six NDDs (Table 1).

Gene nomination and drug target identification

Gene nomination focused on targets shared by multiple NDDs, classifying targets by inferred druggability as described in the introduction. Therapeutic targets were initially nominated using data from DGIdb and Finan et al.[27,28] Further target curation was conducted using Open Targets to verify if any approved indications included an NDD thus allowing us to classify drugs into either the novel or known tiers. Identified network companion genes upstream and downstream of the initial target identified were further categorized into groups based on therapeutic status and approved use in treating any NDD. Our known and difficult tiers were further investigated using gene co-expression networks via Open Targets interaction annotations through the SIGNOR database. Using SIGNOR-identified interactions, we identified companion genes, i.e., those manually annotated for their causal relationships with the gene of interest. We additionally searched known therapeutics that target any identified companion genes to potentially identify proxy gene targets thus expanding the net for drug discovery and repurposing. We implemented custom python scripts to query Open Targets' application programming interface (API) to extract relevant annotations for this workflow.

Results

Overview

We identified 540 candidate gene-level SMR associations (159 unique gene targets) across six NDDs and 186 tissue-omic pairings with a stringent disease-level multiple test correction threshold ($p_{SMR\_multi} < 2.95 \times 10^{-6}$ and $p_{HEIDI} > 0.01$; Tables S1, S2, and S4). On a per-disease basis we identified 317 total significant associations: 116 unique gene targets for AD, 4 significant associations across 3 unique gene targets for ALS, 13 significant associations across 5 unique genes for LBD, 184 significant associations across 46 unique gene targets for PD, and 22 significant associations across 9 unique gene targets for PSP. FTLD had no significant associations at our corrected p value threshold. No NDDs showed significant pQTL or caQTL associations. Of the 159 unique genes across all diseases, 69.8% (111 genes) were expressed in tissues enriched with disease-associated gene expression, and 11.9% (19 genes) showed colocalization with brain tissue eQTLs with posterior probability (PP) >0.90.[7] Colocalized genes from this complementary analysis are also identified in Tables 2 and 3.

SMR analysis identifies 15 common genes significant across multiple NDDs

Using SMR, we identified 15 unique genes across 182 associations to be significant in two or more NDDs at a stringent significance threshold ($p_{SMR\_multi} < 2.95 \times 10^{-6}$ and $p_{HEIDI} > 0.01$; Tables S2, S3, and S4). Of the identified genes, five genes (*MAPT* [MIM: 157140], *CRHR1* [MIM: 122561], *KANSL1* [MIM: 612452], *ARL17A*, and *ARHGAP27* [MIM: 610591]) were found to be significant across 97 tested associations and three NDDs (AD, PD, and PSP). *MAPT* and *CRHR1* were found to be largely significant in mQTL omics with *MAPT* significant in whole blood and brain mQTL data for all previously mentioned NDDs, and *CRHR1* was found to be significant in whole blood mQTL data for all three NDDs (Tables S3, S4, and S5). Additionally, only *MAPT* and *CRHR1* are annotated as druggable in multiple drug data sources as of the writing of this manuscript. All genes, except for *ARL17A*, had multiple significant associations in both brain and blood mQTL tissues. *ARHGAP27* and *KANSL1* had significant associations replicated in the multi-ancestry eQTL data (African, American, East Asian, European, and South Asian ancestries), suggesting a potential generalizability of these targets across different populations.

We identified 10 unique genes across 85 significant associations in any two NDDs ($p_{SMR\_multi} < 2.95 \times 10^{-6}$ and $p_{HEIDI} > 0.01$). One of the identified genes is considered therapeutic, and the remaining nine are non-therapeutic. *KAT8* (MIM: 609912), *ARL17B*, *PRSS36* (MIM: 610560), *LRRC37A2* (MIM: 616556), *WNT3* (MIM: 165330), and *SPPL2C* (MIM: 608284) were all found to be significant in

Table 1. **Summary of SMR data mining across NDDs**

Disease	Total genes (unique)	Liver genes	Total eQTL genes (non-multi-ancestry)	Replicated in multi-Ancestry	Total druggable genes	% Druggable	Total non-druggable genes	% Non-druggable
**All tested genes (protein coding)**								
AD	16,833	1,597	15,112	8,404	3,562	21.2%	13,271	78.8%
ALS	16,875	1,610	15,163	8,408	3,565	21.1%	13,310	78.9%
FTLD	16,788	1,537	15,038	8,394	3,551	21.2%	13,237	78.8%
LBD	16,797	1,540	15,069	8,388	3,554	21.2%	13,243	78.8%
PD	16,872	1,596	15,159	8,407	3,566	21.1%	13,306	78.9%
PSP	16,042	1,033	13,839	8,073	3,420	21.3%	12,622	78.7%
**Significance $p_{\_SMR\_multi} < 0.05$ and $p_{\_HEIDI} > 0.01$**								
AD	8	175	3,189	2,079	1,142	14,275.0%	3,806	47,575.0%
ALS	3,188	83	1,857	1,260	715	22.4%	2,473	77.6%
FTLD	2,318	78	1,243	810	542	23.4%	1,776	76.6%
LBD	2,530	82	1,384	900	580	22.9%	1,950	77.1%
PD	3,592	108	2,161	1,434	811	22.6%	2,781	77.4%
PSP	2,275	30	1,270	842	574	25.2%	1,701	74.8%
**Significance $p_{\_SMR\_multi} < 2.95E{-}06$ (testing all protein coding genes) and $p_{\_HEIDI} > 0.01$**								
AD	116	2	68	7	31	26.7%	85	73.3%
ALS	3	0	3	0	0	0.0%	3	100.0%
FTLD	0	0	0	0	0	0.0%	0	0.0%
LBD	5	0	1	0	1	20.0%	4	80.0%
PD	46	3	33	5	15	32.6%	31	67.4%
PSP	9	0	5	3	2	22.2%	7	77.8%
**Significance $p_{\_SMR\_multi} < 1.58E{-}08$ (testing all protein coding genes across all omics) & $p_{\_HEIDI} > 0.01$**								
AD	47	1	19	19	14	29.8%	33	70.2%
ALS	1	0	0	0	0	0.0%	1	100.0%
FTLD	0	0	0	0	0	0.0%	0	0.0%
LBD	2	0	0	0	1	50.0%	1	50.0%
PD	24	1	14	14	8	33.3%	16	66.7%
PSP	8	0	5	5	2	25.0%	6	75.0%

AD, Alzheimer disease; ALS, amyotrophic lateral sclerosis; FTLD, frontotemporal dementia lobar degeneration; LBD, Lewy body dementia; PD, Parkinson disease; PSP, progressive supranuclear palsy.

both AD and PD; *IDUA* (MIM 252800) and *TMEM175* (MIM: 616660) were found to be significant in LBD and PD; *PLEKHM1* (MIM: 611466) was significant in PD and PSP; and *FMNL1* (MIM: 604656) was significant in AD and PSP (Tables S3, S4, and S5). *KAT8*, the only therapeutic gene, showed significant associations with decreased expression in brain tissue and blood, as well as a significant association with increased methylation. Of the remaining nine genes, *IDUA*, *FMNL1*, *PRSS36*, and *TMEM175* had significant associations in mQTL sources. Additionally, *FMNL1*, *LRRC37A2*, and *PRSS36* had significant associations replicated in the multi-ancestry eQTL data ($p_{SMR\_multi} < 2.95 \times 10^{-6}$; Table S7).

Drug target discovery using significant genes identifies 41 novel gene targets for follow-up study

Using the approach previously outlined in our introduction and methods for drug target gene nomination, we categorized 159 gene hits into one of three tiers (Table 3). In our first tier, novel genes, we nominated 41 gene targets. SMR results for the novel genes are listed in Table S8. Genes are categorized as novel if they are in druggable regions of the genome that can be targeted by common molecular methods and currently have no FDA-approved treatment for any NDD as identified by current literature, knowledge base, and drug databases. Our second tier, known genes, had three gene targets identified—*MAPT*, *KCNN4*

Table 2. Candidate genes for multiple NDDs

Gene	Diseases	Omics
ARL17B	AD, PD	cerebellum eQTL, cortex eQTL, spinalcord eQTL
KAT8	AD, PD	cerebellum eQTL, whole-brain meta-analysis mQTL, cerebellar hemisphere eQTL, cortex eQTL, tibial nerve eQTL, skeletal muscle eQTL, hypothalamus eQTL, whole-brain eQTL, cerebellum eQTL, spinalcord eQTL
LRRC37A2	AD, PD	hippocampus eQTL, cortex eQTL, frontal cortex BA9 eQTL, prefrontal cortex eQTL, caudate basal ganglia eQTL, skeletal muscle eQTL, multi-ancestry, whole-brain meta-analysis eQTL, hypothalamus eQTL, liver eQTL, anterior cingulate cortex BA24 eQTL, putamen basal ganglia eQTL, amygdala eQTL, whole-brain eQTL, cerebellum eQTL, nucleus accumbens eQTL, basal ganglia eQTL, spinalcord eQTL, hippocampus eQTL, substantia nigra eQTL
KANSL1	AD, PD, PSP	whole-brain meta-analysis mQTL, whole-blood mQTL, cortex eQTL, multi-ancestry whole-brain meta-analysis eQTL, spinalcord eQTL, anterior cingulate cortex BA24 eQTL
ARL17A	AD, PD, PSP	spinalcord eQTL, amygdala eQTL, multi-ancestry whole-brain meta-analysis eQTL, hypothalamus eQTL, hippocampus eQTL, cerebellar hemisphere eQTL, cortex eQTL, caudate basal ganglia eQTL, anterior cingulate cortex BA24 eQTL, putamen basal ganglia eQTL, cerebellum eQTL, nucleus accumbens basal ganglia
PRSS36	AD, PD	whole-brain meta-analysis mQTL, cortex eQTL, cerebellar hemisphere eQTL, multi-ancestry whole-brain meta-analysis eQTL, whole-brain eQTL
MAPT	AD, PD, PSP	whole-brain meta-analysis mQTL, whole-blood mQTL
IDUA*	LBD, PD	whole-brain meta-analysis mQTL, whole-blood mQTL, whole-blood eQTL(eQTLgen)
TMEM175*	LBD, PD	whole-blood mQTL
ARHGAP27	AD, PD, PSP	whole-blood mQTL, whole-blood eQTL (eQTLgen), multi-ancestry whole-brain meta-analysis eQTL, caudate basal ganglia eQTL, nucleus accumbens basal ganglia
CRHR1	AD, PD, PSP	whole-brain meta-analysis mQTL, whole-blood mQTL, cortex eQTL, skeletal muscle eQTL
FMNL1	AD, PSP	multi-ancestry whole-brain meta-analysis eQTL, whole-blood mQTL
PLEKHM1	PD, PSP	cortex eQTL, frontal cortex BA9 eQTL, prefrontal cortex eQTL, caudate basal ganglia eQTL, skeletal muscle eQTL, anterior cingulate cortex BA24 eQTL, putamen basal ganglia eQTL, whole-brain eQTL
WNT3	AD, PD	cortex eQTL metaBrain, skeletal muscle eQTL, tibial nerve eQTL
SPPL2C	AD, PD	cerebellum eQTL, prefrontal cortex eQTL

This table shows genes with functional inferences passing multiple test corrections for multiple neurodegenerative diseases. We provide details for all the omics and diseases in which a given gene has significant associations. Asterisks indicate colocalized genes.

(MIM: 602754), and *ADORA2B* (MIM: 600446). Known genes are genes that have at least one FDA-approved therapeutic for treatment of an NDD (Table S9). The 3 nominated known gene targets are targeted by four therapeutics—apomorphine, carbidopa, istradefylline, and riluzole. Currently, these therapeutics are used for the treatment of PD symptoms (apomorphine, carbidopa, istradefylline) and prolonged survival for ALS (riluzole). In our last and largest tier, difficult genes, we identified 115 gene targets with no currently known therapeutics that target these genes and no known druggability. A total of 52 of the identified difficult genes exhibited at least two significant associations, with *LRRC37A2* having the maximum number of significant associations at 25 associations across AD and PD.

Network analysis provides insight into druggable companion genes to non-druggable genes of interest

We further implemented a gene network analysis for our novel and difficult tier candidates to identify potential proxy gene targets within each nominated genes' SIGNOR curated network. In the novel gene tier, we identified 87 companion genes of which 58 are considered potentially druggable (Table S9). Of the 58 druggable companion genes, 30 were found to be targeted by a known drug, and a further five are targeted by therapeutics approved for treatment of AD. The five companion genes with AD-targeted therapeutics are *NCSTN* (MIM: 605254), *MAPK14* (MIM: 600289), *PSEN1* (MIM: 104311), *PSEN2* (MIM: 600759), and *PSENEN* (MIM: 607632), which are all targeted by tarenflurbil, semagacestat, and avagacestat. *MAPK14* is targeted by neflamapimod, an oral p38 alpha kinase inhibitor that the FDA approved for use in the treatment of AD and LBD (Figure 2). Further analysis of difficult gene co-expression networks identified 27 genes with 65 curated companion genes (Tables S10, S11, and S12; Figure S1). Of the 65 identified companion genes for the difficult target tier, 34 were found to be druggable with 18 having known drugs. *MAPK14* was the only companion

Table 3. Therapeutic classification scheme by tier

Tier	Requirements	Number of genes	Genes
Novel	druggable; not approved for use in treating NDDs	41	ADAM10, SNCA, EGFR, POU5F1, STK39, INPP5D, CRHR1, APH1B, MINK1, CLU, CR1, ACE, CD38, RABEP1, ERCC2, KAT8, ITGAX, GAK, STX4, EPHB4, EPHA1, GPNMB, STAG3, CHRNE, NDUFS2, FCER1G, VKORC1, DNTT, CKM, HSD3B7, BST1, STX1B, PSMC3, CDSN, MICB, MS4A2, PSORS1C1, EPHX2, SLC44A4, MAT1A, FBXL19
Known	druggable; approved for use in treating NDD	3	MAPT, KCNN4, ADORA2B
Difficult	no known druggability	115	TRIM27, PPP4C, SPI1, EFNA3, KIF1C, WNT3, CD2AP*, CCNE2, KCTD13, C9orf72*, SRCAP, CELF1, HIP1R*, GRN*, APOC2, ARHGAP27, MEPCE, LRRFIP2, COPS6, GIGYF1, BCKDK, POLR2E, EFNA4, DYDC1, ATF6B, LLGL1, MTMR2, GPC2*, LRRC37A, ARL17B, INO80E*, SNX31, CEACAM19*, DGKQ*, NUP42, LRRC37A2, KANSL1, ARL17A, ANXA11, TSPAN14, CASTOR3, ZNF232, ZNF45, TSBP1, TREM2, PRSS36*, IDUA*, CCDC158, CCDC189, ZSWIM7, PLEKHM1, STH, PVRIG, YPEL3, MMRN1*, SPPL2C, SCIMP, PILRB*, PILRA, LACTB*, FMNL1, APOC4, ZNF646, CPSF3, ZSCAN9, ZKSCAN3, TREML2, EPDR1, UFSP1, FAM131B, TAS2R60*, USP6NL, MS4A4A, CASS4, G2E3, SCFD1, PCGF3*, SETD1A, DCAKD, ZNF668, AGFG2, TMEM175*, TOMM40, TRIM40, WDR81, TMEM106B, FNBP4, SHROOM3, CYP21A2*, REXO1, TNXB, MS4A3, AIF1, RAB8B, ZFP57, FAM200B, BTNL2, IGSF9B*, HS3ST1, ZNF311, NDUFAF6, TMEM163*, APOC1, C17orf107, EXOC3L2, DYDC2, DOC2A, ACMSD, TRIM31, PRDM7, TRIM10, ZAN, MS4A6A, CPLX1, SFTA2

Table providing information on the three classifications tiers in our therapeutic classification scheme including requirements for each tier. Asterisks indicate colocalized genes.

gene to have a therapeutic approved (neflamapimod) to treat an NDD in the difficult target tier. *MAPK14* was identified as a companion gene to the difficult gene *TRIM27* (MIM: 602165) (Figure 2).

Drug toxicity analyses were conducted using liver eQTL data as a proxy. No novel gene was found to show significant SMR expression in liver eQTL data. However, using companion genes, we identified *OXT* (MIM: 167050), a companion to the novel gene *CD38* (MIM: 107270), to be significantly expressed in liver eQTL in the context of ALS. While *CD38* was found to be significant in AD and PD but not ALS, it is a consideration that should be considered as comorbidity may exist between ALS and AD or PD. *TOMM40* (MIM: 608061) is the only difficult gene to show significance in liver eQTL data. There are no companion genes to a difficult gene with significant expression in liver eQTL data.

Multi-ancestry analyses reveal opposing gene expression patterns in significant disease risk loci between non-European and European ancestries

To investigate our findings in more diverse data, we also performed SMR with multi-ancestry eQTL data, aiming to replicate previous significant results ($p_{SMR\_multi} < 2.95 \times 10^{-6}$ and $p_{HEIDI} > 0.01$) and nominate targets that show evidence toward generalizability across multiple ancestry groups. After multiple test corrections, we significantly replicated 11 total associations corresponding to 9 genes that were nominated in our initial analysis: *ARHGAP27*, *ARL17A*, *GPNMB* (MIM: 604368), *KANSL1*, *LRRC37A2*, *PILRA* (MIM: 605341), *PILRB* (MIM: 605342), *PRSS36*, and *ZNF232* (MIM: 616463) (see Table S7).

We then categorized these nine replicated genes based on their druggable status. One of the replicated hits, *GPNMB*, is classified in our novel gene tier, meaning that it is druggable and has no known treatment approved for treatment of NDDs, which suggests this gene may be an interesting but also potentially generalizable target across ancestries. The remaining eight replicated genes were in our difficult tier, meaning they are currently considered non-druggable through the small molecule modality.

Nominated genes of interest found to be expressed in disease-relevant adult human brain snRNA-seq expression data

To provide additional evidence for biological relevance of our nominated targets, we investigated whether the 159 significant genes nominated through SMR ($P_{SMR\_multi} < 2.95 \times 10^{-6}$ & $P_{HEIDI} > 0.01$) were expressed in relevant cell types from adult human brain snRNA-seq data.

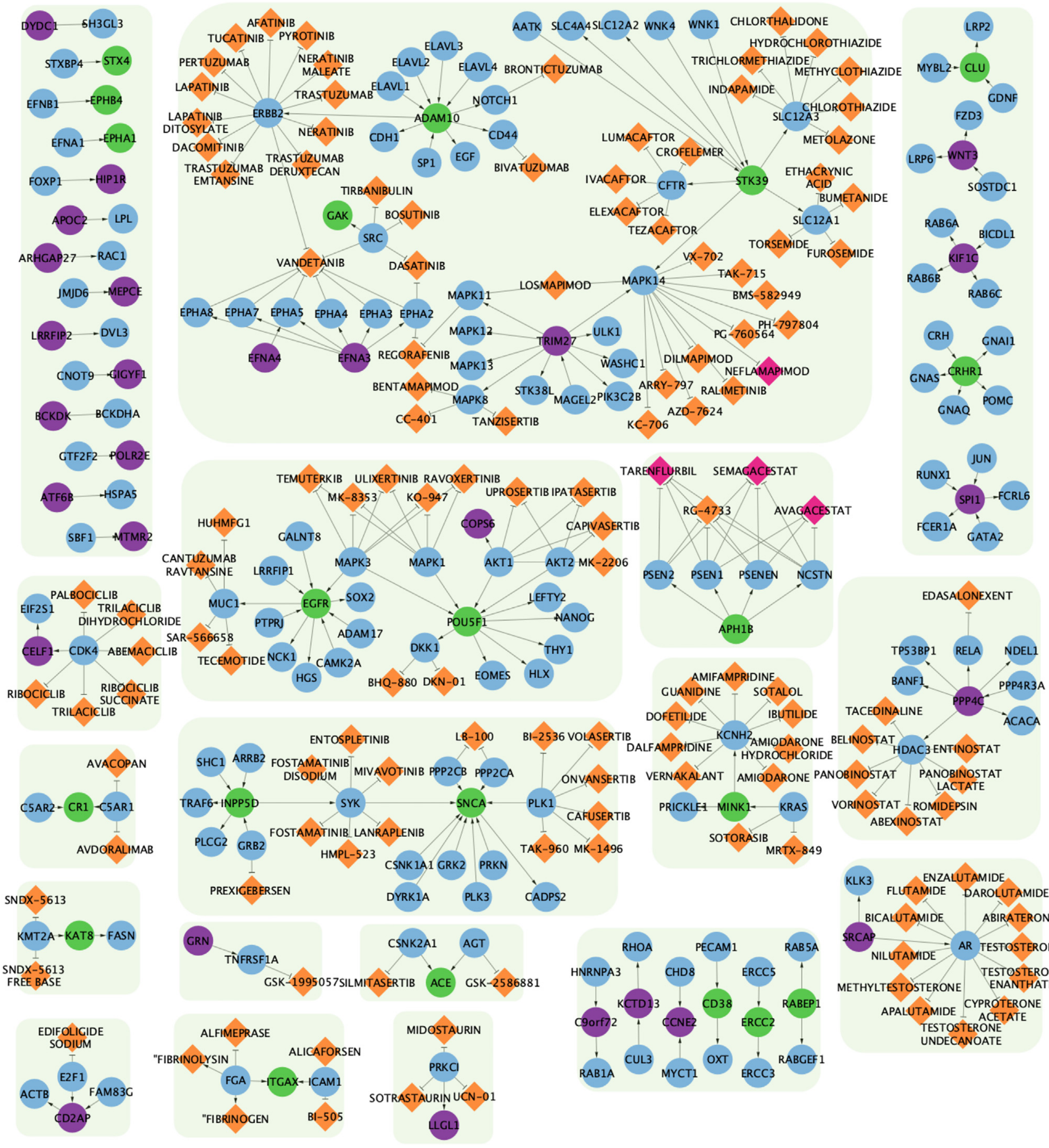

Figure 2. Network visualization of novel and difficult genes, companion genes, and drugs
Graph network visualization of both novel (green nodes) and difficult (purple nodes) genes and their SIGNOR-curated partners (blue nodes). Drugs that interact with companion genes are denoted by orange-colored diamonds while FDA-approved drugs for use in NDD treatment are colored pink. Connecting arrows indicate the direction from regulator gene to target gene. Note: green boxes surrounding the gene-drug groupings serve as a visual guide to help separate non-connected groups.

We tested the 159 genes found to be significant against adult human snRNA-seq data to identify expression in varying brain single-cell types ($P_{SMR\_multi} < 2.95 \times 10^{-6}$ & $P_{HEIDI} > 0.01$). We calculated the mean and median EPR for each gene across cells corresponding to each of the 31 tested cell types. To ease interpretation, we additionally binned the mean EPR values into three expression categories: off, low, and high based on the mean EPR value for each gene-cell-type combination. Using our binned mean EPR values, we found that 40 genes had all exclusively off EPR values while 16 genes had exclusively all low EPR values across all 31 tested cell types (Figure 3;

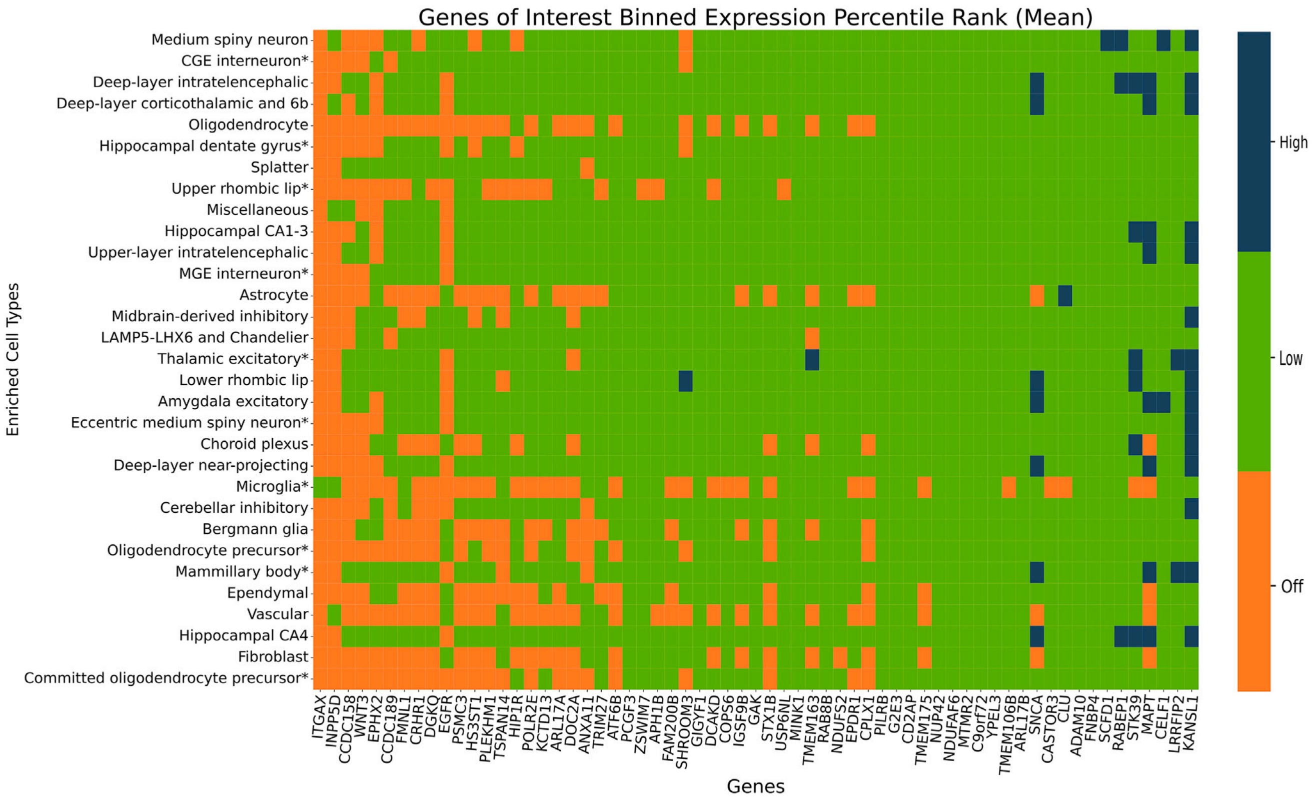

Figure 3. Single-cell RNA sequencing expression for significant genes ($p_{SMR\_multi} < 2.95 \times 10^{-6}$ and $p_{HEIDI} > 0.01$)
Expression of a given gene within each cell type is categorized as being highly expressed (dark blue, top 10% of all genes), intermediately expressed (green, middle 80%), or undetected (orange, bottom 10%). Genes that had off mean EPR values across all tested cell types were excluded from the heatmap. Cell types found to be enriched with AD and/or PD-relevant genes are marked by an asterisk. All other NDDs did not show significant cell type enrichment in any tissue, partially impacted by the much-smaller reference disease GWAS sizes.

Tables S13 and S14). We identified 11 genes (*KANSL1*, *LRRFIP2* [MIM: 614043], *CELF1* [MIM: 601074], *MAPT*, *STK39* [MIM: 607648], *RABEP1* [MIM: 603616], *SCFD1* [MIM: 618207], *CLU* [MIM: 185430], *SNCA* [MIM: 163890], *TMEM163* [MIM: 618978], and *SHROOM3* [MIM: 604570]) that had at least one gene highly expressed in any single cell type, with *KANSL1* having the most high EPR values across 15 cell types (Figure 3; Tables S13 and S14). Detailed breakdowns of both mean and median EPR values are provided in Figures S2–S4 and Tables S13 and S14. Cell types hippocampal CA4 and deep-layer intratelencephalic had the most genes with high EPR values, and hippocampal CA4 had the least number of genes with off EPR values ($n_{high} = 5$, $n_{low} = 89$, $n_{off} = 65$). The vascular cell type had the highest number of genes with off values ($n_{off} = 123$).

We also investigated the nominated genes in disease-relevant cell types—Bergmann glia, caudal ganglionic eminence (CGE) interneuron, committed oligodendrocyte precursor, deep-layer intratelencephalic, eccentric medium spiny neuron, hippocampal CA1–3, hippocampal dentate gyrus, LAMP5, LHX6 and chandelier, medial ganglionic eminence (MGE) interneuron, mammillary body, microglia, midbrain derived inhibitory, oligodendrocyte precursor, thalamic excitatory, upper layer intratelencephalic, and upper rhombic lip—identified in Alvarado et al. to be significantly enriched with AD- and PD-relevant genes.[7] Significant genes from our SMR analysis were highly expressed in three of these disease-relevant cell types: thalamic excitatory, eccentric medium spiny neuron, and mamillary body. *KANSL1* had high expression in all three listed cell types (Figure 3). Of the 159 tested genes, 20 had all low EPR values in the disease-relevant cell types. The thalamic excitatory cell type had the least number of off EPR values for genes *INPP5D* (MIM: 601582), *ITGAX* (MIM: 151510), *EGFR* (MIM: 131550), and *DOC2A* (MIM: 604567).

Discussion

As the global population continues to age, the threat posed by NDDs presents an overwhelming and multifaceted challenge. Our research aims to address the challenge of treating NDDs by identifying therapeutic targets anchored in genetic data—a proven strategy in therapeutic development. Our conservative approach primarily focuses on small-molecule drug targets given the breadth of available data as this class of therapeutics has the most studied and reliable gene-based annotations available. Implementation

of this strategy has been impeded by the small sample sizes and the dispersed nature of genetic- and disease-related data, such as proteomics and transcriptomics. Here, we attempted to address this need by creating and implementing an open-source framework to identify druggable targets across varied NDDs.—→

In our targeted analyses, we were unable to identify any potentially functionally relevant genes that were significant across all six tested NDDs. While NDDs share prominent hallmarks, such as cell death, inflammation, and pathological protein aggregation, the role that each hallmark and its associated biological processes play in the pathogenesis of each NDD differs, creating a spectrum.[2,34] We identified *MAPT*, *CRHR1*, *KANSL1*, *ARL17A*, and *ARHGAP27* to be independently significant in multiple different omics for AD, PD, and PSP (Tables S3, S4, and S5). *MAPT* was found to have significant associations with primarily increased expression for AD, PD, and PSP across eQTL and mQTL omic data, as supported by previous research.[35–37] The *MAPT* locus, 17q21, contains genes *CRHR1*, *KANSL1*, *ARL17A*, and *ARHGAP27*, and mutations in this locus have been previously associated with both PD and PSP.[38] Due to the complexity and observed linkage disequilibrium found in the 17q21 locus and the limitations of the SMR framework, causality cannot be established or inferred without further functional follow up. Previous evidence of significant association of this locus in AD is more fragmented and sparser. The 17q21 locus, which includes genes *KANSL1* and *MAPT*, has been previously implicated in AD.[39] *ARL17A* has been reported to harbor eQTL SNPs implicated in both brain and blood tissues in relation to AD.[40] *CRHR1*'s role in stress response has been hypothesized to exacerbate AD pathologies given its abundance in the brain, including areas implicated in learning and memory.[41] Lastly, evidence of *ARHGAP27*'s significance in AD includes associations between complex traits such as cognitive functioning, reaction time, and cortical structure phenotypes.[42,43]

A deep dive into *KANSL1* highlights its role in autophagy pathways. *KANSL1* is a core member of the non-specific lethal (NSL) complex that binds to *MOF* (also known as *KAT8*), which is necessary for the acetylation of histone H4 lysine 16 acetylation (H4K16ac).[44,45] Some studies have associated elevated expression levels of *KANSL1* with over-promoted autophagic activity, resulting in cell death and cytotoxicity from autophagosome accumulation; however, further research is required to understand this mechanism.[46] Additional research into the role of autophagy and lysosomal pathways in NDDs have indicated that altered autophagy function results in the inability to clear out protein aggregates, resulting in cell death and potentially contributing to disease pathogenesis and neurodegeneration.[45,47–49] Our results are consistent with previous research, linking increased expression of *KANSL1* with neurodegenerative effects. When assessing associations with AD, PD, and PSP, *KANSL1* is associated with an increased expression in brain mQTLs, three different brain eQTLs (psychEncode, multi-ancestry, and anterior cingulate cortex), and spinal cord eQTLs. The consistent significance of *KANSL1* and most of our gene hits in mQTL omics highlights the influence of DNA methylation for NDD pathogenesis and progression.

We identified 10 genes as significant in two diseases. The nominated genes do not share any explicit relationships but are common in their importance for varying biological processes and cellular functions, such as cell proliferation and differentiation, degradation of transmembrane proteins, calcium homeostasis, and autophagy regulation.[50–54] Six of our nominated genes, *ARL17B*, *KAT8*, *LRRC37A2*, *PRSS36*, *SPPL2C*, and *WNT3*, are associated with both AD and PD. Given the significantly larger sample sizes and increased power of the two diseases in GWAS summary statistics, we did not find this unexpected. LBD and PD share two genes, *IDUA* and *TMEM175*, while AD and PSP share *FMNL1*, and PD and PSP share *PLEKHM1* (Tables S3, S4, and S6). In general, the bulk of the gene hits were found to be significant in mQTL data for both brain and blood tissues ($n_{\text{whole brain}} = 4$; $n_{\text{whole blood}} = 4$) followed by cortex eQTLs ($n_{\text{cortex metaBrain}} = 6$, $n_{\text{cortex GTEx}} = 3$, $n_{\text{Frontal Cortex BA9}} = 2$, $n_{\text{prefrontal cortex}} = 3$).

The only gene found in two diseases, AD and PD, that could be targeted therapeutically was *KAT8*, which we previously mentioned in the context of the *KANSL1* gene. In literature, *KAT8* (lysine acetyltransferase 8) is identified as a protein-coding gene that plays a vital role in the NSL complex for acetylation of H4K16ac.[49] Scientific observation has identified the consequences of autophagic dysfunction in NDDs to include impaired neuronal function, neuronal death, and neuron loss. In opposition to the expression pattern of *KANSL1*, decreased expression of *KAT8* is associated with deacetylation of H4K16ac in AD patients, while an overexpression of *KAT8* has been linked to increased expression levels of neuroprotective soluble amyloid precursor protein (sAPP)α and β-secretase (BACE)2 and decreased levels of sAPPβ and *BACE1* (MIM: 604252).[55] In our results, we found blood mQTLs for AD and brain mQTLs for AD and PD to be associated with increased expression of *KAT8*; this is in contrast to gene expression in some of the same tissues, such as blood and brain mQTLs, for *KANSL1*. The associated increased expression of *KAT8* in our results suggests that an increase in expression may be correlated with excess autophagy resulting in cell death, which is a hallmark symptom of all three NDDs (AD, PD, and LBD).[56,57] There are currently no FDA-approved therapeutic that target *KAT8* in NDDs. However, compound MG149, a histone acetyltransferase inhibitor, has been found to reduce proinflammatory genes via inhibition of MYST (named for protein members MOZ, Ybf2/Sas3, Sas2, and Tip60)-type histone acetyltransferase *KAT8*.[58] MG149 has also been found to be effective in restoring impaired autophagic flux via the inhibition of histone acetylation of H4K16ac in cases of ischemic stroke and inflammatory diseases.[48,59] Further research into the application of MG149 could result in a

novel treatment targeting the characteristic accumulation of toxic proteins in NDDs.

FTLD was the only tested disease that did not have any suggestive targets at our test correction threshold. This may be because the FTLD GWASs had the smallest sample size out of all the diseases tested, and results will likely improve as larger FTLD GWASs are conducted. As there were no significant results for FTLD after correction, we decided to investigate potential pleiotropic relationships between FTLD and the other NDDs. To do this, we looked for FTLD associations at a less-stringent p value threshold ($p_{SMR\_multi} < 0.05$) only in the 254 unique candidate genes passing our original threshold of $p_{SMR\_multi} < 2.95 \times 10^{-6}$, a process detailed by Baird et al.[60] This resulted in 124 FTLD hits made up of 31 unique genes that have a potential pleiotropic relationship between FTLD and another NDD. Of those 31, 12 were classified as druggable through our sources (*STX4* [MIM: 186591], *STX1B* [MIM: 601485], *VKORC1* [MIM: 608547], *POU5F1* [MIM: 164177], *HSD3B7* [MIM: 607764], *PSORS1C1* [MIM: 613525], *SLC44A4* [MIM: 606107], *CD38*, *EPHX2* [MIM: 132811], *FBXL19* [MIM: 609085], *CLU*, and *CDSN* [MIM: 602593]). All 12 fall into the novel tier of drug targets, representing potential avenues for drug repurposing for FTLD.

Our creation of a drug target classification scheme is an attempt to inform drug discovery and repurposing from genes considered significant with evidence of causative roles in NDDs. Further inspection of our 41 novel genes provides multiple insights into the genes that compose the tier. Many genes that compose our novel tier have therapeutics used in the treatment of multiple types of cancers and tumors. Fourteen of our novel genes have therapeutics approved for use in the treatment of cancer (MONDO_0004992). Other commonly approved indications for therapeutics that target our novel genes include, but are not limited to, neoplasm (EFO_0000616), hypertension (EFO_0000537 [MIM: 145500]), and cardiovascular disease (EFO_0000319). *GPNMB*, which is of particular interest due to support for its role in PD, falls into this grouping of 14 genes. Similar to its role in cancer and tumor growth, our results highlight *GPNMB*'s pattern of increased expression as shown in brain-related PD eQTLs. We were able to find replication of increased GPNMB expression in brain-related tissues in Li et al., Ortiz et al., and Nalls et al.[61–63] Glembatumumab vedotin is one of the therapeutics that targets *GPNMB* where its primary mechanism of action (MOA) is tubulin inhibition.[25] Consequently, glembatumumab vedotin's inhibitory MOA could be repurposed for use in PD treatment for suppression of inflammation given the recognized role of inflammatory response/neuroinflammation in PD onset and progression.[64,65] However, any treatment developed targeting *GPNMB* would most likely be limited in treating people of European ancestries due to the gene's importance and role compared to non-European ancestries—further increasing inequality.

Our largest and most uncertain classification tier contains 121 difficult genes. Despite not having any currently known therapeutics, this classification tier could lead to the development of NDD-targeted therapeutics or the repurposing of existing ones. Our approach for these genes focused on analyzing well curated networks centered on each difficult gene to identify any partner genes with existing therapeutic drugs. This approach provides us context into any biological pathways and processes that may be affected by a targeted treatment, which could help eliminate the time and resources spent on developing and researching ineffective therapies.

The smallest tier, known genes, is composed of the three genes targeted by NDD-targeted therapeutics. Apomorphine, carbidopa, and istradefylline are indicated for use in treatment of PD. Riluzole is indicated for the treatment of ALS but has undergone phase 2 clinical trials for use in treatment of AD. The results in clinical trials for use of riluzole in AD treatment were promising with cerebral glucose metabolism, an AD biomarker, preserved in patients receiving riluzole compared to those in the placebo group.[66] The researchers conducting the study suggested a more powerful and longer study, but no follow up studies have yet been initiated. Our results support the continued follow up of riluzole clinical trials.

Focusing on genes we flagged as putatively associated with risk across multiple diseases, 13 of 15 were noted as being at least moderately expressed in cell types of interest (those with enriched expression for GWAS risk signatures) from single-nucleus sequencing. Positive beta coefficients at these genes from the SMR analysis suggest that if an expression effect was inhibited, it could be possible to reduce disease risk. Two of these genes, *KANSL1* and *MAPT*, showed significant positive associations (defined as a gene with positive beta values in more than 50% of its significant SMR associations) between risk and expression in our SMR analyses, providing a contextual insight for future follow up.

Genes such as *GPNMB* had different expression patterns in European and non-European ancestries. For example, *GPNMB* had decreased associated expression in multi-ancestry eQTLs but an increased associated expression in all other tested eQTLs. Previous research in certain Asian populations has found no significant association between *GPNMB* and PD.[67,68] Rizig and colleagues, conducting the largest PD GWAS in the African and African admixed populations in ~200,000 individuals, of which 1,488 are cases, report the following per SNP in *GPNMB*: rs858275, $p = 0.1250$, beta = –0.0824, indicating no association in African/African admixed ancestries. Our multi-ancestry data report the same direction of expression in GPNMB SNP rs858275, $p = 1.080397 \times 10^{-8}$, beta = –0.107745 in PD. Interestingly, the reported direction of expression in our multi-ancestry data and Rizig and colleagues' data contrasts with the direction of expression reported for European ancestries in addition to indicating no significant associations (Table S13).[69]

The limitations we encountered in our research included limited GWAS data for diseases, excluding AD and PD,

limited non-eQTL omic data, limited multi ancestry omic data, and reference panels, as well as non-small-molecule drug target annotations. In general, the availability of public and free omic and drug target data is increasing. As new data are published, we intend to conduct updates and incorporate new omic types into our analysis, such as more pQTL, single-cell QTLs, and splicing QTLs (sQTLs). This was a hypothesis generating effort at scale, and while there are too many results for us to follow up in detail ourselves, we hope that the single-nucleus enrichments will help guide others to the correct cell types for their studies and further target nomination efforts. The incorporation of additional multi-omic data should provide new and novel insights into the complex underpinnings of NDDs, while incorporating additional data on drug target modalities, such as monoclonal antibodies and gene therapies, will open new treatment possibilities.

The limitation we feel that presents the most barriers is limited multi-ancestry data. The state of diversity in the NDD research space has historically been Eurocentric, which remains the case in this study due to the limited availability of omic data from non-European participants. One of the distinguishing aspects of this study is the inclusion of multi-ancestry eQTL data in the search for generalizable drug targets. This is particularly important in an era where precision medicine and machine learning can introduce inherent bias when using reference data from solely European populations. We identified common hits, which were consistent with current understanding that there are NDD risk loci that are shared across genetic ancestries while providing insight on which gene loci and differences in expression may play a role in NDD development and treatment in non-Europeans. It is worth noting that while replication was limited at our stringent significance threshold, we were able to make some interesting observations. While we made attempts to include a limited set of multi ancestry data in the future, we would like to be able to include more multi-ancestry disease GWASs and omic data to make more meaningful insights. We recognize that we will need more multi-ancestry QTL and GWAS data for these results to be truly generalizable across different populations. We look forward to the increasing availability of non-European data with the growth of data sources such All of Us, an NIH research program focusing on inclusion of health data of marginalized populations in the United States.[70]

This report is a description of the foundation for a community-driven resource to identify and investigate future genetically derived drug targets in an open-source context. Ultimately, we are working on creating a network tool that incorporates multi-omic data, disease GWAS summary statistics, drug data, and other relevant data types to ease research such as this study, eliminating barriers to drug discovery and drug repurposing and potentially enabling precision medicine in the NDD space. Using multi-omics integration methods, deep learning techniques, and most importantly, community input to better parse and interpret the data presented by the platform, we aim to make our community resource a robust tool for NDD research.

Data and code availability

This paper analyzes existing, publicly available data. All original code has been deposited at GitHub, which can be found on the Center for Alzheimer's and Dementia GitHub (https://github.com/NIH-CARD/NDD_SMR) and Zenodo: https://doi.org/10.5281/zenodo.8425910. Results of SMR analyses can be browsed and downloaded from the Streamlit application (https://nih-card-ndd-smr-home-syboky.streamlit.app/) and csv versions are located at (https://drive.google.com/drive/folders/16lB70BgRKA8yjXuAdW3OntHIrR8gqADO).

Supplemental information

Supplemental information can be found online at https://doi.org/10.1016/j.ajhg.2023.12.006.

Acknowledgments

We would like to thank all the subjects who donated their time and biological samples to the studies that are reflected in these analyses. This research was supported in part by the Intramural Research Program of the National Institutes of Health (National Institute on Aging and National Institute of Neurological Disorders and Stroke; project numbers: 1ZIANS003154, Z01-AG000949-02, and ZIAAG000534). This study used the high-performance computational capabilities of the Biowulf Linux cluster at the National Institutes of Health (http://hpc.nih.gov). The graphical abstract was produced using Biorender.

Author contributions

M.A.N., C.X.A., D.V., H.L.L., F.F., and H.I. conceived and planned the main conceptual ideas. M.A.N., H.L.L., and F.F. supervised the project. D.V., C.X.A., and M.A.N. performed all initial data collection, wrangling, and computations. C.X.A. and C.A.W. processed and updated all experimental data, performed the analysis, and designed the figures. H.L.L., M.B.M., and M.A.N. aided in interpreting analyses results. C.X.A., M.B.M., M.A.N., and C.A.W. contributed to the drafting of the manuscript and preparation for publication. A.S., K.L., M.J.K., and S.B.C. consulted and provided critical feedback to the manuscript. All authors revised the manuscript.

Declaration of interests

C.X.A., D.V., K.L., H.L.L., F.F., and M.A.N. declare that they are consultants employed by Data Tecnica International, whose participation in this is part of a consulting agreement between the US National Institutes of Health and said company. M.A.N. is also an advisor to Neuron23, Inc. and Character Biosciences.

Received: July 19, 2023
Accepted: December 4, 2023
Published: January 4, 2024

Web resources

Biomart python package, https://github.com/sebriois/biomart
GWAS catalog, https://www.ebi.ac.uk/gwas/
metaBrain, https://www.metabrain.nl/
Open Targets Platform, https://platform.opentargets.org/
Pyensembl, https://github.com/openvax/pyensembl
SIGnaling Network Open Resource (SIGNOR), https://signor.uniroma2.it/
Streamlit, https://nih-card-ndd-smr-home-syboky.streamlit.app/
Yang Lab, https://yanglab.westlake.edu.cn/software/smr/#Overview
Yang Lab, Data Resources, https://yanglab.westlake.edu.cn/software/smr/#DataResource

References

1. Van Dyck, C.H., Swanson, C.J., Aisen, P., Bateman, R.J., Chen, C., Gee, M., Kanekiyo, M., Li, D., Reyderman, L., Cohen, S., et al. (2023). Lecanemab in Early Alzheimer's Disease. N. Engl. J. Med. *388*, 9–21.
2. Wilson, D.M., Cookson, M.R., Van Den Bosch, L., Zetterberg, H., Holtzman, D.M., and Dewachter, I. (2023). Hallmarks of neurodegenerative diseases. Cell *186*, 693–714.
3. Organization, W.H.. Dementia. https://www.who.int/news-room/fact-sheets/detail/dementia.
4. King, E.A., Davis, J.W., and Degner, J.F. (2019). Are drug targets with genetic support twice as likely to be approved? Revised estimates of the impact of genetic support for drug mechanisms on the probability of drug approval. PLoS Genet. *15*, e1008489.
5. Zhu, Z., Zhang, F., Hu, H., Bakshi, A., Robinson, M.R., Powell, J.E., Montgomery, G.W., Goddard, M.E., Wray, N.R., Visscher, P.M., and Yang, J. (2016). Integration of summary data from GWAS and eQTL studies predicts complex trait gene targets. Nat. Genet. *48*, 481–487.
6. Siletti, K., Hodge, R., Mossi Albiach, A., Hu, L., Lee, K.W., Lönnerberg, P., Bakken, T., Ding, S.-L., Clark, M., Casper, T., et al. (2022). Transcriptomic Diversity of Cell Types across the Adult Human Brain (Cold Spring Harbor Laboratory).
7. Alvarado, C.X., Weller, C.A., Johnson, N.L., Leonard, H.L., Singleton, A.B., Reed, X., Blauwendraat, C., and Nalls, M.A. (2023). Human Brain Single Nucleus Cell Type Enrichments in Neurodegenerative Diseases (Cold Spring Harbor Laboratory).
8. Bellenguez, C., Küçükali, F., Jansen, I.E., Kleineidam, L., Moreno-Grau, S., Amin, N., Naj, A.C., Campos-Martin, R., Grenier-Boley, B., Andrade, V., et al. (2022). New insights into the genetic etiology of Alzheimer's disease and related dementias. Nat. Genet. *54*, 412–436.
9. Chia, R., Sabir, M.S., Bandres-Ciga, S., Saez-Atienzar, S., Reynolds, R.H., Gustavsson, E., Walton, R.L., Ahmed, S., Viollet, C., Ding, J., et al. (2021). Genome sequencing analysis identifies new loci associated with Lewy body dementia and provides insights into its genetic architecture. Nat. Genet. *53*, 294–303.
10. Nalls, M.A., Blauwendraat, C., Vallerga, C.L., Heilbron, K., Bandres-Ciga, S., Chang, D., Tan, M., Kia, D.A., Noyce, A.J., Xue, A., et al. (2019). Identification of novel risk loci, causal insights, and heritable risk for Parkinson's disease: a meta-analysis of genome-wide association studies. Lancet Neurol. *18*, 1091–1102.
11. Nicolas, A., Kenna, K.P., Renton, A.E., Ticozzi, N., Faghri, F., Chia, R., Dominov, J.A., Kenna, B.J., Nalls, M.A., Keagle, P., et al. (2018). Genome-wide Analyses Identify KIF5A as a Novel ALS Gene. Neuron *97*, 1268–1283.e6.
12. Höglinger, G.U., Melhem, N.M., Dickson, D.W., Sleiman, P.M.A., Wang, L.-S., Klei, L., Rademakers, R., De Silva, R., Litvan, I., Riley, D.E., et al. (2011). Identification of common variants influencing risk of the tauopathy progressive supranuclear palsy. Nat. Genet. *43*, 699–705.
13. Pottier, C., Ren, Y., Perkerson, R.B., Baker, M., Jenkins, G.D., Van Blitterswijk, M., Dejesus-Hernandez, M., Van Rooij, J.G.J., Murray, M.E., Christopher, E., et al. (2019). Genome-wide analyses as part of the international FTLD-TDP whole-genome sequencing consortium reveals novel disease risk factors and increases support for immune dysfunction in FTLD. Acta Neuropathol. *137*, 879–899.
14. Kent, W.J., Sugnet, C.W., Furey, T.S., Roskin, K.M., Pringle, T.H., Zahler, A.M., Haussler, D., and David. (2002). The Human Genome Browser at UCSC. Genome Res. *12*, 996–1006.
15. Võsa, U., Claringbould, A., Westra, H.-J., Bonder, M.J., Deelen, P., Zeng, B., Kirsten, H., Saha, A., Kreuzhuber, R., Yazar, S., et al. (2021). Large-scale cis- and trans-eQTL analyses identify thousands of genetic loci and polygenic scores that regulate blood gene expression. Nat. Genet. *53*, 1300–1310.
16. De Klein, N., Tsai, E.A., Vochteloo, M., Baird, D., Huang, Y., Chen, C.-Y., Van Dam, S., Oelen, R., Deelen, P., Bakker, O.B., et al. (2023). Brain expression quantitative trait locus and network analyses reveal downstream effects and putative drivers for brain-related diseases. Nat. Genet. *55*, 377–388.
17. Zeng, B., Bendl, J., Kosoy, R., Fullard, J.F., Hoffman, G.E., and Roussos, P. (2022). Multi-ancestry eQTL meta-analysis of human brain identifies candidate causal variants for brain-related traits. Nat. Genet. *54*, 161–169.
18. GTEx Consortium, Aguet, F., Anand, S., Ardlie, K.G., Gabriel, S., Getz, G.A., Graubert, A., Hadley, K., Handsaker, R.E., Huang, K.H., et al. (2020). The GTEx Consortium atlas of genetic regulatory effects across human tissues. Science *369*, 1318–1330.
19. Wang, D., Liu, S., Warrell, J., Won, H., Shi, X., Navarro, F.C.P., Clarke, D., Gu, M., Emani, P., Yang, Y.T., et al. (2018). Comprehensive functional genomic resource and integrative model for the human brain. Science *362*, eaat8464.
20. Qi, T., Wu, Y., Zeng, J., Zhang, F., Xue, A., Jiang, L., Zhu, Z., Kemper, K., Yengo, L., Zheng, Z., et al. (2018). Identifying gene targets for brain-related traits using transcriptomic and methylomic data from blood. Nat. Commun. *9*, 2282.
21. Vaja, R., and Rana, M. (2020). Drugs and the liver. Anaesth. Intensive Care Med. *21*, 517–523.
22. McRae, A.F., Marioni, R.E., Shah, S., Yang, J., Powell, J.E., Harris, S.E., Gibson, J., Henders, A.K., Bowdler, L., Painter, J.N., et al. (2018). Identification of 55,000 Replicated DNA Methylation QTL. Sci. Rep. *8*, 17605.
23. Bryois, J., Garrett, M.E., Song, L., Safi, A., Giusti-Rodriguez, P., Johnson, G.D., Shieh, A.W., Buil, A., Fullard, J.F., Roussos, P., et al. (2018). Evaluation of chromatin accessibility in prefrontal cortex of individuals with schizophrenia. Nat. Commun. *9*, 3121.
24. Yang, C., Farias, F.H.G., Ibanez, L., Suhy, A., Sadler, B., Fernandez, M.V., Wang, F., Bradley, J.L., Eiffert, B., Bahena, J.A., et al. (2021). Genomic atlas of the proteome from brain, CSF and plasma prioritizes proteins implicated in neurological disorders. Nat. Neurosci. *24*, 1302–1312.
25. Ochoa, D., Hercules, A., Carmona, M., Suveges, D., Baker, J., Malangone, C., Lopez, I., Miranda, A., Cruz-Castillo, C.,

Fumis, L., et al. (2023). The next-generation Open Targets Platform: reimagined, redesigned, rebuilt. Nucleic Acids Res. *51*, D1353–D1359.

26. Lo Surdo, P., Iannuccelli, M., Contino, S., Castagnoli, L., Licata, L., Cesareni, G., and Perfetto, L. (2023). SIGNOR 3.0, the SIGnaling network open resource 3.0: 2022 update. Nucleic Acids Res. *51*, D631–D637.
27. Finan, C., Gaulton, A., Kruger, F.A., Lumbers, R.T., Shah, T., Engmann, J., Galver, L., Kelley, R., Karlsson, A., Santos, R., et al. (2017). The druggable genome and support for target identification and validation in drug development. Sci. Transl. Med. *9*, eaag1166.
28. Freshour, S.L., Kiwala, S., Cotto, K.C., Coffman, A.C., McMichael, J.F., Song, J.J., Griffith, M., Griffith, O.L., and Wagner, A.H. (2021). Integration of the Drug–Gene Interaction Database (DGIdb 4.0) with open crowdsource efforts. Nucleic Acids Res. *49*, D1144–D1151.
29. (2017). Openvax (PyEnsembl). https://github.com/openvax/pyensembl.
30. Briois, S.. Biomart. https://github.com/sebriois/biomart.
31. Hansen, K.D. (2016), IlluminaHumanMethylation450kanno.ilmn12.hg19: Annotation for Illumina's 450k methylation arrays. https://bioconductor.org/packages/release/data/annotation/html/IlluminaHumanMethylation450kanno.ilmn12.hg19.html.
32. 1000 Genomes Project Consortium, Auton, A., Brooks, L.D., Durbin, R.M., Garrison, E.P., Kang, H.M., Korbel, J.O., Marchini, J.L., McCarthy, S., McVean, G.A., and Abecasis, G.R. (2015). A global reference for human genetic variation. Nature *526*, 68–74.
33. Wu, Y., Zeng, J., Zhang, F., Zhu, Z., Qi, T., Zheng, Z., Lloyd-Jones, L.R., Marioni, R.E., Martin, N.G., Montgomery, G.W., et al. (2018). Integrative analysis of omics summary data reveals putative mechanisms underlying complex traits. Nat. Commun. *9*, 918.
34. Seo, J., and Park, M. (2020). Molecular crosstalk between cancer and neurodegenerative diseases. Cell. Mol. Life Sci. *77*, 2659–2680.
35. Tobin, J.E., Latourelle, J.C., Lew, M.F., Klein, C., Suchowersky, O., Shill, H.A., Golbe, L.I., Mark, M.H., Growdon, J.H., Wooten, G.F., et al. (2008). Haplotypes and gene expression implicate the MAPT region for Parkinson disease: The GenePD Study. Neurology *71*, 28–34.
36. Zhou, F., and Wang, D. (2017). The associations between the MAPT polymorphisms and Alzheimer's disease risk: a meta-analysis. Oncotarget *8*, 43506–43520.
37. Majounie, E., Cross, W., Newsway, V., Dillman, A., Vandrovcova, J., Morris, C.M., Nalls, M.A., Ferrucci, L., Owen, M.J., O'Donovan, M.C., et al. (2013). Variation in tau isoform expression in different brain regions and disease states. Neurobiol. Aging *34*, 1922.e7–1922.e12.
38. Lin, M.K., and Farrer, M.J. (2014). Genetics and genomics of Parkinson's disease. Genome Med. *6*, 48.
39. Jun, G., Ibrahim-Verbaas, C.A., Vronskaya, M., Lambert, J.C., Chung, J., Naj, A.C., Kunkle, B.W., Wang, L.S., Bis, J.C., Bellenguez, C., et al. (2016). A novel Alzheimer disease locus located near the gene encoding tau protein. Mol. Psychiatr. *21*, 108–117.
40. Patel, D., Zhang, X., Farrell, J.J., Chung, J., Stein, T.D., Lunetta, K.L., and Farrer, L.A. (2021). Cell-type-specific expression quantitative trait loci associated with Alzheimer disease in blood and brain tissue. Transl. Psychiatry *11*, 250.
41. Futch, H.S., Croft, C.L., Truong, V.Q., Krause, E.G., and Golde, T.E. (2017). Targeting psychologic stress signaling pathways in Alzheimer's disease. Mol. Neurodegener. *12*, 49.
42. Zhao, B., Shan, Y., Yang, Y., Yu, Z., Li, T., Wang, X., Luo, T., Zhu, Z., Sullivan, P., Zhao, H., et al. (2021). Transcriptome-wide association analysis of brain structures yields insights into pleiotropy with complex neuropsychiatric traits. Nat. Commun. *12*, 2878.
43. Madrid, L., Labrador, S.C., González-Pérez, A., Sáez, M.E., and The Alzheimer's Disease Neuroimaging Initiative Adni. (2021). Integrated Genomic, Transcriptomic and Proteomic Analysis for Identifying Markers of Alzheimer's Disease. Diagnostics *11*, 2303.
44. Sheikh, B.N., Guhathakurta, S., and Akhtar, A. (2019). The non-specific lethal NSL complex at the crossroads of transcriptional control and cellular homeostasis. EMBO Rep. *20*, e47630.
45. Linda, K., Lewerissa, E.I., Verboven, A.H.A., Gabriele, M., Frega, M., Klein Gunnewiek, T.M., Devilee, L., Ulferts, E., Hommersom, M., Oudakker, A., et al. (2022). Imbalanced autophagy causes synaptic deficits in a human model for neurodevelopmental disorders. Autophagy *18*, 423–442.
46. Button, R.W., Roberts, S.L., Willis, T.L., Hanemann, C.O., and Luo, S. (2017). Accumulation of autophagosomes confers cytotoxicity. J. Biol. Chem. *292*, 13599–13614.
47. Son, S.M., Park, S.J., Fernandez-Estevez, M., and Rubinsztein, D.C. (2021). Autophagy regulation by acetylation—implications for neurodegenerative diseases. Exp. Mol. Med. *53*, 30–41.
48. Lingling, D., Miaomiao, Q., Yili, L., Hongyun, H., and Yihao, D. (2022). Attenuation of histone H4 lysine 16 acetylation (H4K16ac) elicits a neuroprotection against ischemic stroke by alleviating the autophagic/lysosomal dysfunction in neurons at the penumbra. Brain Res. Bull. *184*, 24–33.
49. Füllgrabe, J., Lynch-Day, M.A., Heldring, N., Li, W., Struijk, R.B., Ma, Q., Hermanson, O., Rosenfeld, M.G., Klionsky, D.J., and Joseph, B. (2013). The histone H4 lysine 16 acetyltransferase hMOF regulates the outcome of autophagy. Nature *500*, 468–471.
50. Wiesel-Motiuk, N., and Assaraf, Y.G. (2020). The key roles of the lysine acetyltransferases KAT6A and KAT6B in physiology and pathology. Drug Resist. Updates *53*, 100729.
51. Wang, C., Telpoukhovskaia, M.A., Bahr, B.A., Chen, X., and Gan, L. (2018). Endo-lysosomal dysfunction: a converging mechanism in neurodegenerative diseases. Curr. Opin. Neurobiol. *48*, 52–58.
52. Kragh, C.L., Ubhi, K., Wyss-Coray, T., and Masliah, E. (2012). Autophagy in Dementias. Brain Pathol. *22*, 99–109.
53. McEwan, D.G., Popovic, D., Gubas, A., Terawaki, S., Suzuki, H., Stadel, D., Coxon, F.P., Miranda de Stegmann, D., Bhogaraju, S., Maddi, K., et al. (2015). PLEKHM1 Regulates Autophagosome-Lysosome Fusion through HOPS Complex and LC3/GABARAP Proteins. Mol. Cell *57*, 39–54.
54. Lichtenthaler, S.F., Lemberg, M.K., and Fluhrer, R. (2018). Proteolytic ectodomain shedding of membrane proteins in mammals—hardware, concepts, and recent developments. EMBO J. *37*, e99456.
55. Chen, F., Chen, H., Jia, Y., Lu, H., Tan, Q., and Zhou, X. (2020). miR-149-5p inhibition reduces Alzheimer's disease β-amyloid generation in 293/APPsw cells by upregulating H4K16ac via KAT8. Exp. Ther. Med. *20*, 1-1.

56. Erekat, N.S. (2018). Apoptosis and its Role in Parkinson's Disease (Codon Publications), pp. 65–82.
57. Goel, P., Chakrabarti, S., Goel, K., Bhutani, K., Chopra, T., and Bali, S. (2022). Neuronal cell death mechanisms in Alzheimer's disease: An insight. Front. Mol. Neurosci. *15*, 937133.
58. Van Den Bosch, T., Leus, N.G.J., Wapenaar, H., Boichenko, A., Hermans, J., Bischoff, R., Haisma, H.J., and Dekker, F.J. (2017). A 6-alkylsalicylate histone acetyltransferase inhibitor inhibits histone acetylation and pro-inflammatory gene expression in murine precision-cut lung slices. Pulm. Pharmacol. Ther. *44*, 88–95.
59. Dekker, F.J., van den Bosch, T., and Martin, N.I. (2014). Small molecule inhibitors of histone acetyltransferases and deacetylases are potential drugs for inflammatory diseases. Drug Discov. Today *19*, 654–660.
60. Baird, D.A., Liu, J.Z., Zheng, J., Sieberts, S.K., Perumal, T., Elsworth, B., Richardson, T.G., Chen, C.-Y., Carrasquillo, M.M., Allen, M., et al. (2021). Identifying drug targets for neurological and psychiatric disease via genetics and the brain transcriptome. PLoS Genet. *17*, e1009224.
61. Li, Y.I., Wong, G., Humphrey, J., and Raj, T. (2019). Prioritizing Parkinson's disease genes using population-scale transcriptomic data. Nat. Commun. *10*, 994.
62. Diaz-Ortiz, M.E., Seo, Y., Posavi, M., Carceles Cordon, M., Clark, E., Jain, N., Charan, R., Gallagher, M.D., Unger, T.L., Amari, N., et al. (2022). GPNMB confers risk for Parkinson's disease through interaction with α-synuclein. Science *377*, eabk0637.
63. Nalls, M.A., Pankratz, N., Lill, C.M., Do, C.B., Hernandez, D.G., Saad, M., Destefano, A.L., Kara, E., Bras, J., Sharma, M., et al. (2014). Large-scale meta-analysis of genome-wide association data identifies six new risk loci for Parkinson's disease. Nat. Genet. *46*, 989–993.
64. Pajares, M., A, I.R., Manda, G., Boscá, L., and Cuadrado, A. (2020). Inflammation in Parkinson's Disease: Mechanisms and Therapeutic Implications. Cells *9*.
65. Marogianni, C., Sokratous, M., Dardiotis, E., Hadjigeorgiou, G.M., Bogdanos, D., and Xiromerisiou, G. (2020). Neurodegeneration and Inflammation—An Interesting Interplay in Parkinson's Disease. Int. J. Mol. Sci. *21*, 8421.
66. Matthews, D.C., Mao, X., Dowd, K., Tsakanikas, D., Jiang, C.S., Meuser, C., Andrews, R.D., Lukic, A.S., Lee, J., Hampilos, N., et al. (2021). Riluzole, a glutamate modulator, slows cerebral glucose metabolism decline in patients with Alzheimer's disease. Brain *144*, 3742–3755.
67. Wu, H.-C., Chen, C.-M., Chen, Y.-C., Fung, H.-C., Chang, K.-H., and Wu, Y.-R. (2018). DLG2, but not TMEM229B, GPNMB, and ITGA8 polymorphism, is associated with Parkinson's disease in a Taiwanese population. Neurobiol. Aging *64*, 158.e1–158.e6.
68. Xu, Y., Chen, Y., Ou, R., Wei, Q.-Q., Cao, B., Chen, K., and Shang, H.-F. (2016). No association of GPNMB rs156429 polymorphism with Parkinson's disease, amyotrophic lateral sclerosis and multiple system atrophy in Chinese population. Neurosci. Lett. *622*, 113–117.
69. Rizig, M., Bandres-Ciga, S., Makarious, M.B., Ojo, O.O., Crea, P.W., Abiodun, O.V., Levine, K.S., Abubakar, S.A., Achoru, C.O., Vitale, D., et al. (2023). Identification of genetic risk loci and causal insights associated with Parkinson's disease in African and African admixed populations: a genome-wide association study. Lancet Neurol. *22*, 1015–1025.
70. Program, N.I.o.H.A.o.U.R.. All of Us Research Hub. https://www.researchallofus.org/.

The American Journal of Human Genetics, Volume *111*

Supplemental information

omicSynth: An open multi-omic community resource for identifying druggable targets across neurodegenerative diseases

Chelsea X. Alvarado, Mary B. Makarious, Cory A. Weller, Dan Vitale, Mathew J. Koretsky, Sara Bandres-Ciga, Hirotaka Iwaki, Kristin Levine, Andrew Singleton, Faraz Faghri, Mike A. Nalls, and Hampton L. Leonard

Supplementary Tables Legend

Table S1: Summary of SMR data mining across diseases. This table provides a summary of the number of unique genes, genes found to be significant in liver eQTL tissue, eQTL specific genes, number of genes replicated in multi ancestry eQTL data, total number of gene found to be therapeutic/druggable, the percentage of therapeutic genes, total number of non-therapeutic/non-druggable genes, and the percentage of non-therapeutic genes for each diseases. This data is provided at 4 levels, an overall summary of all tested genes and three significance thresholds: $p_{SMR\_multi} < 0.05$ & $p_{HEIDI} > 0.01$, $p_{SMR\_multi} < 2.95 \times 10^{-6}$ (testing all protein coding genes) & $p_{HEIDI} > 0.01$, and $p_{SMR\_multi} < 1.58 \times 10^{-8}$ (testing all protein coding genes across all omics) & $p_{HEIDI} > 0.01$.

Table S2:All unfiltered SMR association summary statistics. Table provides a link to data files for download.

Table S3: Extended data for candidate genes for multiple neurodegenerative diseases (Table 1). This table provides extended data for genes with functional inferences passing multiple test correction at a multi-SNP SMR $P < 2.95 \times 10^{-6}$ for multiple neurodegenerative diseases. We provide details for the most significant *-omic association detected as well as the most significant single SNP in that set of gene level associations. Additionally, we provide all the omics and diseases in which a given gene has significant associations.

Table S4: Summary statistics for all significant gene associations. This table provides summary statistics and SMR results for candidate genes with associations passing multiple test correction at a multi-SNP SMR P < 2.95E-06. Columns ending with GWAS and QTL provide values for the tested probe from the source QTL and GWAS summary statistics used in the SMR analyses.

Table S5: Extended Summaries of candidate genes in three neurodegenerative diseases. This table provides extended data on candidate genes passing multiple test correction at a multi-SNP SMRP < 2.95E-06 for three neurodegenerative diseases. We provide summary statistics for each significant association.

Table S6: Extended Summaries of candidate genes in two neurodegenerative diseases. This table provides extended data on candidate genes passing multiple test correction at a multi-SNP SMRP < 2.95E-06 for two neurodegenerative diseases. We provide summary statistics for each significant association.

Table S7: Summary statistics for candidate genes replicated in multi ancestry data. This table provides summary statistics for all replicated candidate genes’ significant associations across all *-omics.

Table S8: Summary statistics for all novel class genes. This table provides summary statistics of candidate genes classified as *novel,* meaning the gene is considered druggable but does not have NDD specific therapeutics that target the gene. Significant associations for each gene are provided.

Table S9: Summary statistics for all known class genes. This table provides summary statistics of candidate genes classified as *known,* meaning that the gene has therapeutics that are approved for use in at least one NDD. We provide all significant associations for each *known* gene.

Table S10: Summaries of the novel target class of genes including network members. This table lists all candidate genes that fall into the novel tier in our druggable gene classification scheme. For each gene we provide a summary of how many omics a gene has significant associations in and in which diseases. We identify companion genes for each gene using the Signor database and in bold are genes that have been found to have significant associations in our SMR results (multi-SNP SMRP < 2.95E-06).

Table S11: Summary statistics for all difficult class genes. This table provides summary statistics of candidate genes classified as *difficult,* meaning the gene is not currently considered druggable and no therapeutics target the gene.

Table S12: Summaries of the difficult target class of genes including network members. This table lists all candidate genes that fall into the difficult tier in our druggable gene classification scheme. For each gene we provide a summary of how many omics a gene has significant associations in and in which diseases. We identify companion genes for each gene using the Signor database and in bold are genes that have been found to have significant associations in our SMR results (multi-SNP SMRP < 2.95E-06). We provide additional information regarding potential liver toxicity issues by cross referencing companion genes against significant associations in the liver omic.

Table S13: Mean expression percentile ranks in single cell data for multi-NDD gene targets. This table provides calculated mean expression rank values using snRNA-seq data from Silleti et al. (2022). Mean values for each gene-cell type were obtained by using scRNA-seq values for all cell type specific individual cells for a specific gene.

Table S14: Median expression percentile ranks in single cell data for multi-NDD gene targets. This table provides calculated median expression rank values using snRNA-seq data from Silleti et al. (2022). Median values for each gene-cell type were obtained by using scRNA-seq values for all cell type specific individual cells for a specific gene.

Table S15: Comparison of GPNMB across multiple genetic ancestries. This table provides basic summary statistics of our GPNMB significant associations in comparison to other GPNMB summary statistics. We summarize data in African American/African admixed, Chinese, and Taiwanese genetic ancestries to confirm that our multi ancestry association direction was consistent with previous findings in other non-European ancestries.

Supplementary Figure(s)

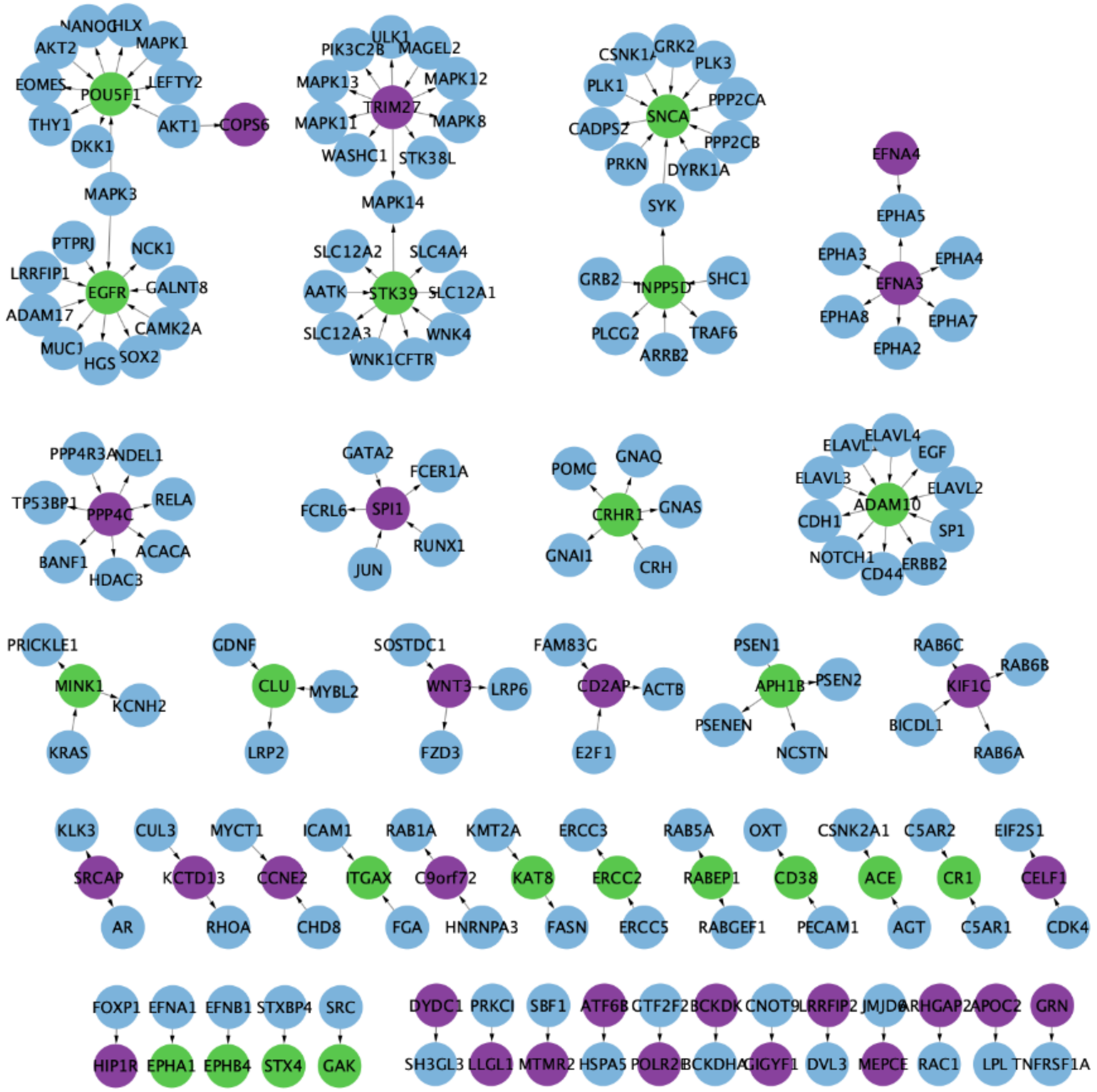

Figure S1: Network Visualization of *Novel* and *Difficult* genes companion genes Graph network visualization of both *novel* (green nodes) and *difficult* (purple nodes) genes and their SIGNOR curated partners (blue nodes). The direction of connecting arrows indicates interaction from regulator to target.

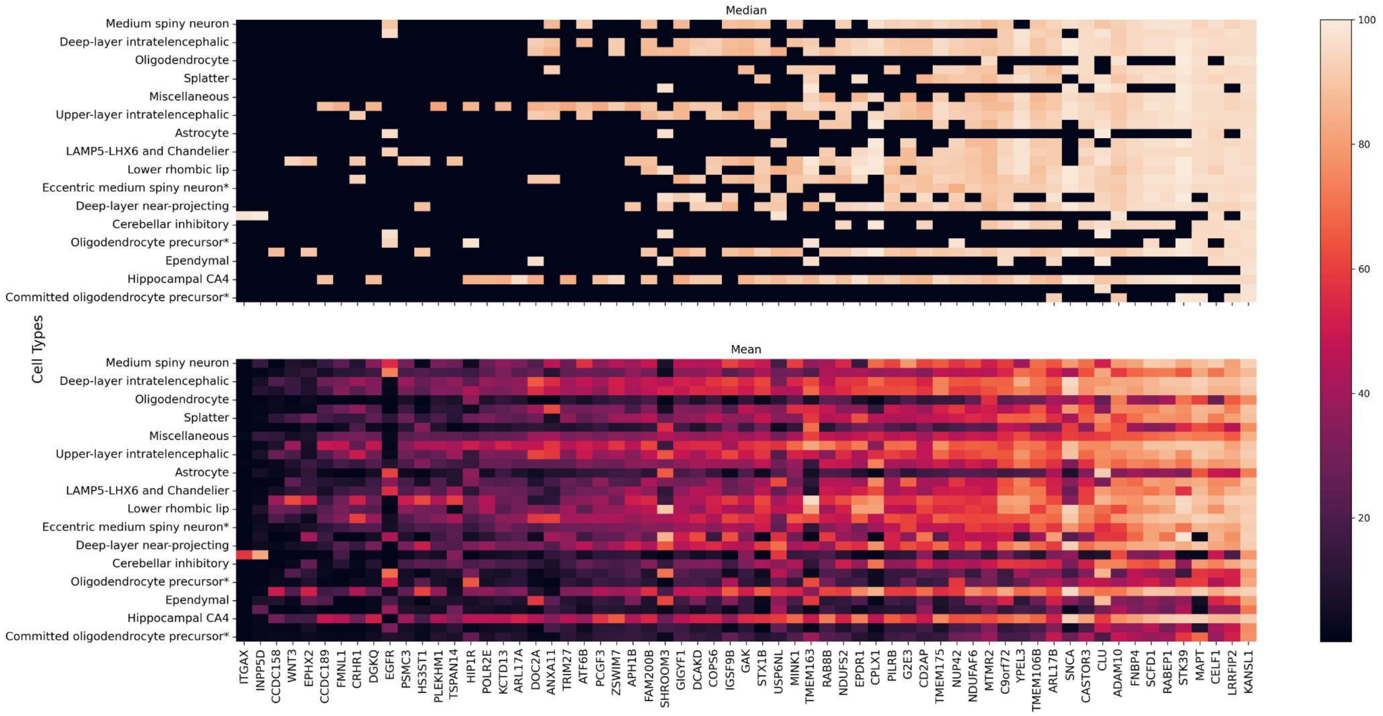

Figure S2: scRNA-seq expression for significant genes (p_{SMR_multi} < 2.95×10-6 and p_{HEIDI} > 0.01). Heatmaps were used to illustrate both the median (top plot) and mean (bottom plot) expression percentile rank for each gene-celltype combination using significant genes and disease relevant cell types.

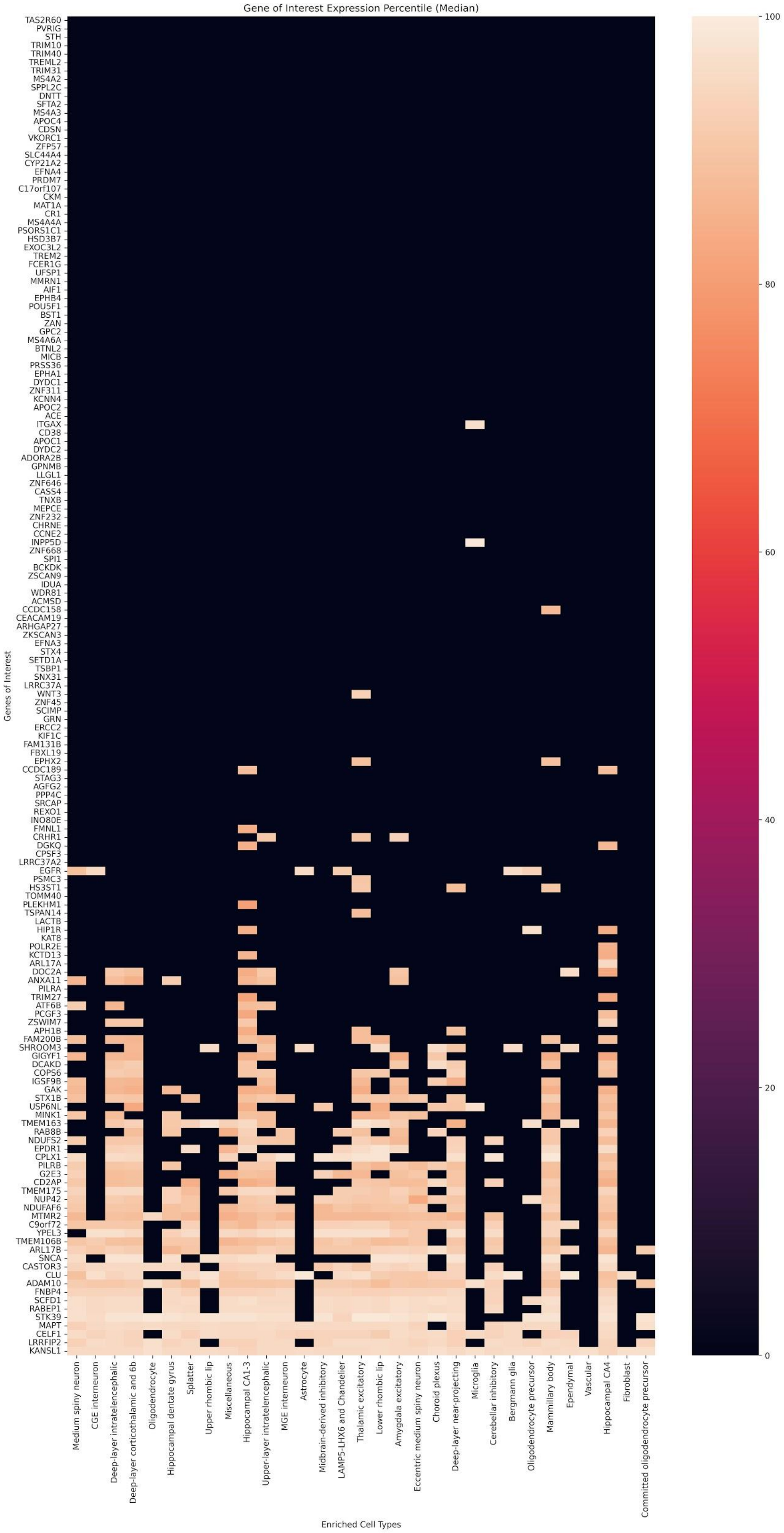

Figure S3: Median scRNA-seq expression for all significant genes. Heatmap illustration of the median expression percentile rank value for each gene-celltype combination.

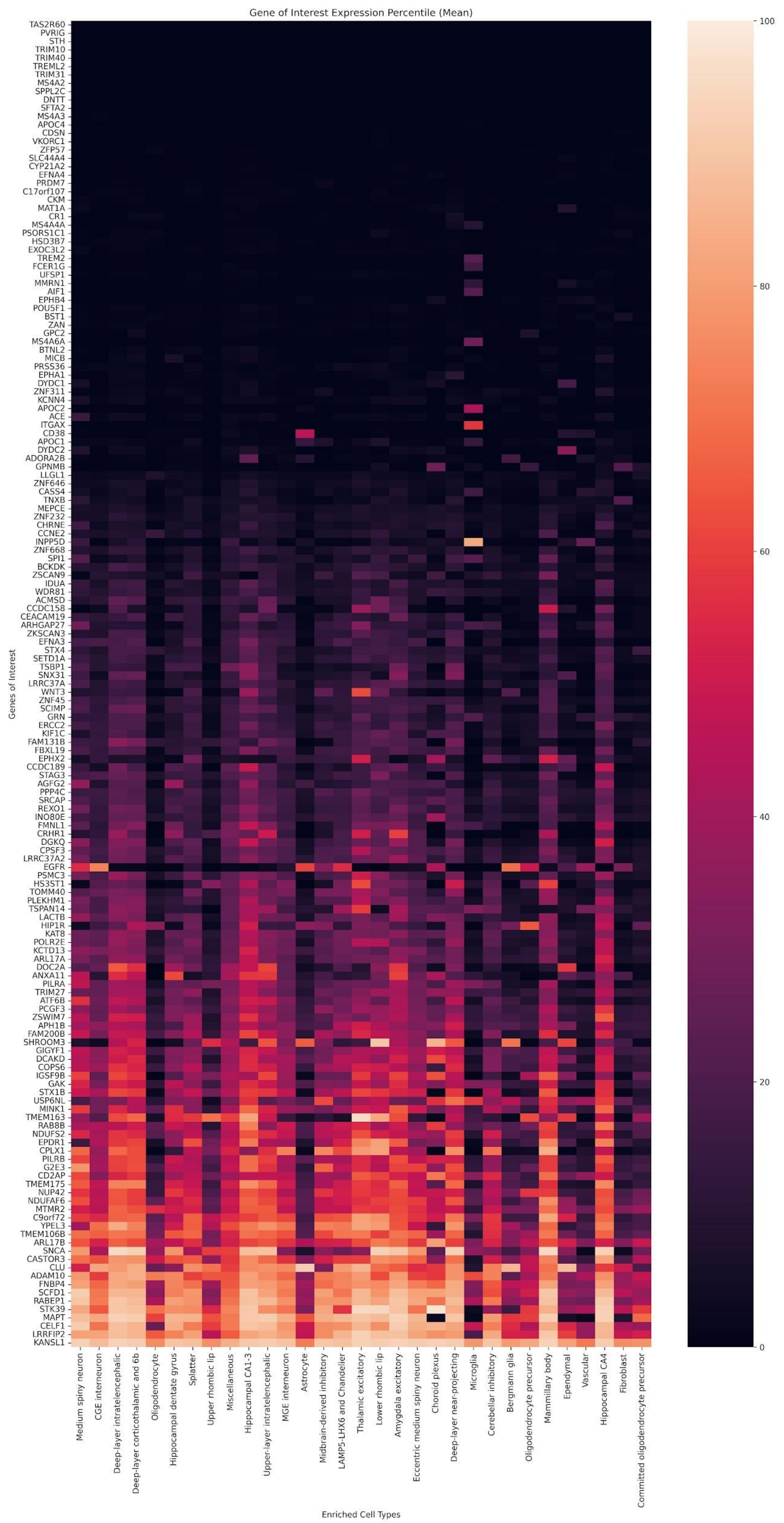

Figure S4: Mean scRNA-seq expression for all significant genes. Heatmap illustration of the mean expression percentile rank value for each gene-celltype combination.

S1 - SMR Result Summary

Disease	Total Genes (Unique)	Liver Genes	Total eQTL Genes (non multi ancestry)	Replicated in Multi Ancestry	Total Therapeutic Genes	% Therapeutic	Total Non-therapeutic Genes	% Non-therapeutic
**All Tested Genes (Protein Coding)**								
AD	16833	1597	15112	8404	3,562	21.2%	13,271	78.8%
ALS	16875	1610	15163	8408	3,565	21.1%	13,310	78.9%
FTLD	16788	1537	15038	8394	3,551	21.2%	13,237	78.8%
LBD	16797	1540	15069	8388	3,554	21.2%	13,243	78.8%
PD	16872	1596	15159	8407	3,566	21.1%	13,306	78.9%
PSP	16042	1033	13839	8073	3,420	21.3%	12,622	78.7%
**Significance p_SMR_multi < 0.05 & p_HEIDI > 0.01**								
**Disease**	**Total Significant Genes (Unique)**		**Total eQTL Genes (non multi ancestry)**	**Replicated in Multi Ancestry**		**% Therapeutic + Sig**		**% Non-therapeutic + Sig**
AD	8	175	3189	2079	1,142	14275.0%	3,806	47575.0%
ALS	3188	83	1857	1260	715	22.4%	2,473	77.6%
FTLD	2318	78	1243	810	542	23.4%	1,776	76.6%
LBD	2530	82	1384	900	580	22.9%	1,950	77.1%
PD	3592	108	2161	1434	811	22.6%	2,781	77.4%
PSP	2275	30	1270	842	574	25.2%	1,701	74.8%
**Significance p_SMR_multi < 2.95E-06 (testing all protein coding genes) & p_HEIDI > 0.01**								
AD	116	2	68	7	31	26.7%	85	73.3%
ALS	3	0	3	0	0	0.0%	3	100.0%
FTLD	0	0	0	0	0	0.0%	0	0.0%
LBD	5	0	1	0	1	20.0%	4	80.0%
PD	46	3	33	5	15	32.6%	31	67.4%
PSP	9	0	5	3	2	22.2%	7	77.8%
**Significance p_SMR_multi < 1.58E-08 (testing all protein coding genes across all omics) & p_HEIDI > 0.01**								
AD	47	1	19	19	14	29.8%	33	70.2%
ALS	1	0	0	0	0	0.0%	1	100.0%
FTLD	0	0	0	0	0	0.0%	0	0.0%
LBD	2	0	0	0	1	50.0%	1	50.0%
PD	24	1	14	14	8	33.3%	16	66.7%
PSP	8	0	5	5	2	25.0%	6	75.0%

All protein coding associations can be download from this link: https://drive.google.com/file/d/1I70F2UcEoKEFJYRM4LYtbsr61QP2VjAV/view?usp=share_link (~200mb)

Gene	Omic	Disease	topRSID	beta, SMR	SE, SMR	p, SMR	p, multi-SNP SMR	p, HEIDI	Diseases	Omics
									AD	Cerebellum eQTL, Cortex eQTL
ARL17B	Spinal cord eQTL	PD	rs199451	-0.202836	0.0332609	1.07E-09	6.42E-09	0.01223556	PD	Spinalcord eQTL
									PD	Spinalcord eQTL
KAT8	Tibial Nerve eQTL	PD	rs9936329	-0.428193	0.0892292	1.60E-06	3.76E-08	0.06971106	AD	Cerebellum eQTL, Whole Brain meta-analysis mQTL, Cerebellar Hemisphere eQTL, Cortex eQTL, Tibial Nerve eQTL, Skeletal Muscle eQTL, Hypothalamus eQTL, Whole Brain eQTL, Cerebellum eQTL
									PD	Hippocampus eQTL, Cortex eQTL, Frontal Cortex BA9 eQTL, Prefrontal Cortex eQTL, Caudate Basal Ganglia eQTL, Skeletal Muscle eQTL, Hippocampus eQTL, Multi Ancestry Whole Brain Meta-analysis eQTL, Hypothalamus eQTL, Liver eQTL, Anterior Cingulate Cortex BA24 eQTL, Putamen Basal Ganglia eQTL, Amygdala eQTL, Whole Brain eQTL, Cerebellum eQTL, Nucleus Accumbens Basal Ganglia
LRRC37A2	Multi Ancestry Whole Brain Meta-analysis eQTL	PD	rs2532329	-0.22263	0.0255896	3.32E-18	1.22E-15	0.4841153	AD	Basal Ganglia eQTL, Spinalcord eQTL, Frontal Cortex BA9 eQTL, Hippocampus eQTL, Multi Ancestry Whole Brain Meta-analysis eQTL, Substantia nigra eQTL, Liver eQTL, Putamen Basal Ganglia eQTL, Amygdala eQTL
									PD	Whole Brain meta-analysis mQTL, Whole Blood mQTL, Cortex eQTL, Multi Ancestry Whole Brain Meta-analysis eQTL
									PSP	Spinalcord eQTL, Whole Brain meta-analysis mQTL, Whole Blood mQTL
KANSL1	Whole Brain meta-analysis mQTL	PD	rs1966345	0.15927	0.0174497	7.02E-20	2.28E-19	0.06522781	AD	Spinalcord eQTL, Whole Blood mQTL, Anterior Cingulate Cortex BA24 eQTL
									PSP	Multi Ancestry Whole Brain Meta-analysis eQTL, Hypothalamus eQTL
									PD	Cerebellar Hemisphere eQTL, Cortex eQTL, Caudate Basal Ganglia eQTL, Hippocampus eQTL, Multi Ancestry Whole Brain Meta-analysis eQTL, Hypothalamus eQTL, Anterior Cingulate Cortex BA24 eQTL, Putamen Basal Ganglia eQTL, Cerebellum eQTL, Nucleus Accumbens Basal Ganglia
ARL17A	Multi Ancestry Whole Brain Meta-analysis eQTL	PD	rs58879558	-0.337169	0.0385816	2.35E-18	3.03E-15	0.1614406	AD	Spinalcord eQTL, Amygdala eQTL
									AD	Whole Brain meta-analysis mQTL, Cerebellar Hemisphere eQTL, Cortex eQTL, Multi Ancestry Whole Brain Meta-analysis eQTL, Whole Brain eQTL
PRSS36	Whole Brain meta-analysis mQTL	AD	rs55667375	0.0928108	0.0167849	3.21E-08	6.46E-07	0.01582905	PD	Whole Brain meta-analysis mQTL
									AD	Whole Brain meta-analysis mQTL
									PD	Whole Brain meta-analysis mQTL, Whole Blood mQTL
MAPT	Whole Brain meta-analysis mQTL	PSP	rs1981997	1.14017	0.0815642	2.10E-44	2.10E-44	0.01425494	PSP	Whole Brain meta-analysis mQTL, Whole Blood mQTL
									LBD	
IDUA	Whole Brain meta-analysis mQTL	PD	rs6599388	-0.134337	0.0147854	1.03E-19	1.87E-12	0.01914885	PD	Whole Brain meta-analysis mQTL
									PD	
TMEM175	Whole Blood mQTL	PD	rs11248057	0.495391	0.0721195	6.46E-12	4.33E-11	0.6479261	LBD	Whole Blood mQTL
									PD	Whole Blood mQTL, Multi Ancestry Whole Brain Meta-analysis eQTL
									AD	Whole Brain meta-analysis mQTL, Whole Blood mQTL, Caudate Basal Ganglia eQTL, Nucleus Accumbens Basal Ganglia
ARHGAP27	Whole Blood mQTL	PSP	rs11012	4.15654	0.543286	2.00E-14	1.36E-13	0.07969932	PSP	Whole Blood mQTL, Whole Blood eQTL eQTLgen
									PD	Whole Blood mQTL
									PSP	Whole Brain meta-analysis mQTL, Whole Blood mQTL, Cortex eQTL, Skeletal Muscle eQTL
CRHR1	Whole Brain meta-analysis mQTL	PSP	rs12373139	1.79071	0.188188	1.81E-21	5.15E-18	0.2191273	AD	Whole Brain meta-analysis mQTL, Whole Blood mQTL
									PSP	Multi Ancestry Whole Brain Meta-analysis eQTL
FMNL1	Whole Blood mQTL	PSP	rs17630064	0.247221	0.0717373	0.0005685207	7.24E-08	0.06396753	AD	Whole Blood mQTL
									PD	Cortex eQTL, Frontal Cortex BA9 eQTL, Prefrontal Cortex eQTL, Cortex eQTL, Caudate Basal Ganglia eQTL, Skeletal Muscle eQTL, Anterior Cingulate Cortex BA24 eQTL, Putamen Basal Ganglia eQTL, Whole Brain eQTL
PLEKHM1	Skeletal Muscle eQTL	PD	rs12947718	-0.667265	0.0974424	7.50E-12	7.76E-12	0.03535971	PSP	Anterior Cingulate Cortex BA24 eQTL
									PD	Cortex eQTL metaBrain, Skeletal Muscle eQTL
WNT3	Cortex eQTL metaBrain	PD	rs9904865	-0.062473	0.0240469	0.009377936	1.37E-08	0.09647706	AD	Tibial Nerve eQTL
									PD	Cerebellum eQTL
SPPL2C	Prefrontal Cortex eQTL	PD	rs17577369	1.04066	0.182771	1.24E-08	1.30E-07	0.01842581	AD	Prefrontal Cortex eQTL

Gene	Disease	Omic	probeID	ProbeChr	Probe_bp	topRSID	topSNP_chr	topSNP_bp	A1	A2	beta, SMR	SE, SMR	p, SMR	p, multi-SNP SMR	p, HEIDI
		Cerebellum eQTL	ENSG00000228696	17	46361797	rs7225002	17	44189067	A	G	-0.0626033	0.0105364	2.82E-09	4.20E-07	0.5937533
	AD	Cortex eQTL metaBrain	ENSG00000228696	17	46361797	rs538628	17	44787313	G	C	-0.0537127	0.00920295	5.33E-09	6.60E-08	0.6738135
ARL17B	PD	Spinalcord eQTL	ENSG00000228696	17	46361797	rs199451	17	44801784	G	A	-0.202836	0.0332609	1.07E-09	6.42E-09	0.01223556
	AD	Multi Ancestry Whole Brain Meta-analysis eQTL	ENSG00000184922	17	43298811	rs62063276	17	44036408	G	T	-0.134405	0.0271688	7.53E-07	2.21E-06	0.6697149
FMNL1	PSP	Whole Blood mQTL	cg19481029	17	43314586	rs17630064	17	43289122	G	A	0.247221	0.0717373	5.69E-04	7.24E-08	0.06396753
	LBD	Whole Brain meta-analysis mQTL	cg08160350	4	996052	rs6599388	4	939087	C	T	-0.165404	0.030336	4.97E-08	2.81E-06	0.08410463
		Cortex eQTL metaBrain	ENSG00000127415	4	986997	rs73211813	4	975238	C	T	-0.435648	0.0846761	2.68E-07	2.39E-08	0.03864672
		Whole Blood eQTL GTEx	ENSG00000127415	4	980785	rs11248061	4	980896	A	C	-0.702144	0.152039	3.87E-06	4.08E-07	0.02435755
			cg00247629	4	995263	rs62294519	4	988619	C	T	-0.487929	0.122202	6.53E-05	2.00E-08	0.05213469
		Whole Blood mQTL	cg27494429	4	997540	rs11248060	4	964359	T	C	-0.486581	0.107127	5.57E-06	1.47E-07	0.07626291
			cg01962146	4	995931	rs1051613	4	951179	A	G	-0.165566	0.0343796	1.47E-06	8.18E-11	0.04819888
			cg08160350	4	996052	rs6599388	4	939087	C	T	-0.134337	0.0147854	1.03E-19	1.87E-12	0.01914885
			cg00187933	4	996785	rs11248061	4	980896	A	C	-0.235183	0.0490756	1.65E-06	3.55E-09	0.08509635
			cg15769764	4	996790	rs11248061	4	980896	A	C	-0.270404	0.0587199	4.12E-06	1.67E-07	0.09144947
IDUA	PD	Whole Brain meta-analysis mQTL	cg08332990	4	997351	rs11248061	4	980896	A	C	-0.248079	0.0525305	2.33E-06	1.16E-08	0.1446274
		Whole Blood eQTL eQTLgen	ENSG00000103510	16	31134894	rs1060506	16	31133449	T	C	-0.216715	0.0379747	1.15E-08	1.78E-07	0.1463961
		Whole Blood mQTL	cg02220965	16	31128310	rs1978487	16	31129942	C	T	0.0603758	0.0130409	3.66E-06	2.55E-07	0.06856892
		Whole Brain eQTL	ENSG00000103510	16	31134894	rs12597511	16	31145219	T	C	-0.079204	0.0178323	8.93E-06	1.45E-06	0.2647209
	AD	Whole Brain meta-analysis mQTL	cg02220965	16	31128310	rs4889619	16	31128615	T	C	0.0713321	0.0162194	1.09E-05	2.80E-07	0.1321537
		Cerebellar Hemisphere eQTL	ENSG00000103510	16	31127075	rs2855475	16	31147548	A	G	-0.233509	0.051988	7.07E-06	3.28E-07	0.02523191
		Cerebellum eQTL	ENSG00000103510	16	31114489	rs1549293	16	31141993	C	T	-0.17521	0.039031	7.16E-06	1.21E-06	0.04107506
		Cerebellum eQTL	ENSG00000103510	16	31127075	rs9972727	16	31149142	G	A	-0.176591	0.0390447	6.10E-06	1.04E-06	0.01264411
		Cortex eQTL GTEx	ENSG00000103510	16	31127075	rs1060506	16	31133449	T	C	-0.189104	0.0477015	7.36E-05	6.68E-07	0.03828886
		Hypothalamus eQTL	ENSG00000103510	16	31127075	rs61162043	16	31114234	G	A	-0.245687	0.0612799	6.09E-05	1.81E-06	0.2130376
		Skeletal Muscle eQTL	ENSG00000103510	16	31127075	rs9925964	16	31129895	G	A	-0.390776	0.0836696	3.01E-06	1.57E-07	0.0538749
		Tibial Nerve eQTL	ENSG00000103510	16	31127075	rs9936329	16	31140799	T	G	-0.428193	0.0892292	1.60E-06	3.76E-08	0.06971106
		Whole Brain eQTL	ENSG00000103510	16	31134894	rs12597511	16	31145219	T	C	-0.184809	0.0378713	1.06E-06	1.84E-07	0.04586272
KAT8	PD	Whole Brain meta-analysis mQTL	cg02220965	16	31128310	rs4889619	16	31128615	T	C	0.170253	0.0349027	1.07E-06	2.53E-06	0.5369356
		Amygdala eQTL	ENSG00000238083	17	44588877	rs2942166	17	43715427	C	T	-0.0465837	0.00988142	2.43E-06	2.54E-06	0.3929416
		Anterior Cingulate Cortex BA24 eQTL	ENSG00000238083	17	44588877	rs56971664	17	43918613	C	T	-0.0439728	0.00888721	7.50E-07	1.03E-06	0.6869415
		Caudate Basal Ganglia eQTL	ENSG00000238083	17	44588877	rs8073146	17	43893751	G	A	-0.0431091	0.00859267	5.25E-07	2.96E-06	0.4893544
		Cerebellum eQTL	ENSG00000238083	17	44588877	rs17564020	17	43991781	T	G	-0.0455583	0.0087789	2.11E-07	1.64E-06	0.8570875
		Cortex eQTL metaBrain	ENSG00000238083	17	46511511	rs2696466	17	44289832	A	G	-0.0586461	0.00950285	6.77E-10	3.80E-08	0.8992636
		Frontal Cortex BA9 eQTL	ENSG00000238083	17	44588877	rs4074462	17	43855228	T	G	-0.0430712	0.00872755	8.01E-07	1.50E-06	0.817564
		Hippocampus eQTL eQTLgen	ENSG00000238083	17	46511511	rs2696466	17	44289832	G	A	-0.0644059	0.0125498	2.87E-07	2.88E-06	0.5139333
		Hippocampus eQTL GTEx	ENSG00000238083	17	44588877	rs2942166	17	43715427	C	T	-0.0431814	0.00898806	1.55E-06	2.25E-06	0.3720312
		Hypothalamus eQTL	ENSG00000238083	17	44588877	rs8073146	17	43893751	G	A	-0.0404116	0.00803609	4.94E-07	1.59E-06	0.4894522
		Liver eQTL	ENSG00000238083	17	44588877	rs58879558	17	44095467	C	T	-0.0659929	0.0128493	2.81E-07	1.90E-06	0.1531949
		Multi Ancestry Whole Brain Meta-analysis eQTL	ENSG00000238083	17	44588877	rs2532329	17	44350090	A	G	-0.04872	0.00902299	6.68E-08	4.57E-08	0.9418131
		Nucleus Accumbens Basal Ganglia	ENSG00000238083	17	44588877	rs413917	17	43723189	A	G	-0.0432939	0.00858522	4.59E-07	6.97E-07	0.484854
		Prefrontal Cortex eQTL	ENSG00000238083	17	44588877	rs2696466	17	44289832	G	A	-0.0902575	0.0151182	2.37E-09	1.22E-08	0.04836808
		Putamen Basal Ganglia eQTL	ENSG00000238083	17	44588877	rs55942528	17	43932028	G	A	-0.0445283	0.00884181	4.75E-07	2.72E-06	0.7963627
		Skeletal Muscle eQTL	ENSG00000238083	17	44588877	rs4510068	17	44184828	T	G	-0.0624675	0.0102461	1.08E-09	1.15E-07	0.4008675
	AD	Whole Brain eQTL	ENSG00000238083	17	44610946	rs55825513	17	44176215	A	G	-0.0406017	0.0076394	1.07E-07	4.35E-08	0.02762345
		Amygdala eQTL	ENSG00000238083	17	44588877	rs2942166	17	43715427	C	T	-0.233466	0.0328069	1.11E-12	7.92E-11	0.3625708
		Basal Ganglia eQTL	ENSG00000238083	17	46511511	rs199530	17	44836653	G	A	-0.204835	0.0316577	9.78E-11	5.01E-10	0.01035425
		Frontal Cortex BA9 eQTL	ENSG00000238083	17	44588877	rs4074462	17	43855228	T	G	-0.206808	0.027368	4.14E-14	8.74E-12	0.4723248
		Hippocampus eQTL GTEx	ENSG00000238083	17	44588877	rs2942166	17	43715427	C	T	-0.216414	0.0290959	1.02E-13	1.43E-11	0.3236888
		Liver eQTL	ENSG00000238083	17	44588877	rs58879558	17	44095467	C	T	-0.273497	0.0391198	2.72E-12	1.24E-10	0.4735931
		Multi Ancestry Whole Brain Meta-analysis eQTL	ENSG00000238083	17	44588877	rs2532329	17	44350090	A	G	-0.22263	0.0255896	3.32E-18	1.22E-15	0.4841153
		Putamen Basal Ganglia eQTL	ENSG00000238083	17	44588877	rs9897399	17	43804317	G	A	-0.209757	0.027917	5.75E-14	1.07E-11	0.7115251
		Spinalcord eQTL	ENSG00000238083	17	46511511	rs58879558	17	44095467	T	C	-0.223417	0.0357817	4.27E-10	4.69E-09	0.2679779
LRRC37A2	PD	Substantia nigra eQTL	ENSG00000238083	17	44588877	rs55974014	17	43757450	A	C	-0.208719	0.0314696	3.30E-11	1.61E-09	0.5630028
		Anterior Cingulate Cortex BA24 eQTL	ENSG00000225190	17	43513266	rs55925547	17	43556807	C	T	-0.565533	0.120734	2.81E-06	1.03E-06	0.3394045
		Caudate Basal Ganglia eQTL	ENSG00000225190	17	43513266	rs79172804	17	43828764	A	G	-0.634594	0.119171	1.01E-07	2.22E-07	0.04643205
		Cortex eQTL GTEx	ENSG00000225190	17	43513266	rs10445367	17	43932798	T	G	-0.450407	0.0891061	4.31E-07	3.81E-09	0.03471772
		Cortex eQTL metaBrain	ENSG00000225190	17	45490749	rs55925547	17	43556807	T	C	-0.880801	0.155923	1.61E-08	2.82E-08	0.01207896
		Frontal Cortex BA9 eQTL	ENSG00000225190	17	43513266	rs79172804	17	43828764	A	G	-0.617712	0.125824	9.14E-07	1.19E-06	0.05384652
		Prefrontal Cortex eQTL	ENSG00000225190	17	43568110	rs79724577	17	43463493	C	A	1.20902	0.207821	5.97E-09	5.90E-09	0.01561697
		Putamen Basal Ganglia eQTL	ENSG00000225190	17	43513266	rs62065442	17	43563894	C	T	-0.545119	0.106568	3.13E-07	2.87E-07	0.03026128
		Skeletal Muscle eQTL	ENSG00000225190	17	43513266	rs12947718	17	43493101	A	G	-0.667265	0.0974424	7.50E-12	7.76E-12	0.03535971
	PD	Whole Brain eQTL	ENSG00000225190	17	43540690	rs113434679	17	44126765	A	C	0.545968	0.111957	1.08E-06	7.17E-07	0.3130195
PLEKHM1	PSP	Anterior Cingulate Cortex BA24 eQTL	ENSG00000225190	17	43513266	rs11012	17	43513441	T	C	3.97818	0.768359	2.25E-07	8.50E-08	0.487497
		Cerebellar Hemisphere eQTL	ENSG00000178226	16	31150246	rs78924645	16	31154358	A	G	-0.066351	0.0135831	1.04E-06	1.04E-06	0.5743859
		Cortex eQTL GTEx	ENSG00000178226	16	31150246	rs1549299	16	31154146	A	G	-0.0643026	0.012623	3.50E-07	1.83E-06	0.4465604
		Cortex eQTL metaBrain	ENSG00000178226	16	31150066	rs55667375	16	31155458	C	T	-0.0957689	0.0165264	6.84E-09	2.42E-06	0.4249718
		Multi Ancestry Whole Brain Meta-analysis eQTL	ENSG00000178226	16	31150246	rs78924645	16	31154358	A	G	-0.0889382	0.0151209	4.06E-09	2.10E-06	0.0123253
		Whole Brain eQTL	ENSG00000178226	16	31155830	rs1549299	16	31154146	A	G	-0.0732105	0.0149537	9.79E-07	9.79E-07	0.1419257
			cg07078430	16	31154357	rs78924645	16	31154358	A	G	-0.0865634	0.0176667	9.59E-07	9.59E-07	0.04135834
	AD	Whole Brain meta-analysis mQTL	cg00915858	16	31154636	rs55667375	16	31155458	C	T	0.0928108	0.0167849	3.21E-08	6.46E-07	0.01582905
PRSS36	PD	Whole Brain meta-analysis mQTL	cg00915858	16	31154636	rs55667375	16	31155458	C	T	0.137373	0.0357248	1.20E-04	2.18E-06	0.1779642
	AD	Cerebellum eQTL	ENSG00000185294	17	43922256	rs17573607	17	44083081	A	G	-0.071854	0.0146818	9.88E-07	2.71E-06	0.9201365
SPPL2C	PD	Prefrontal Cortex eQTL	ENSG00000185294	17	43922256	rs17577369	17	44192923	G	A	1.04066	0.182771	1.24E-08	1.30E-07	0.01842581
	LBD	Whole Blood mQTL	cg18586891	4	952366	rs11248059	4	960331	G	C	0.219928	0.0775702	4.58E-03	2.88E-06	0.06025584
		Cerebellum eQTL	ENSG00000127419	4	926175	rs73211813	4	975238	T	C	-0.275567	0.0574718	1.63E-06	7.71E-07	0.3303648
			cg01437235	4	939027	rs935977	4	943540	A	G	-0.183139	0.0360859	3.87E-07	7.83E-10	0.01008769
			cg10941006	4	942705	rs11724898	4	935933	A	G	-0.343495	0.0745804	4.11E-06	1.05E-06	0.01186439
		Whole Blood mQTL	cg02070118	4	952128	rs11248057	4	906131	G	C	0.495391	0.0721195	6.46E-12	4.33E-11	0.6479261
			cg15105011	4	940614	rs10008187	4	941017	T	C	-0.0888259	0.0169041	1.48E-07	4.41E-10	0.4333231
			cg23939001	4	940644	rs6848199	4	940838	T	C	-0.0868987	0.0165618	1.55E-07	1.17E-09	0.2607437
			cg20814179	4	940893	rs10008187	4	941017	T	C	-0.0876292	0.0166691	1.46E-07	6.69E-10	0.3099254
TMEM175	PD	Whole Brain meta-analysis mQTL	cg08222618	4	941054	rs10008187	4	941017	T	C	-0.0869293	0.0165502	1.50E-07	1.40E-09	0.2163939
	AD	Tibial Nerve eQTL	ENSG00000108379	17	44839872	rs199523	17	44848517	A	C	-0.0919719	0.0204807	7.10E-06	2.44E-06	0.03239027
		Cortex eQTL metaBrain	ENSG00000108379	17	46833058	rs9904865	17	44908263	T	C	-0.062473	0.0240469	9.38E-03	1.37E-08	0.09647706
WNT3	PD	Skeletal Muscle eQTL	ENSG00000108379	17	44839872	rs199523	17	44848517	A	C	-0.550915	0.105743	1.89E-07	8.52E-07	0.01487822

S9 - Known Class Genes

Omic	Disease	Gene	probeID	ProbeChr	ProbeBp	topRSID	topSNPChr	topSNPBp	A1	A2	beta, SMR	SE, SMR	p, SMR	p, multi-SNP SMR	p, HEIDI
Whole Brain meta-analysis mQTL	AD	MAPT	cg17569492	17	44026659	rs17650872	17	44039516	T	G	-0.0814127	0.0161079	4.32E-07	4.32E-07	0.6403735
Whole Brain meta-analysis mQTL	AD	MAPT	cg05721485	17	44071124	rs17651483	17	44058861	A	C	-0.0392778	0.00714939	3.93E-08	1.26E-06	0.1842054
Whole Brain meta-analysis mQTL	PD	MAPT	cg07163735	17	43971906	rs55780945	17	44040120	T	C	-0.485081	0.0918691	1.29E-07	1.29E-07	0.2039012
Whole Brain meta-analysis mQTL	PD	MAPT	cg21327887	17	43971928	rs56189701	17	43987801	C	T	-0.428248	0.0761027	1.83E-08	1.83E-08	0.3550347
Whole Brain meta-analysis mQTL	PD	MAPT	cg10780632	17	43973522	rs9303523	17	43976684	C	T	-0.190159	0.0331328	9.51E-09	1.65E-10	0.06329188
Whole Brain meta-analysis mQTL	PD	MAPT	cg19156875	17	43974446	rs17651507	17	44059010	A	T	0.232089	0.0584561	7.18E-05	1.83E-07	0.01714676
Whole Brain meta-analysis mQTL	PD	MAPT	cg20888521	17	43977917	rs17652121	17	44073973	C	T	-0.157605	0.0173742	1.18E-19	4.13E-13	0.02329478
Whole Brain meta-analysis mQTL	PD	MAPT	cg22291189	17	43978251	rs17652121	17	44073973	C	T	0.157605	0.0173742	1.18E-19	4.73E-14	0.04416005
Whole Brain meta-analysis mQTL	PD	MAPT	cg26019600	17	43978704	rs62063859	17	44078616	A	G	0.53072	0.106841	6.79E-07	6.79E-07	0.1094086
Whole Brain meta-analysis mQTL	PD	MAPT	cg12633764	17	43978756	rs62063859	17	44078616	A	G	0.395332	0.0645481	9.09E-10	1.72E-09	0.1586649
Whole Brain meta-analysis mQTL	PD	MAPT	cg05772917	17	44027251	rs17650872	17	44039516	T	G	-0.174153	0.0191833	1.10E-19	1.72E-13	0.01447168
Whole Brain meta-analysis mQTL	PD	MAPT	cg21705961	17	44060775	rs3785884	17	44057595	A	G	0.36317	0.0597238	1.20E-09	1.20E-09	0.1378246
Whole Brain meta-analysis mQTL	PD	MAPT	cg07368061	17	44090862	rs17574361	17	44108202	G	A	0.173968	0.0190538	6.83E-20	2.72E-13	0.06000518
Whole Brain meta-analysis mQTL	PSP	MAPT	cg10224600	17	43975063	rs1981997	17	44056767	A	G	1.14017	0.0815642	2.10E-44	2.10E-44	0.01425494
Whole Brain meta-analysis mQTL	PSP	MAPT	cg20888521	17	43977917	rs1981997	17	44056767	A	G	1.14017	0.0815642	2.10E-44	1.19E-31	0.08905337
Whole Blood mQTL	AD	MAPT	cg23202277	17	43971911	rs17689882	17	43906828	A	G	-0.130387	0.026718	1.06E-06	1.80E-06	0.04451087
Whole Blood mQTL	AD	MAPT	cg00891649	17	43972573	rs111751251	17	44042951	T	C	0.0760692	0.0144379	1.37E-07	3.08E-07	0.07318684
Whole Blood mQTL	AD	MAPT	cg18878992	17	43974344	rs17689882	17	43906828	A	G	-0.0678265	0.0127858	1.13E-07	3.97E-07	0.0158721
Whole Blood mQTL	AD	MAPT	cg14202850	17	43974869	rs117646503	17	43893260	C	T	-0.128522	0.0263587	1.08E-06	6.88E-07	0.2207176
Whole Blood mQTL	AD	MAPT	cg24801230	17	43978533	rs111751251	17	44042951	T	C	0.0343863	0.00628708	4.52E-08	1.81E-06	0.03870612
Whole Blood mQTL	AD	MAPT	cg18228076	17	43983362	rs111751251	17	44042951	T	C	0.0369295	0.00676406	4.77E-08	9.45E-07	0.02969893
Whole Blood mQTL	AD	MAPT	cg02228913	17	44058016	rs112572874	17	44072984	G	A	0.0385404	0.00711207	5.99E-08	2.08E-06	0.02802182
Whole Blood mQTL	AD	MAPT	cg00846647	17	44060252	rs111751251	17	44042951	T	C	0.0461684	0.00851495	5.89E-08	6.76E-07	0.04804293
Whole Blood mQTL	AD	MAPT	cg21705961	17	44060775	rs17689882	17	43906828	A	G	0.0721308	0.0136509	1.26E-07	2.53E-07	0.02723276
Whole Blood mQTL	AD	MAPT	cg01934064	17	44064242	rs2532233	17	44273218	T	C	-0.0982497	0.0191463	2.87E-07	4.34E-07	0.4999633
Whole Blood mQTL	AD	MAPT	cg07368061	17	44090862	rs62056790	17	43975417	A	G	0.0645877	0.0119984	7.32E-08	3.12E-07	0.09065659
Whole Blood mQTL	AD	MAPT	cg09764761	17	44105544	rs111541901	17	43994358	T	C	0.0724373	0.0134753	7.63E-08	2.69E-07	0.07306081
Whole Blood mQTL	PD	MAPT	cg23202277	17	43971911	rs112995313	17	43795768	C	T	-0.558183	0.0820831	1.04E-11	2.08E-10	0.1171783
Whole Blood mQTL	PD	MAPT	cg11909912	17	43974919	rs112197756	17	44154105	G	A	-1.03241	0.206365	5.65E-07	5.55E-07	0.1464477
Whole Blood mQTL	PD	MAPT	cg10224600	17	43975063	rs112197756	17	44154105	G	A	-0.789168	0.132521	2.60E-09	3.65E-08	0.2790191
Whole Blood mQTL	PSP	MAPT	cg05772917	17	44027251	rs11012	17	43513441	T	C	5.23144	0.831232	3.10E-10	6.22E-11	0.1366316
Skeletal Muscle eQTL	PD	ADORA2B	ENSG00000170425	17	15848231	rs1045599	17	15879910	T	C	0.0810002	0.0161604	5.38E-07	2.73E-06	0.0173546
Whole Blood eQTL eQTLgen	AD	KCNN4	ENSG00000104783	19	44278047	rs56681946	19	44283031	C	T	-0.0500123	0.0145726	0.0005992623	1.01E-08	0.1526556

S10 - Novel Genes Networks

Gene	Diseases	Omics	Number of Omics	Network Partners	Number of Network Partners	Potential network toxicity issues	Gene	Diseases
ADAM10	AD	Whole Brain meta-analysis mQTL, Whole Blood eQTL eQTLgen	2	CDH1, **NOTCH1**, **ERBB2**, **SP1**, **CD44**, ELAVL1, **EGF**, ELAVL4, ELAVL2, ELAVL3	10	None in immediate network	ADAM10	AD
SNCA	LBD	Whole Brain meta-analysis mQTL, Whole Blood mQTL, Whole Blood eQTL eQTLgen	3	**SYK, PPP2CB, CADPS2, PLK1, PRKN, CSNK1A1, PPP2CA, PLK3, DYRK1A, GRK2**	10	None in immediate network	SNCA	LBD
EGFR	AD	Whole Blood mQTL	2	**NCK1, LRRFIP1, MUC1, GALNT8, HGS, CAMK2A, PTPRJ, MAPK3, ADAM17, SOX2**	10	None in immediate network	POU5F1	AD
POU5F1	AD	Cortex eQTL metaBrain, Prefrontal Cortex eQTL	1	**DKK1, MAPK1, AKT1, NANOG, AKT2, MAPK3, LEFTY2, THY1, EOMES, HLX**	10	None in immediate network	EGFR	AD
STK39	PD	Whole Brain meta-analysis mQTL, Whole Blood mQTL	2	**MAPK14, CFTR, SLC4A4, WNK4, WNK1, SLC12A2, AATK, SLC12A3, SLC12A1**	9	None in immediate network	STK39	PD
INPP5D	AD	Whole Blood mQTL, Whole Blood eQTL eQTLgen, Whole Blood eQTL GTEx	3	**SHC1, ARRB2, PLCG2, SYK, TRAF6, GRB2**	6	None in immediate network	INPP5D	AD
CRHR1	PD, PSP, AD	Whole Brain meta-analysis mQTL, Whole Blood mQTL, Cortex eQTL metaBrain, Skeletal Muscle eQTL	4	**GNAS, GNAQ, CRH, GNAI1, POMC**	5	None in immediate network	CRHR1	PD, PSP, AD
APH1B	AD	Cortex eQTL metaBrain, Tibial Nerve eQTL, Skeletal Muscle eQTL, Whole Blood eQTL eQTLgen	4	**PSEN2, NCSTN, PSEN1, PSENEN**	4	None in immediate network	APH1B	AD
MINK1	AD	Whole Blood eQTL eQTLgen	1	**KRAS, KCNH2, PRICKLE1**	3	None in immediate network	MINK1	AD
CLU	AD	Whole Brain meta-analysis mQTL	1	MYBL2, **GDNF**, LRP2	3	None in immediate network	CLU	AD
CR1	AD	Cerebellum eQTL, Basal Ganglia eQTL, Cortex eQTL metaBrain, Cortex eQTL GTEx, Caudate Basal Ganglia eQTL, Whole Brain eQTL	6	C5AR1, **C5AR2**	2	None in immediate network	CR1	AD
ACE	AD	Whole Blood eQTL eQTLgen	6	CSNK2A1, **AGT**	2	None in immediate network	ITGAX	PD
CD38	PD	Cerebellum eQTL, Whole Brain meta-analysis mQTL, Whole Blood mQTL, Cerebellar Hemisphere eQTL, Cortex eQTL GTEx, Tibial Nerve eQTL, Skeletal Muscle eQTL, Hypothalamus eQTL, Whole Blood eQTL eQTLgen, Whole Brain eQTL, Cerebellum eQTL	6	**OXT, PECAM1**	2	OXT - ALS	KAT8	PD, AD
RABEP1	AD	Whole Blood eQTL eQTLgen	1	**RAB5A, RABGEF1**	2	None in immediate network	ERCC2	AD
ERCC2	AD	Cerebellum eQTL, Whole Blood mQTL, Cortex eQTL metaBrain, Prefrontal Cortex eQTL, Whole Blood eQTL eQTLgen, Whole Brain eQTL	1	**ERCC3, ERCC5**	2	None in immediate network	ACE	AD
KAT8	PD, AD	Whole Blood eQTL eQTLgen	11	KMT2A, **FASN**	2	None in immediate network	RABEP1	AD
ITGAX	PD	Cortex eQTL metaBrain, Prefrontal Cortex eQTL, Cortex eQTL GTEx, Caudate Basal Ganglia eQTL, Whole Brain eQTL, Nucleus Accumbens Basal Ganglia	1	FGA, **ICAM1**	2	None in immediate network	CD38	PD
GAK	PD	Whole Blood mQTL, Skeletal Muscle eQTL	2	**SRC**	1	None in immediate network	STX4	PD
STX4	PD	Whole Blood mQTL	2	STXBP4	1	None in immediate network	EPHA1	AD
EPHB4	AD	Whole Blood eQTL eQTLgen	1	EFNB1	1	None in immediate network	EPHB4	AD
EPHA1	AD	Whole Brain meta-analysis mQTL, Whole Brain eQTL	1	**EFNA1**	1	None in immediate network	GAK	PD
GPNMB	PD	Skeletal Muscle eQTL, Whole Blood eQTL eQTLgen	14	No curated network partners	0	None in immediate network	VKORC1	PD
STAG3	AD	Whole Blood mQTL	14	No curated network partners	0	None in immediate network	CDSN	AD
CHRNE	AD	Whole Brain meta-analysis mQTL	2	No curated network partners	0	None in immediate network	MAT1A	AD
NDUFS2	AD	Whole Blood mQTL	4	No curated network partners	0	None in immediate network	SLC44A4	AD
FCER1G	AD	Whole Brain meta-analysis mQTL	3	No curated network partners	0	None in immediate network	EPHX2	AD
VKORC1	PD	Whole Blood mQTL	2	No curated network partners	0	None in immediate network	PSORS1C1	AD
DNTT	AD	Cerebellum eQTL, Whole Brain meta-analysis mQTL, Cortex eQTL metaBrain, Frontal Cortex BA9 eQTL, Prefrontal Cortex eQTL, Cortex eQTL GTEx, Caudate Basal Ganglia eQTL, Multi Ancestry Whole Brain Meta-analysis eQTL, Hypothalamus eQTL, Anterior Cingulate Cortex BA24 eQTL, Whole Blood eQTL eQTLgen, Putamen Basal Ganglia eQTL, Whole Brain eQTL, Nucleus Accumbens Basal Ganglia	1	No curated network partners	0	None in immediate network	GPNMB	PD
CKM	AD	Whole Brain meta-analysis mQTL	1	No curated network partners	0	None in immediate network	MS4A2	AD
HSD3B7	PD	Whole Brain meta-analysis mQTL, Whole Blood mQTL, Cortex eQTL metaBrain, Frontal Cortex BA9 eQTL, Cerebellar Hemisphere eQTL, Caudate Basal Ganglia eQTL, Tibial Nerve eQTL, Hippocampus eQTL, Substantia nigra eQTL, Hypothalamus eQTL, Whole Blood eQTL eQTLgen, Amygdala eQTL, Cerebellum eQTL, Nucleus Accumbens Basal Ganglia	2	No curated network partners	0	None in immediate network	STAG3	AD
BST1	PD	Whole Blood mQTL	1	No curated network partners	0	None in immediate network	MICB	AD
STX1B	PD	Whole Blood mQTL	1	No curated network partners	0	None in immediate network	STX1B	PD
PSMC3	AD	Whole Blood mQTL	1	No curated network partners	0	None in immediate network	PSMC3	AD
CDSN	AD	Whole Blood mQTL, Cortex eQTL GTEx	1	No curated network partners	0	None in immediate network	CHRNE	AD
MICB	AD	Whole Blood eQTL eQTLgen	1	No curated network partners	0	None in immediate network	BST1	PD
MS4A2	AD	Prefrontal Cortex eQTL, Skeletal Muscle eQTL	1	No curated network partners	0	None in immediate network	HSD3B7	PD
PSORS1C1	AD	Whole Blood mQTL, Cortex eQTL metaBrain, Whole Blood eQTL eQTLgen, Whole Brain eQTL	1	No curated network partners	0	None in immediate network	NDUFS2	AD
EPHX2	AD	Whole Blood eQTL eQTLgen	1	No curated network partners	0	None in immediate network	CKM	AD
SLC44A4	AD	Skeletal Muscle eQTL, Whole Blood eQTL eQTLgen, Whole Blood eQTL GTEx	1	No curated network partners	0	None in immediate network	FCER1G	AD
MAT1A	AD	Whole Blood eQTL eQTLgen	1	No curated network partners	0	None in immediate network	DNTT	AD
FBXL19	PD	Whole Blood mQTL	1	No curated network partners	0	None in immediate network	FBXL19	PD

Source	Ancestry	rsID	Allele1	Allele2	Effect	P-value
Wu et al. (2017)	Taiwanese	rs3793947	A	*n/a*	*n/a*	0.508
Xu et al. (2016)	Chinese	rs156429	A	A	*n/a*	0.108
Rizig et al. (under preperation)	African & African Admix	rs1637190	G	A	-0.0808	0.1338
		rs858275	C	T	-0.0824	0.125
		rs858274	C	T	-0.0522	0.5875
		rs858239	G	A	0.0494	0.6099
		rs199357	G	A	-0.0271	0.6602
		rs199355	G	A	-0.0122	0.8008
		rs199348	C	A	-0.0042	0.931
		rs199351	C	A	-0.0335	0.7277
		rs466240	C	T	-0.0232	0.8135
		rs858273	C	T	0.054	0.5768
Present Study	multiancestry	rs858275	T	C	-0.107745	1.08E-08
	European	rs1637190	A	G	0.124869	2.00E-07
		rs199357	A	G	0.118241	9.66E-07
		rs858239	A	G	0.24504	1.94E-08
		rs199357	A	G	0.126337	2.20E-06
		rs858273	T	C	0.128654	2.65E-06
		rs199351	C	A	0.132703	2.88E-06
		rs858273	T	C	0.155196	1.40E-06
		rs858274	T	C	0.0887275	3.21E-08
		rs199357	A	G	0.179231	1.88E-08
		rs199348	C	A	0.117214	1.61E-08
		rs199355	G	A	-0.237456	1.94E-06
		rs199357	G	A	-0.181814	2.46E-07
		rs199357	G	A	-0.17763	5.79E-07
		rs466240	T	C	-0.078348	2.84E-08
		rs199357	G	A	-0.0900894	1.89E-08
		rs199357	G	A	-0.0901975	2.45E-08
		rs199357	A	G	0.131601	1.20E-06
		rs199357	A	G	0.121781	8.08E-07
